# Electroactive Electrospun Nanofibrous Scaffolds: Innovative Approaches for Improved Skin Wound Healing

**DOI:** 10.1002/advs.202416267

**Published:** 2025-04-07

**Authors:** Yang Zhang, Zhiyuan Zheng, Shilu Zhu, Liang Xu, Qingdong Zhang, Jie Gao, Min Ye, Shuwei Shen, Jinyu Xing, Ming Wu, Ronald X. Xu

**Affiliations:** ^1^ Department of Rehabilitation The First Affiliated Hospital of USTC Division of Life Sciences and Medicine University of Science and Technology of China Hefei Anhui 230027 P. R. China; ^2^ Department of Precision Machinery and Instrumentation School of Engineering Science University of Science and Technology of China Hefei Anhui 230027 P. R. China; ^3^ School of Biomedical Engineering Division of Life Sciences and Medicine University of Science and Technology of China Hefei Anhui 230027 P. R. China; ^4^ Suzhou Institute for Advanced Research University of Science and Technology of China Suzhou 215000 China

**Keywords:** bioelectricity, electroactive biomaterials, electrospun nanofiber, scaffold, wound healing

## Abstract

The incidence and burden of skin wounds, especially chronic and complex wounds, have a profound impact on healthcare. Effective wound healing strategies require a multidisciplinary approach, and advances in materials science and bioengineering have paved the way for the development of novel wound healing dressing. In this context, electrospun nanofibers can mimic the architecture of the natural extracellular matrix and provide new opportunities for wound healing. Inspired by the bioelectric phenomena in the human body, electrospun nanofibrous scaffolds with electroactive characteristics are gaining widespread attention and gradually emerging. To this end, this review first summarizes the basic process of wound healing, the causes of chronic wounds, and the current status of clinical treatment, highlighting the urgency and importance of wound dressings. Then, the biological effects of electric fields, the preparation materials, and manufacturing techniques of electroactive electrospun nanofibrous (EEN) scaffolds are discussed. The latest progress of EEN scaffolds in enhancing skin wound healing is systematically reviewed, mainly including treatment and monitoring. Finally, the importance of EEN scaffold strategies to enhance wound healing is emphasized, and the challenges and prospects of EEN scaffolds are summarized.

## Graphical abstract

This review explores the potential of electroactive electrospun nanofibrous (EEN) scaffolds for enhanced skin wound healing. It discusses how a variety of electroactive materials can be prepared into EEN scaffolds via electrospinning technology, and their applications in various wound types. The review provides insights into the future perspectives for skin tissue regeneration and repair, and the development of novel high‐performance EEN.

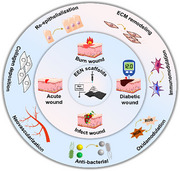

## 1 Introduction

The incidence and burden of cutaneous wounds have a profound impact on healthcare, with chronic non‐healing wounds imposing considerable demands on the human and material resources of patients, healthcare providers, and systems.^[^ ^1^ ^]^ With an aging population, the global rise in diabetes and obesity, and the ongoing threat of infections, the challenge of wound healing—particularly chronic wounds—will continue to be a significant clinical, social, and economic concern.^[^ ^2^ ^]^ Consequently, there is an urgent need for more effective and cost‐efficient wound treatment, along with innovative strategies to accelerate the wound healing process, particularly in the case of chronic wounds.

To date, numerous wound healing strategies have been developed, including hyperbaric oxygen therapy, negative pressure therapy, physical factor therapy, cellular therapy, engineered skin grafts, and topical drug or biologic delivery.^[^ ^3^ ^]^ Despite the potential of these approaches, challenges such as high costs, uncertain efficacy, unclear biosafety, and dependence on laboratory‐based products continue to make the treatment of both acute and chronic wounds difficult.^[^ ^4^ ^]^

As we know, wound dressings play a crucial and irreplaceable role, regardless of the type of wound or therapeutic strategy adopted.^[^ ^5^ ^]^ Currently, the most commonly used clinical wound dressings consist of traditional materials such as cotton, bandages, and gauze.^[^ ^6^ ^]^ However, these materials primarily serve to protect the wound from contaminants, play only a passive role in the healing process, and are often difficult to remove due to adhesions caused by prolonged contact, leading to secondary damage and hindering wound healing.^[^ ^7^ ^]^ For this reason, growing research efforts are focused on overcoming the limitations of conventional dressings and exploring novel materials for wound healing, including films, foams, sponges, hydrogels, and nanofiber membranes.^[^ ^7^, ^8^ ^]^ Among these strategies, electrospun nanofiber films stand out due to their high specific surface area, microporosity, robust drug‐carrying capacity, controlled release properties, and their ability to mimic the extracellular matrix (ECM), making them highly promising for wound dressings.^[^ ^9^, ^10^ ^]^ Although electrospun nanofiber wound dressings have been widely shown to promote healing and regeneration of damaged skin to some extent, their functional restoration and regeneration efficiencies remain suboptimal, particularly for large acute wounds, infected wounds, and various chronic wounds.^[^ ^11^ ^]^ Therefore, the development of stimuli‐responsive and functionalized nanofibers has garnered significant interest, including the introduction of biochemical cues^[^ ^12^, ^13^ ^]^ (e.g., growth factors, small molecules, and other bioactive agents) and biophysical cues^[^ ^14^, ^15^, ^16^, ^17^ ^]^ (e.g., surface morphology, mechanical, photo‐, and magneto‐electric stimuli) to enhance the wound healing effectiveness of nanofiber membranes.

Bioelectricity is a critical guiding factor in human life processes, and its regulatory effects on cell behavior and tissue function are well documented.^[^ ^18^ ^]^ The therapeutic approach of applying electrical stimulation (ES) to the body, by simulating or augmenting bioelectricity, has been employed for the repair and regeneration of electrically active tissues and organs, including neural,^[^ ^19^ ^]^ musculoskeletal,^[^ ^20^ ^]^ cardiac,^[^ ^21^ ^]^ and skin tissues.^[^ ^22^ ^]^ Endogenous electric fields (EFs), which underpin bioelectrical signaling, play a critical role in wound repair.^[^ ^23^, ^24^ ^]^ Numerous studies have demonstrated that cutaneous wound healing involves cell migration, proliferation, polarization, and vascularization in response to endogenous currents,^[^ ^25^, ^26^, ^27^ ^]^ while clinical research has confirmed the positive effects of electrical stimulation on wound healing.^[^ ^28^, ^29^ ^]^ Nanofibers functionalized with electrical properties possess the potential to integrate physicochemical, electrical, and topological features, effectively mimicking the electrophysiological microenvironment for tissue regeneration across diverse pathological conditions.^[^ ^30^ ^]^ EEN scaffolds have been shown to exert synergistic effects in wound hemostasis, anti‐inflammatory and antibacterial activities, angiogenesis, epithelialization, and scar remodeling.^[^ ^31^, ^32^, ^33^, ^34^, ^35^, ^36^ ^]^ Furthermore, EEN scaffolds can trigger drug release under external electrical stimulation to promote wound healing and integrate biosensors to monitor the healing process.^[^ ^37^, ^38^ ^]^

**Figure 1:**
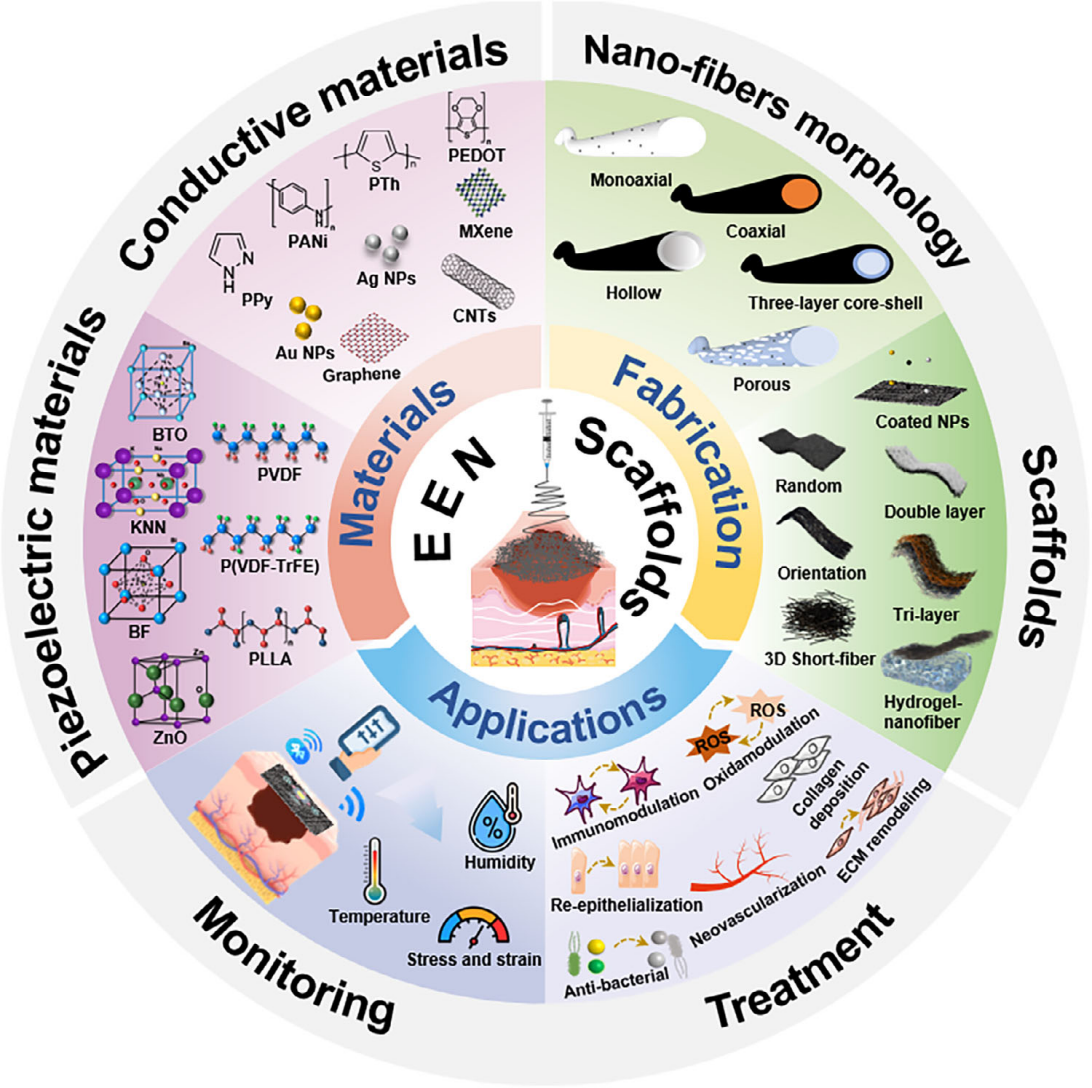
Schematic illustration of the main sections in this review, focusing on electroactive materials, preparation techniques, and potential applications in wound healing.

EEN scaffolds offer new opportunities for cutaneous wound healing, driving advancements in skin tissue repair and regeneration while providing technical support for tissue engineering and regenerative medicine. Although several studies have explored electrospun nanofibrous scaffolds in wound healing and skin tissue engineering,^[^ ^39^, ^40^, ^41^, ^42^ ^]^ a comprehensive review of EEN scaffolds for enhancing skin wound healing remains lacking. Herein, we provide a review article to summarize the design and research progress of EEN for enhanced skin wound healing (**Figure** **1**). In this review, we first summarize the wound healing process, mechanisms, causes of chronic wounds, and current status of clinical treatments, highlighting the importance of wound dressings. We then discuss the biological effects of EFs and summarize their intrinsic properties and the latest progress in wound healing. Next, the preparation materials (e.g., conductive biomaterials and piezoelectric biomaterials) and fabrication techniques (e.g., blend, aligned, core‐shell, and hollow fibers) of EEN scaffolds are outlined, as well as their roles in tissue engineering. In the following section, we systematically categorize and discuss the applications of EEN scaffolds to enhance skin wound healing, including conventional full‐thickness incisions, diabetic wounds, infected wounds, and wound monitoring. Finally, we describe the challenges and future perspectives of EEN scaffolds in terms of the electrical stimulation healing mechanism, electrical parameters, biosafety, wound models, and intelligent strategies. This review, which covers EFs effects, electroactive materials, scaffold structures, various wound applications, and other relevant aspects, is expected to provide valuable insights into the development of novel high‐performance EEN scaffolds.

## 2 Physiology of Skin and Its Wound Healing Mechanisms

A comprehensive understanding of the normal structural and functional characteristics of the skin not only provides a theoretical foundation for treating skin diseases but also offers critical insights for constructing in vitro skin models and advancing the innovative development of skin grafts and tissue‐engineered scaffolds. Therefore, a brief overview of skin physiology, structure, and function is provided in Section 2.1 of this section. Additionally, the transition from a healthy healing wound to a non‐healing wound may result from various factors that influence the different stages of wound healing. A comprehensive understanding of the pathophysiological events associated with wound healing is essential for developing effective treatment strategies. Therefore, Sections 2.2 and 2.3 of this section provide a detailed discussion of the wound healing process, causes of chronic wound formation, and underlying mechanisms.

### 2.1 Physiological Structure and Function of the Skin

The skin, the largest organ of the human body, plays a vital role in regulating the physiological environment, including thermoregulation, water and electrolyte homeostasis, immune functions (both innate and adaptive), and sensory perception.^[^ ^43^ ^]^ It serves as the primary barrier against external stimuli and pathogens, making it the most vulnerable part of the body to injury.^[^ ^44^ ^]^ Its structure is complex, comprising various subcompartments and cell populations that interact in diverse ways to establish and maintain this barrier.^[^ ^45^ ^]^ Mammalian skin is primarily composed of the epidermis, dermis, and subcutaneous adipose tissue, along with various appendages.^[^ ^46^ ^]^ The epidermis and dermis, derived from the ectoderm and mesoderm respectively, are the two primary layers of the skin that are tightly connected, with a combined thickness varying from ≈0.5 to 4 mm depending on the body part.^[^ ^47^ ^]^ Each layer of the skin varies in structure, biochemical composition, water content, as well as physical and mechanical properties.^[^ ^45^ ^]^

The epidermis, the outermost layer of the skin, is composed of four sublayers, listed from outer to inner: stratum corneum, stratum granulosum, stratum spinosum, and stratum basale.^[^ ^48^ ^]^ Keratinocytes, which constitute at least 80% of the total cells, are primarily located in the stratum corneum and secrete lipid components in the stratum granulosum.^[^ ^49^ ^]^ Langerhans cells, which play a role in immune function, are the most prominent in the stratum spinosum.^[^ ^50^ ^]^ Melanocytes, responsible for melanin production,^[^ ^51^ ^]^ and Merkel cells, involved in sensory function, are primarily located near the basal layer.^[^ ^52^ ^]^ Each sublayer has distinct structural and functional roles, yet they function synergistically.^[^ ^53^ ^]^ The stratum corneum, a critical sublayer of the epidermis, provides an effective cellular barrier between the external and internal environments of living cells and, plays a vital role in the skin's defense system, such as protecting against external damage and maintaining internal water balance.^[^ ^49^ ^]^ The cell wall of the stratum corneum is composed of highly cross‐linked proteins, primarily keratin, that are tightly bound to lipids, forming a keratinized lipid envelope.^[^ ^54^, ^55^ ^]^ The epidermis lacks a direct blood supply and relies on the dermis for oxygen and nutrients, primarily through diffusion.^[^ ^53^ ^]^

The dermis, situated beneath the epidermis, is firmly connected to the epidermis at the basement membrane (dermo‐epidermal junction) through a complex network of proteins and glycoproteins, which supply the epidermis with oxygen‐rich blood and facilitate waste removal.^[^ ^47^, ^56^ ^]^ The dermis contains resident cell types, including fibroblasts and immune cells.^[^ ^57^ ^]^ Fibroblasts primarily produce the ECM, composed of collagen, elastin, and proteoglycans, which provide elasticity and mechanical properties (collage and elastin) and maintain tissue hydration (glycosaminoglycans).^[^ ^46^, ^47^ ^]^ Immune cells in the dermis include macrophages, mast cells, eosinophils, neutrophils, B‐lymphocytes, and T‐lymphocytes, primarily responsible for immunomodulation and infection prevention.^[^ ^58^ ^]^ Additionally, the dermis can be subdivided into two layers: papillary dermis and reticular dermis, each containing distinct fibroblast subpopulations with corresponding functional characteristics. The papillary dermis, located adjacent to the epidermis, is composed of small‐diameter collagen fibers that form a spongy structure, providing the skin with protection from mechanical stress.^[^ ^46^, ^47^, ^59^ ^]^ In contrast, the reticular dermis comprises large‐diameter, densely packed bundles of collagen fibers interwoven to form a robust network, endowing the skin with its elastic mechanical properties and providing structural support.^[^ ^47^, ^59^, ^60^ ^]^ This structural composition not only resists external physical damage but also maintains the dermis' structural integrity and functionality, which is essential for overall skin health.^[^ ^59^ ^]^ Moreover, this layer is a current focus of research in tissue engineering and regenerative medicine, where detailed structural and functional characterization forms the foundation for developing more effective therapeutic and regenerative strategies for the skin.

Subcutaneous adipose tissue also referred to as subcutaneous tissue, is situated in the innermost layer of the skin and acts as a bridge between the dermis and the underlying bones.^[^ ^61^ ^]^ This layer primarily comprises fat cells, which play a critical role in energy storage and thermoregulation.^[^ ^62^ ^]^ It is also rich in stem cells, hormones, and growth factors that contribute to epithelialization and wound healing.^[^ ^63^ ^]^ However, the subcutaneous tissue is often overlooked in in vitro models.

### 2.2 Processes and Mechanisms of Skin Wound Healing

**Figure 2:**
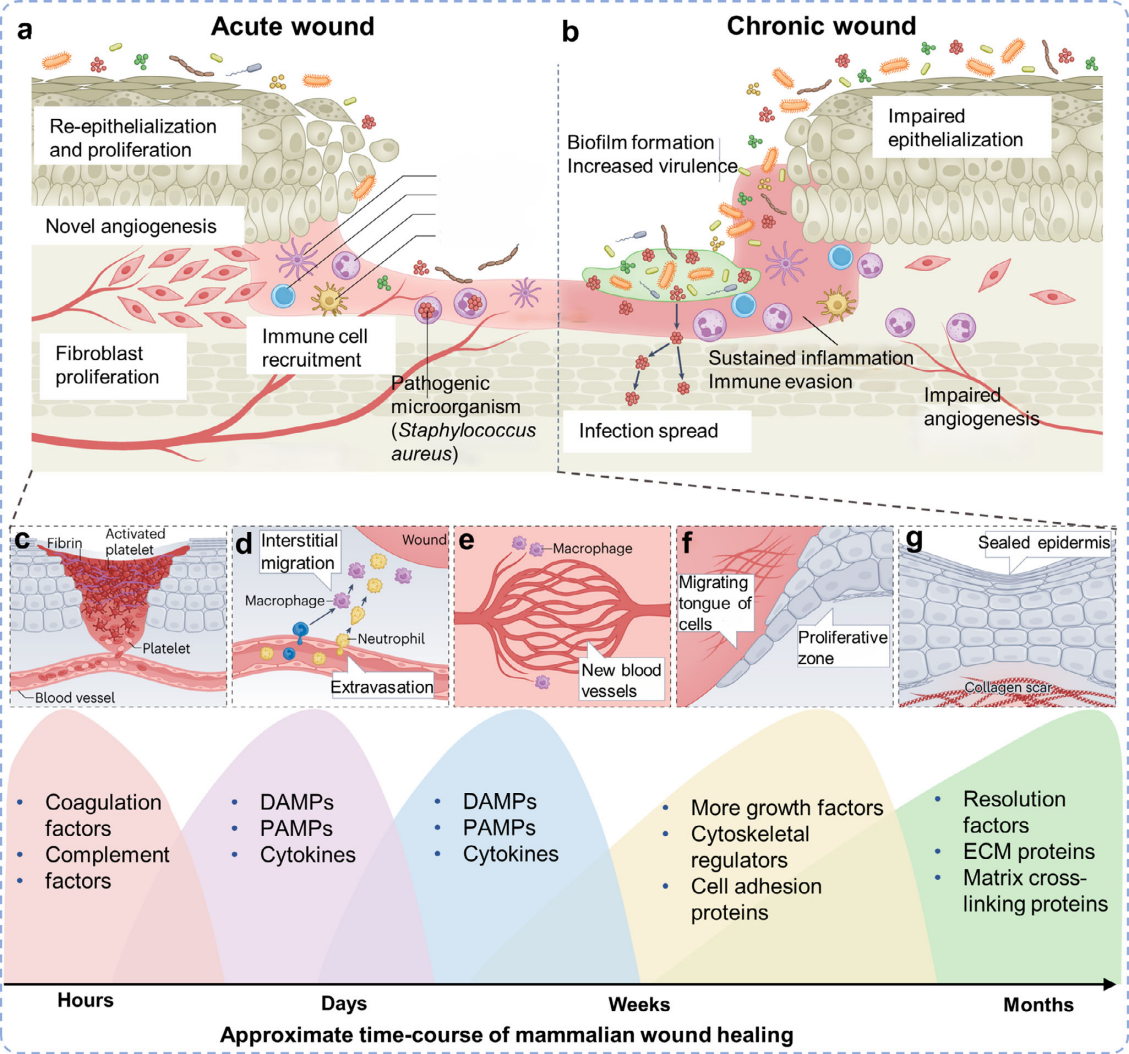
a) Development of acute (left) and b) chronic wounds (right). Reproduced with permission.^[^ ^67^ ^]^ Copyright 2024, Springer Nature. c) Clot formation phase. d) Inflammation phase. e) Angiogenesis phase. f) Migration and proliferation phase. g) Remodeling and resolution phase. Modified with permission.^[^ ^65^ ^]^ Copyright 2024, Springer Nature.

Skin damage resulting from various external factors, including mechanical, physical, thermal, chemical, or radiological injuries, as well as chronic diseases, can significantly impair the structure and function of the skin.^[^ ^56^ ^]^ Wound healing is initiated immediately upon the occurrence of a skin injury. Natural wound healing in the skin is a structured, timely, dynamic, and highly complex process, involving phases of hemostasis, inflammation, angiogenesis, proliferation, and remodeling, typically achieving wound closure in 3–14 days depending on the type of wound (**Figure** **2**a).^[^ ^64^, ^65^, ^66^ ^]^

Certain wounds may become chronic or even non‐healing due to underlying systemic or local factors that disrupt the healing process (Figure 2b).^[^ ^68^ ^]^ The initial response to skin injury (hemostasis) involves rapid closure of the damaged barrier via vasoconstriction and quick formation of blood clots to prevent blood loss and pathogen invasion (Figure 2c).^[^ ^65^ ^]^ This process activates a complex coagulation cascade, in which prothrombin cleaves fibrinogen to fibrin, forming fibrin fibers (reticulated blood clots) to seal the injury.^[^ ^69^, ^70^ ^]^ Platelets release various mediators, such as transforming growth factor‐beta (TGF‐β), platelet‐derived growth factor (PDGF), stromal cell‐derived factor 1 (SDF1, also known as CXCL12), vascular endothelial growth factor (VEGF), and endothelial repressors, to promote wound healing, activate the local immune response, and enhance coagulation through factors secreted by immune cells, primarily macrophages.^[^ ^71^ ^]^ This reciprocal activation and interconnection underscore the fact that wound healing is a highly coordinated and complex biological process.^[^ ^72^ ^]^

The wound enters the inflammatory phase over several days, involving complex coordination of cellular and molecular interactions within the immune system (Figure 2d).^[^ ^65^ ^]^ Innate immune cells, including neutrophils and macrophages, are actively recruited and migrate into the wound via nearby vascular extravasation.^[^ ^65^ ^]^ As the primary leukocytes, these cells serve as the first responders to acute inflammation.^[^ ^73^ ^]^ Neutrophil migration to the wound mesenchyme is mediated by damage‐associated molecular pattern (DAMP) and pathogen‐associated molecular pattern (PAMP) receptors, along with CXC receptors (CXCR).^[^ ^74^, ^75^ ^]^ It plays a pivotal role in infection resistance through multiple mechanisms, including reactive oxygen species (ROS) release and neutrophil extracellular trap (NET) formation.^[^ ^65^, ^76^, ^77^ ^]^ However, excessive NET production can impair receptors, growth factors, and the ECM, impeding the angiogenic process and highlighting the potential dangers of neutrophil overactivity.^[^ ^77^, ^78^, ^79^ ^]^ Timely removal of neutrophils is crucial for wound healing, as delayed clearance can lead to chronic inflammation and impaired healing. For instance, one study found that disrupting DNase 1 of the NET accelerated wound healing in both diabetic and normoglycemic wild‐type (WT) mice.^[^ ^80^ ^]^ Another crucial type of immune cells is macrophages, which primarily differentiate from locally resident and vascular exudate monocytes. In the early stages of inflammation, differentiation into the M1 subtype is induced by damage‐associated molecular patterns and other inflammatory signals produced at the wound site, such as Interferon‐gamma (IFN‐γ) and Tumor Necrosis Factor (TNF). This subtype produces mitochondrial ROS, contributing to pathogen clearance, proinflammatory responses, and early tissue repair.^[^ ^65^, ^81^ ^]^ In the later stages of the inflammatory phase, macrophages transition from the M1 to the M2 phenotype, characterized by interleukin 4 receptor alpha (IL‐4Rα)‐mediated mitochondrial respiration, along with excitatory and pro‐catabolic functions.^[^ ^82^ ^]^ These functions contribute to anti‐inflammation, ECM production, fibroblast proliferation initiation, and angiogenesis.^[^ ^82^ ^]^ Additionally, macrophages act as scavengers, phagocytizing depleted neutrophils, bacteria, and cellular debris, while secreting lipid mediators (e.g., lipoxins) to promote the resolution of inflammation, thus preventing further damage to the wound site during the later stages of healing.^[^ ^83^, ^84^ ^]^ Therefore, this dynamic shift in macrophage phenotype from M1 to M2 is crucial for coordinating the various stages of healing and extends beyond the inflammatory phase. Any delay or disruption in this phenotypic shift can impair wound healing. For instance, macrophages isolated from wounds in diabetic and db/db mice exhibit persistent inflammatory vesicle activity, resulting in impaired early healing in diabetic wounds.^[^ ^85^ ^]^ It has also been found that iron overload in macrophages in chronic venous leg ulcers in humans and a mouse model induces a macrophage population with an uncontrolled pro‐inflammatory M1 activation state, perpetuating inflammation and impairing wound healing.^[^ ^86^ ^]^ Given the crucial role of immune cells in wound healing, numerous tissue engineering studies have focused on enhancing the efficiency and quality of wound healing by modulating immune cell activity.^[^ ^87^, ^88^, ^89^, ^90^, ^91^, ^92^ ^]^

Subsequently, the wound transitions into the proliferative phase, typically lasting several weeks, characterized by granulation tissue formation, angiogenesis, and epithelialization.^[^ ^65^ ^]^ Granulation tissue formation is primarily driven by fibroblasts, although macrophages, endothelial cells, and keratinocytes also play key roles.^[^ ^93^ ^]^ Stimulated by growth factors, particularly transforming growth factor‐beta and PDGF secreted by macrophages, fibroblasts proliferate and migrate to the wound site, synthesizing and depositing various ECM proteins (e.g., hyaluronic acid, fibronectin, collagen, and proteoglycans).^[^ ^94^ ^]^ The ECM formation provides a structural framework for subsequent vascularization and epithelialization.^[^ ^95^ ^]^ Additionally, after adult fibroblasts have deposited sufficient ECM, they undergo a phenotypic transformation, driven by mechanical tension and growth factors, to form highly contractile myofibroblasts characterized by cytoplasmic microfilament bundles of α‐smooth muscle actin (α‐SMA).^[^ ^96^ ^]^ The contractile forces generated by myofibroblasts draw the wound edges together at a rate of up to 0.75 mm per day.^[^ ^97^ ^]^ This process occurs throughout the proliferative phase and is known as wound contraction.^[^ ^98^ ^]^ The underlying mechanism likely involves the intracellular cytoskeleton exerting contractile forces through ECM‐linked adhesion patches; however, excessive contraction may result in abnormal scar formation.^[^ ^98^ ^]^

A critical mechanism that must be rapidly established during granulation tissue formation for proper wound healing is angiogenesis, characterized by the formation of new blood vessels from existing ones, giving the tissue its characteristic pink, swollen appearance (Figure 2e).^[^ ^99^ ^]^ Angiogenesis initiates during the early hemostatic phase with thrombus formation and becomes more pronounced during the proliferative phase.^[^ ^100^ ^]^ Angiogenesis during the proliferative phase is stimulated by various growth factors (e.g., fibroblast growth factor [FGF], VEGF, TGF‐β1, and angiopoietin) and cytokines, driven by changes in the tissue environment, including hypoxia, increased lactate, and decreased pH, initially resulting in the formation of a disorganized vascular network.^[^ ^101^, ^102^ ^]^ As wound healing progresses and capillary density peaks, the expression of several anti‐angiogenic factors increases, such as Sprouty2 and pigment epithelium‐derived factor (PEDF), resulting in the pruning of the vascular network.^[^ ^103^, ^104^ ^]^ Subsequent stabilization of capillaries by pericytes and vascular smooth muscle cells (vSMCs) promotes the maturation and stabilization of the vascular network.^[^ ^105^, ^106^ ^]^ Pericytes play a pivotal role in vascular maturation, as modulatory proteins expressed by wound macrophages activate and differentiate pericytes,^[^ ^107^ ^]^ while elevated levels of platelet‐derived growth factor‐BB (PDGF‐BB) at the wound site further support their recruitment and differentiation.^[^ ^108^ ^]^ Additionally, the activation of the Ang‐2/Tie2 signaling pathway in pericytes also plays a critical role in regulating angiogenesis.^[^ ^109^ ^]^ Considering that vascularization is a crucial physiological process and plays a pivotal role in cutaneous wound healing, numerous studies have explored various strategies, including biomaterials‐based and cellular therapies, to enhance or modulate angiogenesis at the wound site from a tissue engineering perspective.^[^ ^110^, ^111^ ^]^

Following the initial injury, epithelial cells gradually migrate from the wound edge toward the center, covering the newly formed granulation tissue and typically completing epithelialization within 8 to 10 days (Figure 2f).^[^ ^65^ ^]^ During this stage, epithelial cells may acquire motility through the process of epithelial‐mesenchymal transition (EMT).^[^ ^112^ ^]^ Subsequently, contact inhibition halts further epithelial cell migration, enabling them to transition from a motile phenotype to a proliferative one, thereby repopulating and completing the repair of the epidermal barrier.^[^ ^113^, ^114^ ^]^ Notably, an appropriate ECM is a prerequisite for promoting epithelial cell migration and achieving effective epithelialization, highlighting the importance of ECM generated by macrophages, fibroblasts, and blood vessels, as well as components such as type I collagen, glycoproteins, fibronectin, and hyaluronic acid.^[^ ^115^ ^]^

The final stage of wound healing is the remodeling phase, which can persist for months or even years (Figure 2g).^[^ ^72^ ^]^ This phase is characterized by scar maturation, involving the degradation and reorganization of the ECM, with a critical emphasis on maintaining a balance between these processes.^[^ ^116^ ^]^ Type III collagen fibers, rapidly produced by fibroblasts, are gradually replaced by stronger type I collagen fibers through the action of matrix metalloproteinases, thereby enhancing the ultimate tensile strength of the skin.^[^ ^117^ ^]^ However, the final tensile strength of the wound may only be restored to ≈80% of that of the original tissue, making it difficult to achieve the same level of strength as before the injury.^[^ ^118^ ^]^ Simultaneously, during the remodeling phase, there is a gradual reduction in the number of cells that played crucial roles in earlier phases, such as endothelial cells, macrophages, and fibroblasts.^[^ ^119^ ^]^ This reduction is essential for the successful completion of wound healing and the prevention of hyperplastic scar formation.^[^ ^119^ ^]^

Additionally, during wound healing, aside from the epidermal and connective tissue fibroblasts, macrophages, and endothelial cells (ECs) that have been the primary focus, adipocytes, melanocytes, and cutaneous innervation also make important contributions to wound repair.^[^ ^65^ ^]^ However, as this paper focuses on the role of electroactive electrospun fiber scaffolds in the major stages of wound healing, these aspects will not be explored in detail here.

### 2.3 Pathophysiology of Chronic Wounds

Wound healing is a complex, overlapping biological process that involves precise interactions between multiple cell types and signaling molecules.^[^ ^66^, ^120^ ^]^ Each stage is characterized by specific biological activities and goals that must be precisely coordinated, timely executed, and sequentially ordered.^[^ ^65^ ^]^ Any disruption or delay in one of these phases can upset the balance of these interactions, causing the healing process to deviate from the normal progression of successive repair phases, potentially leading to delayed skin healing and the development of chronic wounds.^[^ ^68^ ^]^ Venous ulcers, pressure ulcers, arterial ulcers, and diabetic foot ulcers are the most prevalent types of chronic wounds.^[^ ^121^ ^]^ Furthermore, other wounds, including surgical incisions, traumatic injuries, and thermal injuries, may also progress into chronic wounds.^[^ ^122^ ^]^ The pathophysiology of these chronic wounds is fundamentally similar, characterized by persistent infection and inflammation, excessive ROS production, defective epithelialization, inadequate angiogenesis, and local hypoxia.^[^ ^68^, ^123^ ^]^

Existing literature indicates that the inflammatory response is a critical factor in the formation of chronic wounds or delayed wound healing.^[^ ^124^ ^]^ Whether stemming from intrinsic factors (e.g., diabetes, hyperglycemia, immunosuppression, autoimmune diseases) or localized stimuli (e.g., infections, hypoxia), this ultimately leads to a sustained inflammatory response, overstimulation of inflammatory vesicle production, and an imbalance in the natural regulation of proinflammatory and anti‐inflammatory processes.^[^ ^56^ ^]^ Elevated and persistent levels of proinflammatory cytokines (e.g., tumor necrosis factor‐alpha [TNF‐α] and interleukins [ILs]), protease activity (e.g., matrix metalloproteinases [MMPs]), and ROS can disrupt, impair, and inhibit the functions of key cell types involved in wound healing, including macrophages, neutrophils, fibroblasts, and endothelial cells.^[^ ^125^, ^126^, ^127^ ^]^ The dysfunction of these key cellular processes further exacerbates inflammation and tissue degradation, while inhibiting beneficial processes critical for wound healing (e.g., growth factor and ECM production).^[^ ^128^ ^]^ This leads to a vicious cycle that continuously hinders wound healing and ultimately results in the formation of a chronic wound.^[^ ^128^ ^]^

This section also addresses the infectious factors that induce a sustained inflammatory response, widely regarded as a common and significant threat to wound healing and skin repair.^[^ ^67^ ^]^ Microbial populations that initially contaminate and colonize injured tissues can disrupt various cell types and interfere with multiple stages of the wound‐healing process.^[^ ^67^, ^129^ ^]^ Antimicrobial treatment of wound infections becomes particularly challenging once biofilms form, as they are typically colonized by *Staphylococcus aureus (S. aureus)* and *Pseudomonas aeruginosa (P. aeruginosa)*.^[^ ^130^ ^]^ Additionally, factors such as neuropathy, hyperglycemia, hypertension, and persistent inflammation can lead to an imbalance in the angiogenic process, which is another critical factor influencing wound healing.^[^ ^131^ ^]^ These conditions often cause disturbances in angiogenesis, resulting in a persistent hypoxic microenvironment that activates oxidative stress in endothelial cells, contributing to increased vasoconstrictor factors, which in turn exacerbate the inflammatory response.^[^ ^132^, ^133^ ^]^ Furthermore, insufficient angiogenesis compromises nutrient supply and metabolic waste exchange, further delaying tissue reconstruction and wound healing.^[^ ^110^ ^]^ As a result, the exploration of efficient anti‐inflammatory, anti‐infective, and angiogenesis‐promoting strategies has become a central focus in tissue engineering research.

## 3 Therapeutic Dressings and Wound Healing Applications

Currently, the primary methods of wound management in clinical practice include debridement, regular dressing changes, antibiotic therapy, and autologous skin grafting.^[^ ^134^ ^]^ Researchers have also developed advanced wound healing strategies, including gene therapy,^[^ ^135^ ^]^ growth factor delivery,^[^ ^136^ ^]^ nanotherapeutic,^[^ ^137^ ^]^ stem cell therapy,^[^ ^138^ ^]^ RNA interference,^[^ ^139^ ^]^ bioengineered skin grafts,^[^ ^140^ ^]^ smart dressings,^[^ ^141^ ^]^ and 3D bioprinting,^[^ ^142^ ^]^ all aimed at restoring the original function of healed skin and regenerating damaged tissue. Despite the development of various emerging therapeutic strategies, limitations such as preparation complexity, high costs, concerns over bioavailability and safety, and uncertain efficacy have hindered their clinical application.^[^ ^111^, ^143^, ^144^ ^]^ Wound management, whether using traditional methods or emerging therapeutic techniques, requires addressing the underlying cause and applying an appropriate dressing or bandage.^[^ ^145^ ^]^

Wound dressings serve as a temporary matrix and play a crucial role in promoting the wound‐healing process.^[^ ^146^ ^]^ Traditional wound dressings, such as sterilized cotton wool, gauze, and bandages, are commonly used to keep wounds dry and provide a physical barrier.^[^ ^147^ ^]^ However, they lack the skin‐like properties necessary to effectively absorb exudate and promote cell proliferation, thereby limiting the rate of wound closure.^[^ ^147^ ^]^ Additionally, these dressings may adhere to the wound after prolonged contact, making removal difficult and potentially causing secondary damage, which further impedes the healing process.^[^ ^148^, ^149^ ^]^ Consequently, increasing research efforts are directed at overcoming the limitations of conventional dressings and mimicking the wound‐healing microenvironment using advanced technologies. Fully replicating the entire microenvironment remains highly challenging, so most tissue engineers focus on structural frameworks critical for healing, such as thin films, foams, sponges, hydrogels, and nanofibrous films.^[^ ^111^, ^141^, ^150^ ^]^

Electrospun nanofiber membranes stand out for their significant potential as wound dressings, owing to their high specific surface area, microporosity, strong drug‐loading capacity, controlled release properties, and ability to mimic the ECM.^[^ ^9^, ^148^, ^151^ ^]^ However, their functional recovery and regenerative efficiencies remain suboptimal, particularly for large acute, infected, and chronic wounds.^[^ ^148^ ^]^ To address these limitations, researchers have introduced biochemical and biophysical cues to develop stimuli‐responsive and functionalized nanofibers, thereby enhancing the wound‐healing effectiveness of nanofiber membranes.^[^ ^148^ ^]^ Indeed, this strategy has been found to compensate for the shortcomings of conventional nanofiber wound dressings, thereby paving the way for functionalized nanofiber scaffolds in wound healing. Inspired by the bioelectric phenomena in the human body, electrospun nanofibrous scaffolds with electroactive characteristics are gaining widespread attention and gradually emerging.

## 4 Biological Effects of EFs and Their Applications in Skin Wound Healing

### 4.1 Bioelectricity

Bioelectricity is an essential component of living organisms, present in all cell types as EFs, ionic currents, redox processes, and potential differences.^[^ ^18^ ^]^ It plays a pivotal role in key biological processes, including embryogenesis, wound healing, tissue repair and remodeling, and overall organismal development.^[^ ^152^ ^]^

**Figure 3:**
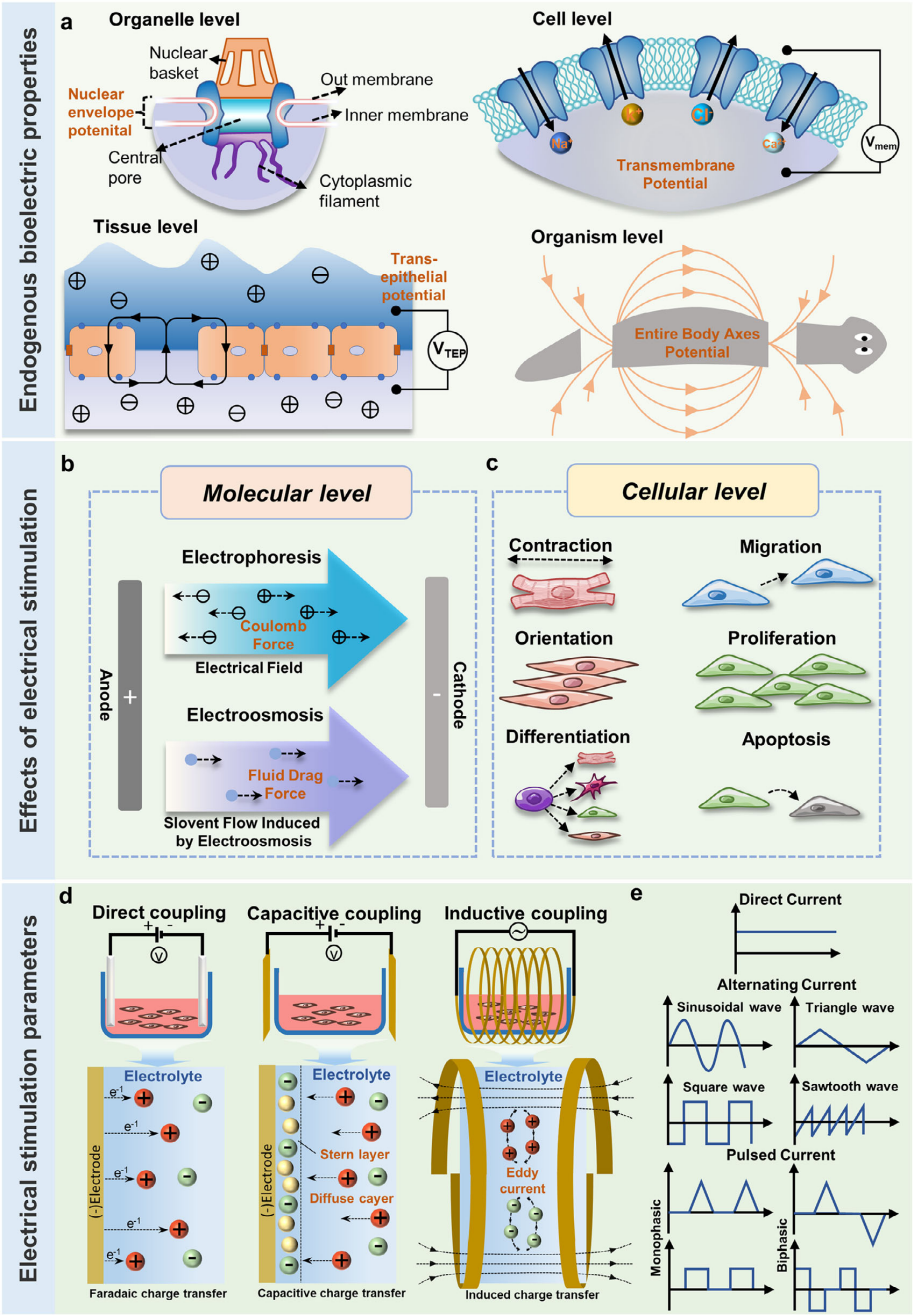
a) Endogenous bioelectric properties at different levels. b) Effects of electrical stimulation on molecular level. c) Effects of electrical stimulation on cellular level. d) Types of exogenous electrical stimulation and possible charge transfer mechanisms. e) Commonly used waveforms for exogenous electrical stimulation.

The phenomenon of bioelectricity was first discovered in brain‐dead frogs by the Italian physician and zoologist Luigi Galvani.^[^ ^153^ ^]^ This discovery marked an important milestone in understanding the complex relationship between electrical phenomena and biological processes. Subsequently, the German physiologist Emil Du Bois‐Reymond (1818‐1896) detailed the electrical activity associated with nerve excitation, muscle contraction, and wounds.^[^ ^154^ ^]^ Building on this foundation, Jaffe, Borgens, and others expanded upon the pioneering work of Matthews (1903),^[^ ^155^ ^]^ demonstrating that the electrical properties of individual cells, epithelia, neural structures, and entire limbs play an instructive role in growth, patterning, and anatomical polarity (**Figure** **3**a).^[^ ^156^, ^157^, ^158^ ^]^ Thus, a strong connection between biology and electricity was established, leading to extensive research in the field. Meanwhile, piezoelectric phenomena in biological tissues were first reported by Martin's team in 1941,^[^ ^159^ ^]^ followed by the measurement of piezoelectricity in dry bone by Fukada and colleagues.^[^ ^160^ ^]^ Continuous research and discovery have revealed piezoelectric effects ranging from microscopic biomolecular components to macroscopic tissues and organs, such as amino acids, peptides, polysaccharides, proteins, bone, ligaments, muscles, and hair.^[^ ^161^ ^]^ Additionally, the complexity of ECM components is linked to their nanocrystalline or liquid crystal‐ordered structure.^[^ ^160^ ^]^

For decades, scientists have explored the concept of EFs in living organisms and the mechanisms underlying their generation. Among these, membrane potential (V_mem_) is regarded as a key indicator in bioelectricity research and can be measured using the second harmonic generation (SHG) technique.^[^ ^162^ ^]^ Membrane potentials are present at the plasma membrane of nearly all mammalian cells, ranging from ≈10 to 90 mV depending on cell type.^[^ ^163^ ^]^ These potentials mediate several key parameters that regulate cellular behaviors, including division, proliferation, oriented polarization, migration, differentiation, and apoptosis.^[^ ^18^, ^163^ ^]^ Whether in electrically excitable cells, such as neurons and myocytes that depolarize to generate action potentials, or in non‐excitable cells, membrane potential has been utilized as a cell‐autonomous bioelectric regulator.^[^ ^163^, ^164^ ^]^ Its key characteristics can be approximated using a simple integrated feedback circuit.^[^ ^165^ ^]^ The effect of V_mem_ on cell behavior is mediated by the activity of transmembrane ion channels (e.g., Nav1.5, Kir2.1, GlyR),^[^ ^166^ ^]^ proton pumps (e.g., V‐ATPase),^[^ ^167^ ^]^ and gap junction complexes (e.g., Connexin43).^[^ ^168^ ^]^ These structures regulate key cellular behaviors by modulating the transmembrane transport of specific ions and molecules.^[^ ^152^ ^]^ Several key events contribute to this process, including the electrophoretic traction of small signaling molecules through intercellular gap junctions, voltage‐dependent regulation of signaling molecule transport (e.g., calcium ions and various neurotransmitters), voltage‐driven conformational changes in integrin proteins, and the electrophoretic segregation or aggregation of protein complex subunits within the cell membrane.^[^ ^169^ ^]^ These effectors subsequently influence nuclear events, including gene transcription, cytoskeletal remodeling, and the physical properties of the cell, leading to the up or down‐regulation of genes that control cell proliferation, migration, and adhesion.^[^ ^152^, ^169^ ^]^ Of particular interest is the interplay between biochemistry, bioelectricity, and biomechanics. Bioelectricity, as a key driver of morphogenesis, does not diminish the crucial roles of biochemical and biomechanical mechanisms.^[^ ^152^ ^]^ Instead, it likely functions as part of a ‘morphogenetic field’, integrating all non‐local signals from cells and cell populations throughout the organism.^[^ ^170^ ^]^ Within the intricate network of multimodal processes that co‐direct morphogenesis, bioelectricity facilitates unique and powerful information processing capabilities at the organ level, enabling cellular assemblies to make coordinated decisions and expand into complex morphogenetic collectives.^[^ ^18^ ^]^ It is important to recognize that bioelectricity is not merely a simple mechanism within the complex molecular cascade of cellular processes. Therefore, rational modulation of exogenous membrane potential presents significant opportunities for therapeutic applications, particularly in regenerative medicine.

### 4.2 Endogenous Electric Field of Skin Wound

Since the 19th century, when German physiologist Emil Du Bois‐Reymond first recorded the natural electric current generated at human skin wounds,^[^ ^171^ ^]^ researchers have proposed the “skin battery” theory, emphasizing that electric field strength is closely related to both the distance from the wound edge and the wound surface area.^[^ ^22^, ^25^, ^171^, ^172^ ^]^

With advancing research, the generation of endogenous EFs in skin wounds has been found to be closely linked to transepithelial potential (TEP).^[^ ^173^ ^]^ The TEP arises from the asymmetric distribution of ion channels, generating transcellular currents that create polarized, ion‐directed transport across epithelial cells.^[^ ^163^, ^172^ ^]^ The epithelial cells of healthy skin maintain a TEP of ≈10 to 60 mV between cells.^[^ ^174^ ^]^ When a wound occurs, the TEP short‐circuits, creating a potential gradient between the wound site and the uninjured area or wound margin, which drives a current to the wound site and generates a lateral electric field with strengths ranging from ≈40 to 200 mV mm^−1^.^[^ ^174^, ^175^ ^]^ A study by Hołyńska‐Iwan et al. reveals that a transient current averaging 4 A cm^−^^2^ is observed at the wound site shortly after injury.^[^ ^176^ ^]^ This current gradually increases to 10 A cm^−2^ within 120 min post‐injury and stabilizes at 4 to 8 A cm^−2^.^[^ ^176^ ^]^ The lateral electric field induces the electrotactic migration of epithelial cells and various other cell types, including neutrophils, lymphocytes, monocytes, macrophages, ECs, and fibroblasts, from the wound edge towards the wound bed.^[^ ^177^, ^178^ ^]^ Electrically driven migration can surpass the effects of chemical gradients and biomechanical forces.^[^ ^177^ ^]^ In this process, the ionic dynamics of Cl⁻ and Na⁺ play a critical role, as their relative concentrations determine the potential difference.^[^ ^179^, ^180^ ^]^ Therefore, Cl⁻ and Na⁺ pumps are essential for maintaining the wound potential. A 2006 study published in *Nature* explored the genetic mechanisms underlying cell electrotaxis and electric field‐induced wound healing, identifying the tumor suppressor phosphatase and tensin homolog (PTEN) as a key regulator of electrotaxis.^[^ ^181^ ^]^ Additionally, signal transduction may occur through the epidermal growth factor receptor (EGFR) pathway (ERK1/2) and the integrin pathway (Rac1).^[^ ^173^ ^]^

Therefore, EFs are one of the many guidance cues in wound healing, playing a critical role in directing cell migration and providing new avenues for wound management through electrical signal transduction.^[^ ^182^, ^183^ ^]^ However, current technology only allows for the measurement of EFs at the wound surface, limiting our understanding of deep dermal EFs and the electrical properties of chronic wound areas.^[^ ^183^ ^]^ This lack of information may hinder the effectiveness of electrical stimulation in treating chronic wounds. Furthermore, other mechanisms beyond PTEN, EGFR, and integrin pathways are likely involved in electrotaxis and electric field‐driven wound healing, suggesting a more complex network of pathways that require further investigation.

### 4.3 Exogenous EFs and Their Effects on Cellular Responses in Wound Healing

As the understanding of bioelectricity in human tissues and the endogenous EFs generated after skin injury has advanced, exogenous EFs have become a focal point of research and a broad range of biomedical effects.^[^ ^184^ ^]^ In tissue engineering and regenerative medicine, exogenous EFs (including self‐powered systems) can facilitate the transport of both charged and uncharged biomolecules through biological membranes via electrophoresis and electroosmosis at the molecular level and promote cellular proliferation, migration, orientation, apoptosis, stem cell differentiation at the cellular level (Figure 3b,c), as well as the remodeling and maturation of engineered tissue constructs.^[^ ^22^, ^185^, ^186^, ^187^, ^188^, ^189^ ^]^

Exogenous electrical stimulation involves the transfer of charges from an external source to the target tissue, generating a transmembrane potential by modulating ion channels, pumps, and gap junction complexes on the cell membrane.^[^ ^189^ ^]^ The four primary methods of delivering exogenous electrical stimulation are direct coupling, capacitive coupling, inductive coupling, and piezoelectric stimulation.^[^ ^161^, ^185^ ^]^ Direct coupling is a straightforward and widely used method in which electrodes are directly inserted into the medium, making contact with the conductive scaffold to deliver electrical stimulation.^[^ ^185^ ^]^ However, this method encounters challenges related to electrode biocompatibility, as electrode‐medium contact can result in temperature increases, pH changes, and the production of harmful by‐products.^[^ ^190^ ^]^ This form of charge transfer, caused by the direct movement of the electrode across the electrolyte interface, is known as Faraday charge transfer and involves the interaction between electrons and ions (Figure 3d).^[^ ^187^, ^191^ ^]^ In contrast, capacitive coupling is a non‐invasive method with enhanced biosafety, involving the placement of two electrodes on opposite sides of the medium to generate a uniform electric field for the cells.^[^ ^185^ ^]^ However, the efficiency of capacitive coupling is relatively low, requiring high voltage between electrodes and prolonged treatment times.^[^ ^192^ ^]^ In this method, charge transfer is governed by the discharge and charging principles of electrical double‐layer capacitors (EDL), involving only the free movement of ions without electron transfer—a process referred to as capacitive charge transfer (Figure 3d).^[^ ^193^ ^]^ Inductive coupling is commonly used to simulate natural charge transfer in the human body, without direct contact with cells. In this approach, a controllable electromagnetic field is generated by conductive coils placed around the cell culture system.^[^ ^185^, ^194^ ^]^ In this method, an electromotive force is induced perpendicular to the axis of the time‐varying magnetic field, driving charge movement from the lower to the higher potential, in accordance with Faraday's law of induction (Figure 3d).^[^ ^195^ ^]^ While this method avoids direct contact, it requires specialized equipment and consumes significant resources, making it less commonly utilized.^[^ ^196^ ^]^ Exogenous electrical stimuli can also be classified into monophasic and biphasic currents based on their directionality (Figure 3e).^[^ ^25^, ^197^ ^]^ Monophasic currents include direct current (DC) and unidirectional pulsed current (PC), both characterized by a constant charge imbalance that mimics endogenous EFs.^[^ ^163^ ^]^ In contrast, biphasic currents consist of opposite polarity (e.g., biphasic pulses, symmetrical waveforms, square waves, or triangular waves) (Figure 3e),^[^ ^198^ ^]^ causing the direction of the current to alternate direction, thus reducing electrochemical effects or thermal effects, making this method highly promising for clinical applications.^[^ ^163^, ^198^, ^199^ ^]^ Notably, regardless of the method employed, electrical stimulation exerts similar effects on cells.^[^ ^200^, ^201^ ^]^

The piezoelectric effect is a linear electromechanical coupling phenomenon that encompasses both direct and inverse piezoelectric effects.^[^ ^202^ ^]^ This phenomenon was first discovered by the Curie brothers in 1880.^[^ ^202^ ^]^ The piezoelectric effect occurs in piezoelectric materials when external mechanical stress is applied, causing the centers of positive and negative charges within the dipoles to shift toward opposite surfaces of the material.^[^ ^202^, ^203^ ^]^ This shift leads to the formation of an internal potential difference (referred to as ‘polarization’) between the surfaces, ultimately generating a transient current.^[^ ^203^ ^]^ Piezoelectric material‐mediated stimuli can be delivered non‐invasively,^[^ ^161^, ^204^ ^]^ and research has explored their applications in nerve regeneration,^[^ ^205^ ^]^ musculoskeletal tissue repair,^[^ ^206^ ^]^ and neovascularization.^[^ ^207^ ^]^ Piezoelectric materials mediate electrical signals through both direct and indirect effects, and their electrical behavior is governed by the piezoelectric effect, which can be described as a relationship between physical quantities such as stress, strain, electric field, and potential shift, according to Hooke's and Maxwell's laws:^[^ ^208^, ^209^ ^]^

(1) $$J_{D}=\frac{\partial D}{\partial t}=\varepsilon_{0}\frac{\partial E}{\partial t}+\frac{\partial P}{\partial t}$$

where *J_D_* is displacement current density, *D* is electric displacement, ε_0_ is the vacuum permittivity, *E* is the electric field, *t* is time, and *P* is the polarization field. With polarization in the z‐axis direction, the displacement current density is given as:

(2) $$J_{D_{z}}=\frac{\partial P_{z}}{\partial t}=\frac{\partial \sigma_{P\left(z\right)}}{\partial t}$$

where $\partial P_{z}$ is piezoelectric charges that accumulate at both ends of the material. The open circuit voltage is expressed as:

(3) $$V_{OC}=k\frac{\sigma_{P\left(z\right)}}{\varepsilon }$$

where *k* is the thickness of piezoelectric media. In the absence of an external electric field and with a membrane of constant thickness, the piezoelectric membrane can be considered an AC voltage source relative to the excitation time, and its output is influenced by the piezoelectric coefficient of the material, as the magnitude of the polarized charge is dependent on this coefficient.^[^ ^208^, ^209^, ^210^ ^]^

This section will focus on the effects of exogenous EFs on the primary cell types involved in skin wound healing, including keratinocytes, fibroblasts, endothelial cells, and macrophages. Notably, there are relatively few direct studies investigating the effects of the piezoelectric effect on cell behavior, with existing research primarily focusing on how the electrical stimulation generated by piezoelectric materials promotes cell migration, proliferation, and differentiation in vitro.^[^ ^211^ ^]^ However, no unified consensus exists regarding the optimal piezoelectric coefficient for different cell behaviors, which remains an important direction for future research. Therefore, this section will primarily discuss the effects of three types of exogenous electrical stimulation on cellular behavior, such as direct coupling, capacitive coupling, and inductive coupling, and the main effects are summarized (**Table** **1**).

**Table 1:** Summarizes the effects of various types of exogenous electrical stimulation on keratinocytes, fibroblasts, endothelial cells, and macrophages.

Cell type	Cell source	Type of current	Type of delivery ES	Electrical parameters	Key effects on cellular behavior	Potential mechanism of action	Refs.
Keratinocytes	Human epidermal keratinocytes derived from neonatal foreskins	DC	Direct coupling	EF: 0–400 mV mm^−1^/Time: 2.5 h	Cells exhibit random migration in fields below ≈10 mV mm^−1^, while directed migration toward the negative pole occurs in fields between 10 and 400 mV mm^−1^.	The electrical properties of cells are primarily governed by their plasma membrane, which demonstrates exceptionally high electrical resistance to current flow.	[212]
	HaCaT cells (Cell Bank of the Chinese Academy of Sciences in Beijing, China)	DC	Direct coupling	EF: 200 mV mm^−1^/Time: 6 h	The range of cell motion increased significantly, and the cells exhibited a distinct tendency to migrate toward the positive pole.	Accelerated microtubule acetylation by regulating Paxillin/HDAC6.	[216]
	Primary keratinocytes from Pten^flox^/^flox^ mice	DC	Direct coupling	EF: 0–200 mV mm^−1^/Time: 2 h	Directed migration	Activated PI(3)Kγ and PTEN signaling pathways.	[181]
	Primary human skin keratinocytes from human skin biopsies	DC	Direct coupling	EF: 50, 100, 150, and 200 mV mm^−1^/Time: 6 and 24 h	Exposure to 100 and 150 mV mm^−1^ for 6 or 24 h significantly increased cell proliferation rates.	Activated the ERK1/2 and p38 MAP kinase pathways, and increased EGF and VEGF secretion.	[215]
	HaCaT cells (Cell Resource Center, Institute of Basic Medical Sciences, Chinese Academy of Medical Sciences)	AC	Capacitive coupling	EF: 58 mV mm^−1^/Time: 2 h per day, 5 days/Frequency: 10, 60, and 110 Hz	ACEF significantly promotes the proliferation of HaCaT cells, with only 60 Hz ACEF further enhancing their migration.	N/C	[217]
	Human epidermal keratinocytes derived from neonatal foreskins	PC	Direct coupling	Voltage: 3 V or 5 V/Time: 5 min per day, 5 days/Frequency: 4,800 Hz	High‐frequency pulsed electric stimulation suppressed the growth of keratinocytes and concomitantly induced keratinocyte differentiation.	Accelerated the L‐type VGCC and/or the extracellular calcium‐sensing receptor	[218]
	Primary keratinocytes were isolated from newborn BALB/C mice	PC	Direct coupling	EF: 150 mV mm^−1^/Time: 3 h/Frequency: 0.1 Hz	After 30 min of stimulation, the cells exhibited clear directional migration.	MAPK/ERK pathway may be critical for PC‐induced keratinocyte electrotaxis.	[219]
	Human epidermal keratinocytes derived from neonatal foreskins	RF	Direct coupling	Current density: 50 or 100 µA mm^−2^/Time:5 min per 4 h, 48 h/ Frequency: 448 kHz	Exposure to 100 µA mm^−^^2^ significantly enhances cell proliferation.	The expression and/or location of E‐cadherin, p‐FAK, MMP‐9, and β‐catenin would exert potential inhibitory effects on cell migration.	[220]
Fibroblasts	HDFs (HDF‐a, 2320, ScienCell, USA)	AC	Capacitive coupling	EF: 58 mV mm^−1^/Time: 2 h per day, 5 days/Frequency: 10, 60, and 110 Hz	ACEF significantly promotes the proliferation of HDF cells, with only 60 Hz ACEF further enhancing their migration.	N/C	[217]
	HDFs isolated from neonatal foreskin	RF	Direct coupling	Current density: 50 or 100 µA mm^−2^/Time: 5 min per 4 h, 48 h/ Frequency: 448 kHz	Exposure to 100 µA mm^−^^2^ significantly enhances cell proliferation.	The expression and/or location of E‐cadherin, p‐FAK, MMP‐9, and β‐catenin would exert potential effects on cell migration.	[220]
	HDFs (Akron General Medical Center)	DC	Direct coupling	EF: 25,50, and 100 mV mm^−1^/Time: 10 min	Exposure to 25–100 mV mm^−1^ shows an increase in random migration behavior, but no directional migration occurs.	The mRNA transcript levels of the four integrins (αV, β3, α5, and β5) showed no significant changes.	[221]
	HDFs cell line (161BR, ECACC No. 90011809; CLFB) and primary HDFs derived from the eyelids of adult donors	DC	Direct coupling	EF: 50, 100, and 400 mV mm^−1^/Time: 5 h	Human dermal fibroblasts exhibit anodal migration, with directional migration becoming more pronounced at higher electric field strengths (400 mV mm^−1^).	The activation of PI3 kinase/Akt signaling mediates electrotaxis.	[222]
	Primary HDFs	AC	Direct coupling	Current: 50 and 100µA/Time: 4 h/Frequency: 0.3 Hz	Low‐intensity DC (100µA) promotes migration to the negative pole.	N/C	[223]
	HDFs from 27‐ and 68‐year‐old human skin (HF27 and HF68, respectively)	PC	Direct coupling	Voltage: 1, 3, and 5 V/Time: 15, 30, and 60 min/Frequency: 4,800 Hz	A voltage of 5 V at a PES exposure time of 60 min significantly increased human dermal fibroblast proliferation	The production of three growth factors—PDGF, FGF2, and TGF‐β1—stimulates the proliferation of HDFs.	[224]
	Primary HDFs derived from a 33‐year‐old woman	PC	Direct coupling	Current: 200 µA/Time: 24 h/Frequency: 2 Hz/Duty cycles: 10%, 50%, and 90%	A duty cycle of 10% promotes cell differentiation, whereas a duty cycle of>50% induces cell death.	The duty cycle of 10% significantly promoted the mRNA expression of α‐SMA and TGF‐β1.	[225]
	27‐year‐old HDFs (HF27)	PC	Direct coupling	Voltage: 5 V/Time: 60 min/Frequency: 4,800 Hz	PES induces fibroblast proliferation and fibroblast‐to‐myofibroblast transition.	The proliferation increase is attributed to PDGFA expression, while differentiation results from the upregulation of α‐SMA expression.	[226]
	Primary HDFs derived from human skin biopsies	PC	Direct coupling	EF: 50 and 100 mV mm^−1^ / Time: 24 h/Duty cycle: 50% and 0.83%	Exposure to 300 s of stimulation demonstrated greater cell migration and fibroblast‐to‐myofibroblast transition.	The enhanced migration was attributed to increased FGF2 secretion, while differentiation was linked to the activation of the Smad signaling pathway.	[227]
	Primary skin fibroblasts were extracted from human skin biopsies.	PC	Direct coupling	EF: 100 mV mm^−1^ /Time: 24 h/Duty cycle: 50% and 0.83%	Accelerated cell migration and promote fibroblast‐to‐myofibroblast transition	Activated TGFβ1‐ERK‐NF‐κB signaling pathway.	[228]
	NIH‐3T3 cells	BES	Direct coupling	Voltage: 5 V/Time: 1 h (2 times per day)/Frequency: 1.688 Hz	Promoted the proliferation of fibroblasts and the production of collagen and growth factors	N/C	[229]
	HDFs from the foreskin of males (aged 2–14 years)	DC	Direct coupling	EF: 50 and 200 mV mm^−1^/Time: 2, 4, and 6 h	Increased proliferation and migration	Induced FGF2 secretion	[230]
Endothelial cells	HUVECs line (ATCC)	DC	Direct coupling	EF: 50–300mV mm^−1^/Time: 24 h	Causing significant cell elongation, orientation, and directional migration.	Activated VEGF receptor, Rho‐ROCK, and PI3K‐Akt signaling pathways.	[231]
	Primary HUVECs (Lonza), Primary HMECs (Lonza)	DC	Direct coupling	EF: 50–300 mV mm^−1^/Time: 3 h	Both cell types exhibited faster migration toward the cathode; HUVECs responded to fields as low as 50 mV mm^−1^, while the threshold for HMECs was 100 mV mm^−1^. Mitosis was stimulated at 50 mV mm^−1^ for HMECs and at 150 mV mm^−1^ for HUVECs, but the cleavage plane became oriented orthogonal to the field vector at 200 mV mm^−1^ in both cell types.	Upregulated expression of CXCR4 and CXCR2 chemokine receptors and phosphorylation of both chemokines.	[232]
	HUVECs (ATCC)	DC	Direct coupling	EF: 50 and 200 mV mm^−1^/Time: 2 and 6 h	Increased proliferation, proliferation rate, migration, invasion, angiogenesis	Induced FGF2 secretion and activated MAPK/ERK signaling pathway.	[230]
	HUVEC line (ATCC)	DC	Direct coupling	EF: 50–200 mV mm^−1^/Time: 4, 6, and 8 h	Stimulated the neovessel formation of ECs in 3D culture, especially 150 mV mm^−1^	Activated VEGFR‐2‐mediated signaling pathway.	[233]
	HMEC‐1s (Dr. F.J. Candal, Centers for Disease Control, Bethesda, Md), HUVEC (ATCC)	DC	Direct coupling	EF: 50–400 mV mm^−1^/Time: 0, 4, 6, 8, and 24 h	Induced directed migration, and reorientation. HMEC‐1s migrated toward the cathode, whereas the HUVEC cell migrated toward the anode.	N/C	[234]
	HUVECs were isolated from human umbilical cord veins	ELF‐EMFs	Inductive coupling	Magnetic field: 1 mT/Time:12 h/Frequency: 50 Hz	Increased proliferation and tubule formation.	Activated VEGF signal pathway.	[235]
	HUVEC lines and mouse microvascular lines (Lonza)	LF‐ EMF	Inductive coupling	Magnetic field: 4 mT/Time:340 µs/Frequency: 72 Hz	Promoted the proliferation of both types of endothelial cells.	N/C	[236]
	HUVECs (Lonza)	PC	Direct coupling	EF: 50, 155, and 360 mV mm^−1^/Time: 48 h/Duty cycles of 14.3% and 50%/Frequency: 0.0083 Hz	Initiation, growth, and switching of endothelial sprouts to anodic polarization.	N/C	[237]
	Primary HUVECs (neonatal pooled, 200P–05N, Sigma‐Aldrich)	PC	Direct coupling	Voltage: 1.2 V/Time: continuous/Frequency: 1.2 Hz	Stimulation initially suppressed growth during the first 24 h but ultimately led to adaptive proliferation.	N/C	[238]
	EA.Hy926	DC	Direct coupling	EF: 50–250 mV mm^−1^ /Time: 10–24 h	Obvious cathodic directional migration with voltage dependence	N/C	[239]
	HUVECs lines (ATCC)	DC	Direct coupling	EF: 50, 100, and 200 mV mm^−1^/Time: 72 h	Cell density, cell growth rate, and mitotic index decreased significantly upon exposure to 200 mV mm^−1^.	The downregulation of cyclin E expression and upregulation of p27kip1 induce G1 phase cell cycle arrest, thereby controlling cellular proliferation.	[240]
Macrophages	Human monocyte‐derived macrophages were isolated from the blood of healthy adult‐consenting donors	DC	Direct coupling	EF: 5–450 mV mm^−1^/Time: 2 h	Anode‐directed cell migration and modulate macrophage phagocytosis and cytokine secretion.	Induced mobilization of intracellular Ca^2+^, activation of PI3K‐Akt signaling pathways, and reorganization of the actin cytoskeleton.	[241]
	THP‐1 cells (TIB‐202, ECACC)	DC	Direct coupling	EF: 100 mV mm^−1^/Time: 1 h per day for 3 days	Modulates the polarization of THP‐1‐derived macrophages towards an M2 phenotype in both M0 and M1 cells.	Regulated PPARG signaling	[242]
	THP‐1 cells (Beyotime Company)	DC	Direct coupling	EF: 15 V cm^−1^/Time: 12 h	Induced macrophage polarization toward the M2 type.	Inhibited actin polymerization in macrophages and the downstream PI3K signaling pathway.	[243]
	THP‐1 ​cells (TCHu 57, Cell Bank of Chinese Academy of Sciences)	AC	Capacitive coupling	EF:58 mV mm^−1^/Time:2 h per day, 5 days/Frequency: 10, 60, and 110 ​Hz	Facilitated the M2‐type polarization of macrophages.	Promoted YAP/TAZ expression of macrophages.	[217]
	mouse bone marrow‐derived macrophages (BMDMs) from C57BL/6 mice	AC	Direct coupling	Voltage: 1–1000 mV/Time: 6 h per day/Frequency: 1, 100, 500, and 1000 Hz	The square waveform only promotes M1 polarization. The sine waveform can promote both M1 and M2 polarizations.	The square waveform affected the intracellular ion concentration and the sinusoidal waveform promoted both the intracellular ion concentration and membrane receptors.	[244]

Keratinocytes, residing in the outermost layer of the skin, play a vital role in wound healing, with their repair speed closely associated with the overall healing rate.^[^ ^22^ ^]^ Studies have shown that human keratinocytes are highly sensitive to direct current EFs ranging from 10 to 400 mV mm^−1^.^[^ ^212^ ^]^ In fields of 5 mV mm^−1^ or lower, keratinocytes exhibit random migration;^[^ ^213^ ^]^ however, as field strength increases (10 to 400 mV mm^−1^), they migrate directionally towards the cathode.^[^ ^212^ ^]^ Conversely, Liu and colleagues found that EFs at 200 mV mm^−1^ induced the migration of human immortalized epidermal cells (HaCaT) towards the anode while upregulating Paxillin, downregulating HDAC6, and promoting microtubule acetylation.^[^ ^214^ ^]^ When exposed to DC electrical stimulation at 100 to 150 mV mm^−1^ for 24 h, keratinocyte proliferation significantly increased, while no significant effect was observed at 200 mV mm^−1^.^[^ ^215^ ^]^

In addition to DC fields, alternating current (AC) fields, pulsed electrical stimulation, and radiofrequency currents also affect keratinocytes. For example, AC EFs at 10, 60, and 110 Hz (58 mV mm^−1^) significantly promoted HaCaT keratinocyte proliferation, with 60 Hz also enhancing cell migration—a response similarly observed in human dermal fibroblasts and macrophages.^[^ ^217^ ^]^ Pulsed electrical stimulation can also induce electrotaxis in keratinocytes, similar to the effects observed with direct current EFs (DCEFs). Ren and colleagues found that after 30 min of stimulation at 150 mV mm^−1^, with a 60% duty cycle and a frequency of 0.1 Hz, keratinocytes exhibited directional migration, with their positive electrotaxis persisting for 30 to 90 min after the field was turned off.^[^ ^219^ ^]^ This study also revealed, for the first time, that the ERK1/2 signaling pathway is involved in the electrotactic mechanism under pulsed DCEF.^[^ ^219^ ^]^ Additionally, Arai's team found that when cultured keratinocytes were exposed to an electric field at 4800 Hz and 3 V (≈100 mV mm^−1^) or 5 V (≈166 mV mm^−1^) for 5 min per day over 5 days, cell growth and differentiation were significantly inhibited under 5 V stimulation compared to the control group.^[^ ^218^ ^]^ The underlying mechanism may involve the accelerated activation of L‐type voltage‐gated calcium channels (VGCC) and/or extracellular calcium‐sensing receptors.^[^ ^218^ ^]^ The two available studies indicate that the effect of pulsed electrical stimulation on keratinocytes is primarily dependent on voltage and duty cycle, with frequency playing a role only at very high levels. Radiofrequency (RF) currents (100 µA mm^−^^2^ intermittent exposure for 48 h) promoted the proliferation and migration of human keratinocytes and fibroblasts, with fibroblasts exhibiting a faster migration rate.^[^ ^220^, ^245^ ^]^ Considering that keratinocytes in vivo are organized into tightly packed layers, whether collective cell migration exhibits electrotaxis remains to be explored.^[^ ^246^, ^247^ ^]^ A preliminary study using a microfluidic electrical stimulation platform found that a unidirectional electric field (200 mV mm^−1^) had a more pronounced effect on the healing dynamics of collective keratinocyte migration.^[^ ^248^ ^]^

Fibroblasts play a critical role in granulation tissue formation.^[^ ^22^ ^]^ Initial findings suggest that human dermal fibroblasts, in both primary and cell line cultures, do not exhibit directional migration over short periods (10 min), but may display slow, directed migration toward the anode over longer periods (generally greater than 1 h) in DC fields of 25–100 mV mm^−1^.^[^ ^221^ ^]^ This behavior may be related to differences in the efficiency of the keratinocyte response. Thus, higher field strengths (greater than 200 mV mm^−1^) may be necessary to induce directional migration within 1 h of field initiation.^[^ ^222^ ^]^ Furthermore, microcurrent stimulation at 100 µA, DC with a frequency of 0.3 Hz for 4 h resulted in significantly greater mobility toward the negative pole compared to 50 µA and 0 µA.^[^ ^223^ ^]^ Subsequently, numerous studies have demonstrated that pulsed electric stimulation (PES) with varying parameters can induce fibroblast proliferation, differentiation, and migration.^[^ ^224^, ^225^, ^226^, ^227^, ^228^ ^]^ Notably, PES seems to be more effective in promoting the phenotypic transition of fibroblasts into myofibroblasts.^[^ ^225^, ^227^, ^228^ ^]^ Meanwhile, in an alternating electric field (ACEF) of 58 mV mm^−1^, the optimal effect on human dermal fibroblast proliferation and migration was observed, particularly at 60 Hz.^[^ ^217^ ^]^ Recently, So et al. introduced the concept of bionic electrical stimulation (BES), demonstrating that it had a more pronounced effect on fibroblast proliferation compared to DC and AC.^[^ ^229^ ^]^ While these studies consistently demonstrate the effects of exogenous electrical stimulation on healthy fibroblasts, limited research has focused on abnormal fibroblasts. Current studies suggest that low‐intensity DC electrical stimulation (20 and 40 mV mm^−1^) may promote the proliferation and migration of fibroblasts from diabetic donors.^[^ ^249^, ^250^ ^]^

Angiogenesis is a critical process during the proliferative phase and a key component in the formation of granulation tissue.^[^ ^22^ ^]^ The primary driving force behind angiogenesis is the alignment of ECs into tip and stalk cells.^[^ ^110^ ^]^ Growth, migration, polarization, sprouting, and lumen formation of ECs contribute to the development of a functional circulatory system.^[^ ^251^ ^]^ Studies indicate that DC EFs ranging from 75 to 400 mV mm^−1^ induce physical changes in endothelial cells, such as realignment of the cell's long axis, elongation, proliferation, changes in cell shape, and directional migration, all contributing to angiogenesis.^[^ ^230^, ^231^, ^232^, ^233^, ^234^ ^]^ Similar effects have been observed with other forms of electrical stimulation. For instance, extremely low‐frequency electromagnetic fields (ELF‐EMF) enhance endothelial cell proliferation and tubular formation,^[^ ^235^, ^236^ ^]^ while pulsed EFs promote endothelial cell sprouting, growth, and polarization toward the anode.^[^ ^237^ ^]^ Additionally, Abasi and colleagues developed the Electrical Cell Stimulation and Recording Apparatus (ECSARA) to electrically stimulate and monitor cell cultures.^[^ ^238^ ^]^ They observed that human umbilical vein endothelial cells (HUVECs) exposed to AC stimulation (162 mV mm^−1^, 1.2 Hz) exhibited faster growth and formed tight junctions.^[^ ^238^ ^]^ Compared to previous studies in 2D cultures, this study effectively demonstrated the regulatory effects of electrical stimulation on HUVECs in 3D cultures. However, Long et al. and Wang et al. reported opposite results, showing that electric field (EF) strengths of 50 to 250 mV mm^−1^ had no effect on ECs proliferation, while at 200 mV mm^−1^, ECs proliferation was inhibited due to cell cycle arrest or G1 phase blockade.^[^ ^239^, ^240^ ^]^ Therefore, the effects of electrical stimulation on ECs are closely linked to the type of ECs and the specific stimulation parameters, warranting further investigation.

Accelerated recruitment of immune cells and cytokines plays a crucial role in wound healing, with macrophages being central to this process.^[^ ^22^ ^]^ Studies have shown that macrophages exhibit electrotaxis and undergo phenotype transitions under electrical stimulation. Hoare et al. reported that macrophages migrate toward the anode under low electrical stimulation (5 mV mm^−1^), with the migration speed proportional to the electric field strength, peaking around 300 mV mm^−1^.^[^ ^241^ ^]^ Furthermore, DC stimulation at 100 mV mm^−1^ induces polarization of THP‐1‐derived macrophages toward the pro‐regenerative M2 phenotype,^[^ ^242^ ^]^ a response also observed at 150 mV mm^−1^.^[^ ^243^ ^]^ In addition to the effects of DC stimulation, ACEF has also been reported to promote M2 polarization, with 10 and 60 Hz ACEF stimulation enhancing YAP/TAZ expression in macrophages.^[^ ^217^ ^]^ Various waveforms have also been reported to influence macrophage polarization.^[^ ^188^, ^244^ ^]^ Square‐wave stimulation selectively promotes lipopolysaccharide (LPS) and IFN‐γ ‐induced M1 polarization, while sine‐wave stimulation promotes both LPS/IFN‐γ‐induced M1 and IL‐4‐induced M2 polarization.^[^ ^244^ ^]^ Other research groups have attempted to induce macrophage differentiation using wireless electrical stimulation via piezoelectric materials to improve biocompatibility, but the results have been inconsistent.^[^ ^252^, ^253^ ^]^ For instance, ultrasound‐driven piezoelectric stimulation increased the proportion of CD68^+^CD206^+^ M2 macrophages,^[^ ^252^ ^]^ while Kong and colleagues reported enhanced M1 macrophage activity and inhibited M2 polarization.^[^ ^253^ ^]^ These findings indicate that the effects of piezoelectric stimulation on macrophages are still unclear and may vary depending on the specific piezoelectric materials used.

Exogenous electrical stimulation interacts with multiple cell types, influencing behaviors such as contraction, migration, orientation, and proliferation.^[^ ^184^ ^]^ However, the underlying mechanisms remain under investigation and are not yet fully understood. Nonetheless, researchers have proposed several potential physical and biological mechanisms to explain these effects, including changes in aqueous interfaces,^[^ ^254^, ^255^, ^256^ ^]^ electro‐osmosis,^[^ ^257^ ^]^ alterations in membrane potential (asymmetric ion flow and the opening of voltage‐gated channels),^[^ ^246^, ^258^, ^259^ ^]^ and mechanosensation.^[^ ^260^, ^261^ ^]^ Building on these physical mechanisms, external electric field signals are transmitted to cells via receptor‐mediated signaling, triggering a series of biological changes both intracellularly and extracellularly. These changes include the redistribution of membrane components and lipid rafts,^[^ ^262^ ^]^ activation of membrane‐bound receptors,^[^ ^263^, ^264^ ^]^ and initiation of signaling pathways such as Rho/Rac, PI3K/AKT, MAPK/ERK, and TGFβ1/ERK/NF‐κB, as well as increased expression of Ki67, VEGF, and FGF‐2.^[^ ^228^, ^230^, ^231^, ^265^ ^]^ These possible mechanisms are considered to be key factors driving cellular responses, but these mechanisms still need further verification and in‐depth research. In summary, cells involved in skin wound healing can sense external EFs of physiological intensity, regulating their functions and behaviors by altering cell morphology and polarity, thus influencing the skin wound healing process.

Given the responsiveness of key cell types to electrical stimulation during skin wound healing, electrical stimulation has emerged as an innovative tool in skin tissue engineering and has found widespread application.^[^ ^22^, ^28^, ^249^, ^266^ ^]^ Concurrently, with the ongoing development of electroactive biomaterials, they have been recognized as a new generation of smart biomaterials. These materials can directly deliver electrical signals to target cells and tissues or self‐regulate under electrical stimulation to adapt to the cellular microenvironment, thereby further promoting wound healing and attracting increasing attention. A detailed introduction is provided in Section 4.2 of Section 4.

### 4.4 Clinical Application of Electrical Stimulation to Accelerate Wound Healing

Exogenous electrical stimulation has been shown to promote skin healing in vitro cell studies,^[^ ^249^ ^]^ in vivo animal models,^[^ ^267^ ^]^ and clinical research.^[^ ^268^ ^]^ However, from an evidence‐based medicine perspective, the level of evidence provided by in vitro and animal studies is relatively low,^[^ ^269^ ^]^ as these studies primarily demonstrate phenomena and speculate on potential mechanisms of action.^[^ ^270^ ^]^ Therefore, we will focus on systematically summarizing the clinical research on electrical stimulation for accelerating wound healing, to provide stronger evidence and verify its effectiveness in clinical applications.

Over the past 30 years, numerous clinical trials and meta‐analyses have preliminarily demonstrated that electrical stimulation is a safe and effective adjuvant therapy for chronic wounds, including pressure ulcers,^[^ ^268^, ^271^, ^272^ ^]^ diabetic wounds,^[^ ^273^ ^]^ and venous ulcers.^[^ ^274^ ^]^ For example, Avendaño‐Coy et al. conducted a meta‐analysis of randomized controlled trials (RCTs) investigating the effects of microcurrent on wound healing and pain relief in patients with acute or chronic wounds, concluding that it is an effective and safe treatment for reducing wound area, shortening healing time, and alleviating pain.^[^ ^275^ ^]^ Khouri et al. conducted a meta‐analysis of various ES modalities for chronic wounds and found that, when comparing DC, low‐voltage pulsed current (LVPC), high‐voltage pulsed current (HVPC), and diathermy (DW), HVPC demonstrated the greatest improvement in reducing chronic wound size.^[^ ^276^ ^]^

The types and parameters of electrical stimulation used in clinical applications are diverse, such as DC, pulsed DC, and AC. However, the lack of standardization in stimulation parameters and duration, even within the same type of electrical stimulation, poses challenges for standardizing this therapy. In this section, we summarize and discuss clinical trials conducted over the past five years on electrical stimulation for wound healing. A study by Juan and colleagues found that patients who received 10 h of microcurrent therapy (4.2 µA cm^−2^) per day showed a 16.8% greater improvement in overall pressure ulcer healing and a 20.1% greater reduction in wound area compared to the control group.^[^ ^277^ ^]^ Similarly, microcurrent therapy (100 µA, 9 kHz AC, 120 seconds) was shown to accelerate palatal wound healing and reduce discomfort in patients after connective tissue graft surgery.^[^ ^278^ ^]^ Zulbaran‐Rojas et al. used a commercially available bioelectric stimulation technology platform (BEST®), the Tennant Biomodulator® microcurrent device, which delivers high‐voltage pulsed alternating current (HVPAC).^[^ ^279^ ^]^ The waveform they used was an asymmetrical damped sine wave, with the voltage set between 150 and 250 V. This intensity level is FDA‐approved, does not cause harm to patients, and is widely used for transcutaneous electrical nerve stimulation (TENS) for pain relief. The results of the study showed that the BEST® microcurrent platform can effectively accelerate wound healing and improve skin perfusion pressure and tissue oxygen saturation in patients with chronic diabetic foot ulcers (DFU) and mild to severe peripheral arterial disease (PAD).^[^ ^279^, ^280^ ^]^ Additionally, radioelectric asymmetric conveyer (REAC) technology has been applied in the treatment of venous ulcers, where REAC therapy, combined with standard dressings and elastic compression therapy, achieved optimal outcomes in promoting ulcer healing, reducing pain perception, and improving patients' quality of life.^[^ ^274^ ^]^ Remarkably, Hearne and colleagues introduced a new concept: the combined use of ultrasound and electrical stimulation for the treatment of DFU.^[^ ^281^ ^]^ In this approach, electrical stimulation parameters were set at 4000–4250 Hz ±1 in a 4‐pole interferential mode. This combined technique is considered an effective adjunct therapy for hard‐to‐heal diabetic foot ulcers. In addition to the healing rate and wound area as the primary observation indicators, the effects on immune markers were also reported. Anna Polak's research team found that anodal HVPC significantly increased the IL‐10/TNF‐α ratio in serum, suggesting its potential to regulate immune responses and inhibit inflammatory processes, thereby accelerating the healing of chronic wounds.^[^ ^282^ ^]^ Based on existing clinical research, high‐voltage pulsed current has demonstrated the most effective reduction in chronic wound areas.^[^ ^276^ ^]^ This may be attributed to HVPC's ability to avoid noticeable skin changes while more accurately mimicking physiological currents and penetrating deeper into skin tissues to exert its effects.^[^ ^283^ ^]^ Meanwhile, the concept of ‘effective electrical stimulation’ may also play a role. Based on the work of Yuk's research group,^[^ ^284^ ^]^ Kim et al. summarized the requirements for efficient bioelectronic stimulation at the tissue‐electrode interface and proposed ideal electrode characteristics, which include three key aspects: (i) electrical properties, (ii) mechanical properties, and (iii) stimulation pulse type.^[^ ^187^ ^]^ To expand on this, higher signal frequency, lower mechanical modulus of the electrode material, and an optimal pulse phase are key factors in achieving effective electrical stimulation. This work suggests that researchers should consider electrode impedance, the distance from the target tissue, and signal frequency when developing electroactive nanofiber scaffolds to optimize electrical stimulation effects.

Based on the reviewed literature, the number of systematic reviews and meta‐analyses has increased over the past five years, while newly conducted clinical studies remain relatively scarce. This scarcity may be attributed to the significant variability in polarity, duration, and specific methods of electrical stimulation across different studies, making it difficult to establish a standardized application method.^[^ ^283^ ^]^ This variability has hindered the adoption of electrical stimulation as a standardized treatment in wound healing. Currently, physicians in Australia, New Zealand, and the United States recommend ES as an adjunct therapy for treating chronic wounds.^[^ ^285^, ^286^ ^]^ In contrast, the NICE guidelines do not recommend ES therapy for chronic wounds, instead advocating for appropriate wound dressing techniques.^[^ ^287^ ^]^ This highlights the potential and value of electroactive functional wound dressings in wound healing. Section 5 will present a detailed discussion of the advancements in the application of EEN scaffolds (functional wound dressings) in wound healing.^[^ ^288^ ^]^

## 5 Design and Fabrication of EEN Scaffolds for Biomedical Applications

### 5.1 Electrospinning Technology and Principles

The phenomenon of liquids being attracted by electrostatic forces was first observed by William Gilbert in 1600.^[^ ^289^ ^]^ After centuries of development, Boys discovered that fibers could be drawn from viscoelastic liquids under the influence of an external electric field in the 1880s.^[^ ^290^ ^]^ This discovery laid the foundation for modern electrospinning technology. By the early 20th century, Morton and Cooley's team introduced the prototype of an electrospinning device and successfully filed two patents related to electrospinning, opening a new chapter in the technological advancement of this field.^[^ ^290^ ^]^ In the late 1960s, Sir Geoffrey Ingram Taylor used mathematical simulations to study the shape of droplets formed under the influence of an electric field and proposed the renowned ‘Taylor cone’ theory, which laid a crucial theoretical foundation for electrospinning.^[^ ^291^ ^]^ In the early 1990s, Reneker's research group, along with other teams, discovered that many organic polymers could be processed into nanofibers through electrospinning.^[^ ^292^ ^]^ This discovery popularized the term ‘electrospinning’ and infused the technology with new vitality and potential. Since then, research in electrospinning has rapidly expanded, exhibiting exponential growth, and has found widespread applications in fields such as biomedicine, energy, and environmental science.^[^ ^293^ ^]^

Electrospinning is a simple and versatile method for producing nano‐ and microscale fibers.^[^ ^293^ ^]^ By adjusting process parameters (e.g., voltage, flow rate, spinneret design, and collector‐to‐nozzle distance), polymer solution parameters (e.g., molecular weight, viscosity, surface tension, concentration, and solvent selection), and environmental factors (e.g., temperature and humidity), a variety of materials can be used to manufacture fiber/nanofiber structures with different functions and properties for applications such as drug delivery, tissue engineering, and cancer treatment.^[^ ^294^, ^295^, ^296^, ^297^ ^]^ Additionally, depending on specific application requirements and the design of the electrospinning needle (e.g., single‐axial, coaxial, or multi‐axial), various fiber structures can be produced, such as core‐shell, hollow, hybrid, porous, and multilayer fibers.^[^ ^293^ ^]^ Each fiber structure can be engineered for specific functionalities through tailored design and process control, meeting the demands of various fields.^[^ ^298^ ^]^ Compared to other non‐electrospinning techniques (e.g., interfacial polymerization, drawing, self‐assembly, freeze‐drying, phase separation, template synthesis), electrospinning offers distinct advantages, including the production of continuous, highly ordered, and smooth‐surfaced micro‐ and nanofibers.^[^ ^293^, ^299^ ^]^ It remains one of the simplest and most scalable methods for fabricating micro‐ and nanofibers.^[^ ^299^ ^]^

Polymers used in the preparation of fiber scaffolds through electrospinning can be classified into three categories: natural polymers, synthetic polymers, and composite materials.^[^ ^295^ ^]^ Natural polymers mainly consist of polysaccharides and polypeptides, including chitosan (CS), bacterial cellulose (BC), sodium alginate (SA), gelatin (GEL), collagen (COL), and silk fibroin (SF). These materials are highly biocompatible and biodegradable, making them ideal for applications in tissue engineering and regenerative medicine.^[^ ^297^ ^]^ Synthetic polymers, including polycaprolactone (PCL), polyglycolic acid (PGA), polylactic acid (PLA), and poly(lactic‐co‐glycolic acid) (PLGA), provide excellent mechanical properties and controllable degradation rates, making them widely used in biomedical applications.^[^ ^293^ ^]^ Given the advantages and limitations of natural polymers (e.g., excellent biocompatibility but poor mechanical properties and spinnability) and synthetic polymers (e.g., superior mechanical, thermal, and spinnability properties but poor hydrophilicity and biocompatibility), combining them into composite materials can effectively address the limitations of each while maximizing their strengths, making them more suitable for tissue engineering applications.^[^ ^30^, ^39^, ^293^ ^]^ Common methods for preparing composite materials include direct dispersion, gas‐solid reactions, sol‐gel methods, co‐evaporation, and coaxial electrospinning.^[^ ^293^, ^300^ ^]^ These techniques allow for material integration at the molecular or nanoscale, imparting composites with favorable physical properties, structural stability, and bioactivity.^[^ ^293^ ^]^ The successful preparation of polymer solutions is critical in determining the structure and morphology of electrospun nanofibers, typically requiring specific considerations regarding polymer molecular weight and solvent selection.^[^ ^300^ ^]^ Evaluating polymer chain entanglement serves as an effective criterion for assessing fiber‐forming ability during electrospinning.^[^ ^301^ ^]^ A decrease in polymer molecular weight often leads to bead formation instead of fiber formation.^[^ ^302^ ^]^ A homogeneous polymer solution is another key factor, with solvent selection being critical—if the solvent volatility is too high or too low, it will affect the jet's solidification rate.^[^ ^303^ ^]^ Water is generally avoided as a solvent, with common alternatives including methanol, dichloromethane, chloroform, dimethylformamide (DMF), tetrahydrofuran (THF), acetone, dimethyl sulfoxide (DMSO), hexafluoroisopropanol (HFIP), and trifluoroethanol.^[^ ^293^, ^300^ ^]^ In response to these factors, researchers have proposed a formula linking the critical concentration required for electrospinning to polymer molecular weight and effective solvent volume.^[^ ^302^, ^304^ ^]^

(4) $$C_{c}^{*}=\frac{3M_{w}}{4\pi R_{g}^{3}N_{A}}$$

where *N_A_* is the Avogadro constant, $R_{g}$ is the mean radius of gyration, $M_{w}$ is the molecular weight of the polymer. If the polymer concentration is below the critical point (C < $C_{c}^{*}$) inadequate chain entanglement can result in an unstable jet due to Rayleigh instabilities. Therefore, for stable electrospinning, the polymer concentration needs to be higher than the critical point (C > $C_{c}^{*}$). Additionally, process parameters such as voltage, flow rate, and spinneret‐to‐collector distance have also a decisive impact on fiber formation and quality.^[^ ^300^, ^305^ ^]^ The interaction between these parameters can be explained through electrohydrodynamic theory.^[^ ^306^ ^]^

The electrospinning process consists of four steps: i) the formation of a conical jet from a liquid droplet (Taylor cone); ii) the stretching of the charged jet in a single direction; iii) the narrowing of the jet under the applied electric field, leading to whipping instability (electro‐bending instability); and iv) the collection of the jet on a grounded collector surface, where it solidifies into fibers.^[^ ^293^ ^]^ The formation of the Taylor cone is governed by the balance between electrostatic forces and surface tension.^[^ ^307^ ^]^ As the strength of the electrostatic field increases, the accumulation of surface charges overcomes the droplet's surface tension, causing it to stretch, narrow, and form a jet, which undergoes intense whipping and splitting motions.^[^ ^307^ ^]^ If the additional effects of viscosity and ohmic conductivity are neglected, the entire process can be characterized by the electrostatic Bond number and the Weber number.^[^ ^307^, ^308^ ^]^ The relationship between electrostatic forces and surface tension is given as the electrostatic Bond number (also known as the Taylor number):

(5) $$Bo_{e}=\frac{F_{e}}{F_{\gamma }}=\frac{\varepsilon \varphi^{2}}{\gamma D_{O}}$$

and the relationship between inertial forces and surface tension is expressed as the Weber number:

(6) $$We_{D}=\frac{F_{Q}}{F_{\gamma }}=\frac{\rho Q^{2}}{\gamma D_{O}D_{1}^{2}}$$

where *F_e_* is the electrostatic forces, *F*_γ_ is the surface tension, *F_Q_* is the inertial forces, γ is the surface tension coefficient, ε is the dielectric constant, φ is the potential or voltage, *D_O_* is the outer diameter of the capillary, *D*_1_ is the inner diameter of the capillary, ρ is the density, and *Q* is the flow rate. When *Bo_e_* >1, electrostatic forces dominate, leading to the deformation of the droplet exiting the capillary needle into a Taylor cone, which emits a fine jet. When both *Bo_e_* >1 and *We_D_* >1, whipping mode jet motion is observed. Additionally, according to Taylor's 1966 theory,^[^ ^309^ ^]^ the critical voltage required to reach the state at which a jet is emitted under the influence of electrostatic forces is expressed as:^[^ ^310^ ^]^

(7) $$V_{c}^{2}=\frac{4H^{2}}{L^{2}}\left(\ln\frac{2h}{R}-\frac{3}{2}\right)\left(1.3\pi \gamma R\right)\left(0.09\right)$$

where *H* is the distance from the spinneret tip to the collector, *h* is the length of the liquid column, *R* is the inner radius of the spinneret, and γ is the surface tension of the spinning solution. The factor 0.09 is inserted to predict the voltage. It is important to note that during electrospinning, the solution flow rate is low and the capillary needle radius is small, allowing the injection pressure and droplet gravity to be ignored. The conical jet initially flows in a straight line, known as the near‐field region.^[^ ^309^ ^]^ In this region, the viscoelasticity of the fluid must suppress Rayleigh instability, as failure to do so may result in the jet breaking into droplets, a phenomenon known as electrostatic atomization.^[^ ^311^ ^]^ Preparation based on electrostatic atomization technology, when combined with other processes, is also applied in tissue engineering.^[^ ^312^, ^313^ ^]^ Therefore, it is important to distinguish between these methods. Moreover, He et al. investigated the critical radius and length of the electrospinning straight jet.^[^ ^314^ ^]^ By applying the mass conservation law, charge conservation law, momentum conservation law, and Cauchy inequality, the critical length and radius are given as:

(8) $$R_{0}={\left(\frac{2\sigma Q}{\pi k\rho E}\right)}^{1/3}$$

(9) $$L=\frac{KQ^{3}}{\pi \rho^{2}I^{2}}\left(R_{0}^{-2}-r_{0}^{-2}\right)$$

where *Q* is the flow rate, σ is the surface charge, *k* is the dimensionless conductivity, *E* is the applied electric field, *I* is the current passing through the jet, ρ is the liquid density, and *r*_0_ is the initial radius of the jet. Finally, as the diameter of the coils increases, the process involves the bending instability of the charged liquid jet,^[^ ^315^ ^]^ which is considered key to forming nanofibers through electrospinning.^[^ ^316^ ^]^ Subsequently, Fridrikh et al. proposed a model of charged liquid exhibiting bending instability to predict the terminal jet fiber diameter.^[^ ^317^ ^]^ The corresponding prediction formula is as follows:

(10) $$d_{t}={\left[\left(\gamma \tilde{\varepsilon }\left(Q^{2}/I^{2}\right)\left(2/\pi \right)\left(2\ln\tau -3\right)\right)\right]}^{1/3}$$

The equation relates the terminal jet fiber diameter (*d_t_*) to the flow rate (*Q*), current (*I*), dielectric constant of the surrounding medium ($\tilde{\varepsilon }$), and dimensionless wavelength of the bending instability ($\tilde{\varepsilon }\approx l/d$), where *l* corresponds to the radius of the bending disturbance.^[^ ^316^ ^]^ Environmental factors, such as temperature and humidity, also influence the morphology of electrospun fibers, primarily by affecting the solvent evaporation rate.^[^ ^316^ ^]^ For example, Ghobeira et al. used a mixed solvent of acetic acid and formic acid for PCL electrospinning and found that at 25°C, the average fiber diameter increased by 237% as the relative humidity rose from 35% to 65%.^[^ ^318^ ^]^

In summary, electrospinning is a simple, versatile, and theoretically well‐established method for generating nanofibers. The formation of electrospun nanofibers is influenced by various parameters, such as voltage, liquid feed rate, spinneret design, collector distance, and environmental conditions.^[^ ^293^ ^]^ By adjusting these parameters, nanofibers with diverse structures can be produced. High‐quality electrospinning is typically defined by the ability to consistently eject viscoelastic liquid to form continuous fibers under an applied electrostatic field, resulting in uniform fiber diameters and minimal bead defects.^[^ ^316^ ^]^

### 5.2 Electroactive Biomaterials of EEN Scaffolds

In tissue engineering, scaffolds are commonly employed to replicate key components of the natural ECM.^[^ ^319^ ^]^ These scaffolds offer structural integrity and support, while also delivering physical and chemical signals to promote cell and tissue growth.^[^ ^320^ ^]^ Therefore, scaffolds hold significant potential for creating functional tissue substitutes and mimicking essential biological processes.

Electrospun fibers have attracted significant attention as scaffolds in tissue engineering, owing to their numerous advantages.^[^ ^305^ ^]^ However, as their applications continue to expand, single‐function electrospun fiber scaffolds are no longer sufficient to address the growing complexity of demands.^[^ ^306^ ^]^ This has spurred the development of stimuli‐responsive and functionalized fiber scaffolds, designed to meet higher functional requirements and broaden their potential applications in tissue engineering.^[^ ^321^ ^]^ Electrospun nanofibers, owing to their high specific surface area, microporosity, strong drug‐loading capacity, and controlled release properties, can effectively mimic the ECM and have found widespread use in tissue engineering.^[^ ^293^ ^]^ Their applications in tissue engineering include vascular,^[^ ^322^ ^]^ nerve,^[^ ^323^ ^]^ musculoskeletal,^[^ ^324^ ^]^ cardiac,^[^ ^296^ ^]^ and wound healing.^[^ ^39^ ^]^ Researchers have employed various techniques to modify fiber scaffolds, such as pre‐modification, post‐modification, and the preparation of fiber composite membranes, to meet diverse application scenarios and requirements.^[^ ^325^ ^]^ These modification strategies further enhance the performance of electrospun fiber scaffolds, making them more suitable for a wide range of tissue engineering applications. Notably, electroactive electrospun fiber scaffolds play a vital role in facilitating electrically active tissue regeneration such as nerves, cardiac tissue, and skin.^[^ ^163^ ^]^ Functionalized electroactive fiber scaffolds can integrate physical, chemical, electrical, and topographical properties, effectively mimicking the electrophysiological microenvironment to support tissue regeneration under diverse pathological conditions.^[^ ^292^ ^]^

Electroactive materials involved in EEN scaffolds can be categorized into two types based on the form of electrical signal generation: conductive and piezoelectric. The following sections will explore these two types of electroactive materials and the fabrication of EEN scaffolds, with an emphasis on their applications in tissue engineering.

#### 5.2.1 Conductive Biomaterials

**Figure 4:**
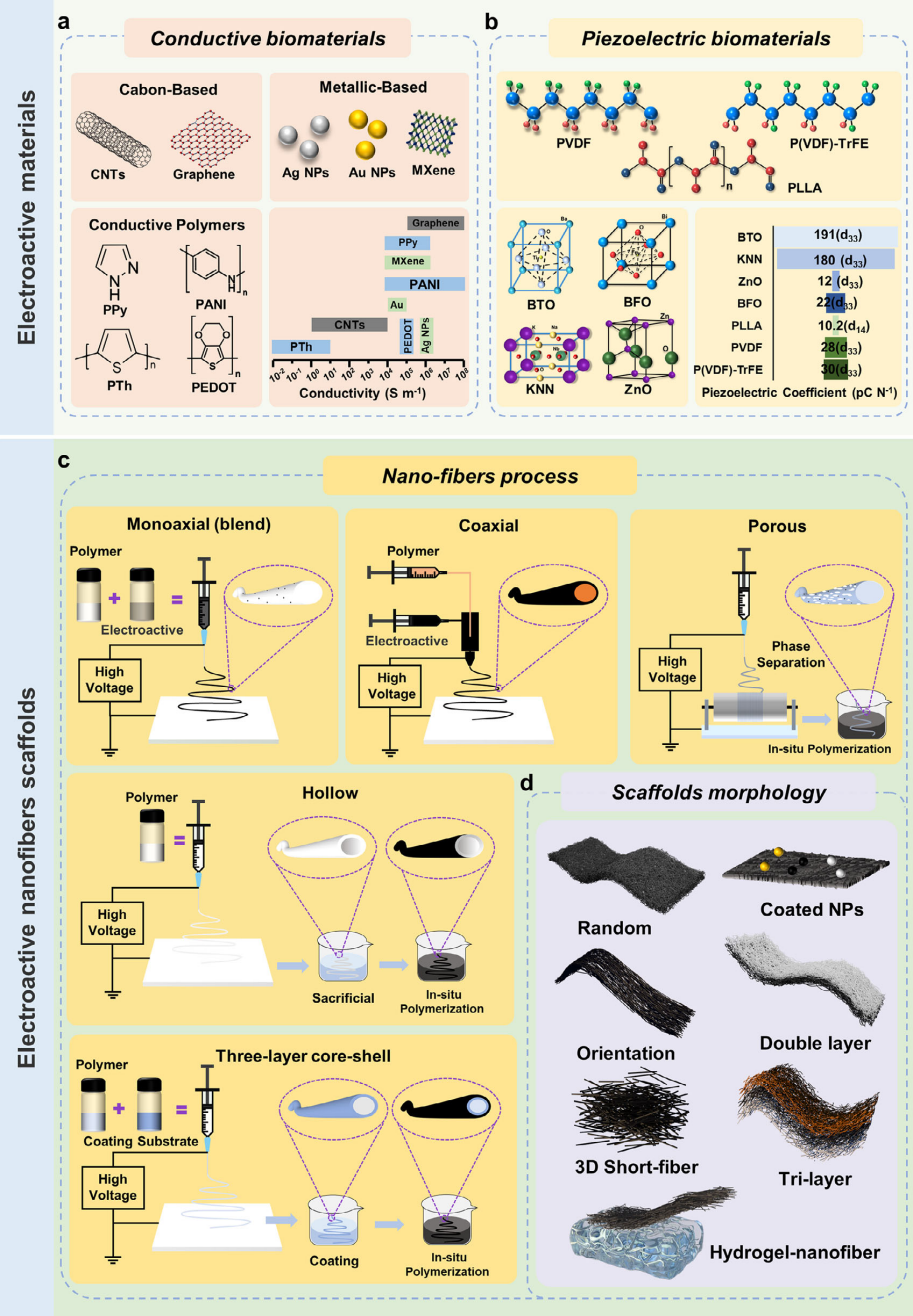
a) Conductive biomaterials and conductivity. b) Piezoelectric biomaterials and piezoelectric coefficient. c) Schematic diagram of the preparation of electroactive nanofibers. d) Schematic diagram of the electroactive nanofiber scaffold morphology.

Conductive materials are crucial for the construction of effective conductive scaffolds.^[^ ^326^ ^]^ Studies have shown that these materials facilitate charge transfer at the cell‐substrate interface and regulate both cell‐substrate and cell‐cell interactions, thereby promoting cell adhesion, proliferation, self‐renewal, differentiation, and signal transduction.^[^ ^327^, ^328^ ^]^ Fundamentally, this is closely tied to the bioelectric effects of conductive materials, as discussed in Section 3. Conductive biomaterials used in the fabrication of electroactive electrospun fiber scaffolds can be broadly classified into carbon‐based materials, conductive polymers, and metal nanomaterials (**Figure** **4**a).

Carbon‐based materials have long been at the forefront of engineering advancements.^[^ ^329^ ^]^ These materials exist in various carbon allotropes, including three‐dimensional graphite, two‐dimensional graphene, one‐dimensional carbon nanotubes (CNTs), and zero‐dimensional structures like carbon dots (C‐dots), fullerenes, and nanodiamonds.^[^ ^330^ ^]^ Each carbon‐based biomaterial exhibits distinct chemical and physical properties owing to differences in size, mechanical performance, and surface chemistry.^[^ ^329^, ^330^ ^]^ These materials offer key advantages, such as low cost, high electrical conductivity, large specific surface area, chemical stability, and mechanical durability, making them widely utilized in tissue engineering for fabricating conductive fibers.^[^ ^331^ ^]^ CNTs and graphene are the most commonly used, while carbon dots, fullerenes, and nanodiamonds remain underexplored for conductive fiber scaffolds in tissue engineering. For instance, Liu and colleagues developed CNT‐incorporated oriented PELA fibers for cardiac tissue engineering using both blending and coaxial electrospinning.^[^ ^332^ ^]^ Their findings revealed that when the CNT content reached 3%, the conductivities of the fibers prepared by these two methods were ≈30.7 and 26.1 ms cm^−1^, respectively, with the blended fibers demonstrating higher conductivity than the coaxial fibers.^[^ ^332^ ^]^ However, attention must be given to the dispersion of carbon nanomaterials, as randomly dispersed carbon nanomaterials tend to form microaggregates, which can reduce the electrical conductivity of carbon nanotube‐based scaffolds.^[^ ^333^ ^]^ To address these issues, carbon‐based materials are typically modified with chemical functional groups such as redox, carboxyl, and dopamine.^[^ ^334^ ^]^ Graphene exhibits superior electrical conductivity, high light absorption, excellent thermal conductivity, and remarkable mechanical strength compared to any other carbon material at room temperature, making it highly valuable in numerous applications. Graphene oxide (GO) and reduced graphene oxide (rGO) are the most extensively studied covalently functionalized forms of graphene.^[^ ^335^ ^]^ Zhou et al. developed nanofiber scaffolds with oriented microstructures and electroactivity using PCL and GO for nerve injury reconstruction.^[^ ^336^ ^]^ The four‐point probe method results demonstrated that the 0.5% GO/PCL nanocomposite scaffold achieved a higher conductivity, reaching up to 0.09 ± 0.01 S m^−1^.^[^ ^336^ ^]^ Naturally, during the graphene modification process, the introduction of various functional groups or increased oxygen content may adversely affect conductivity, and this must be carefully considered in the design phase.^[^ ^337^ ^]^ Compared with sole graphene and its derivatives, graphene‐based composite scaffolds can effectively reduce the cytotoxicity caused by direct contact between graphene and cells, showing promise for the design of electroactive scaffolds suitable for introducing ES.^[^ ^337^ ^]^ Additionally, the biocompatibility of carbon nanomaterials remains a topic of debate, and their potential cytotoxicity must be thoroughly evaluated to ensure both safety and efficacy in tissue engineering applications.^[^ ^338^ ^]^ Overall, carbon‐based biomaterials have garnered significant attention from researchers due to their cost‐effectiveness, ease of fabrication, wide availability, and excellent properties. For tissue engineering applications, carbon‐based biomaterials are commonly used as reinforcing agents to increase the mechanical performance and conductivity of polymer matrix.^[^ ^339^ ^]^ And in the fabrication of electroactive fiber scaffolds for wound healing not only regulates the mechanical properties of the scaffolds but also exhibits antibacterial properties and reactive oxygen species (ROS)‐clearing capabilities.^[^ ^340^ ^]^ As vital biomaterials in regenerative medicine, future research should focus on further exploring modification strategies and synthesizing novel carbon‐based materials to enhance their biocompatibility and broaden their applicability.

Metal‐based biomaterials demonstrate exceptionally high electrical conductivity (10^5^–10^7^ S m^−1^), attributed to the presence of free valence electrons.^[^ ^333^, ^337^ ^]^ In comparison to traditional bulk metallic materials (e.g., titanium alloys, magnesium, stainless steel, and cobalt‐chromium alloys), newly developed metal‐based biomaterials provide several advantages, such as micro/nanostructures, large specific surface area, and ease of surface modification.^[^ ^341^ ^]^ These properties allow for binding with bioactive agents, thereby enhancing the regenerative efficiency of tissue engineering.^[^ ^333^, ^337^ ^]^ Gold and silver nanomaterials are among the most widely used metal‐based biomaterials in tissue engineering. Typically employed as conductive fillers in nanoparticle form, they improve the electrical conductivity and mechanical strength of tissue engineering scaffolds.^[^ ^163^ ^]^ Furthermore, owing to their inherent physical properties, such as a large specific surface area, and chemical properties, including an easily modifiable surface, metal‐based materials can effectively serve as drug carriers, further enhancing their functionality.^[^ ^163^, ^337^ ^]^ For instance, McKeon‐Fischer and colleagues developed conductive, biocompatible, and biodegradable scaffolds for skeletal muscle tissue engineering by combining poly(L‐lactic acid) (PLLA) with varying concentrations of gold nanoparticles (Au NPs) via electrospinning.^[^ ^342^ ^]^ The 21% Au‐PLLA ratio scaffold exhibited an output current of 0.032 ± 0.010 A and a conductivity of 0.094 ± 0.037 S cm^−1^. Similarly, Shahmoradi S et al. fabricated a porous PCL‐GO/Ag/Arg dressing by incorporating graphene oxide, silver nanoparticles (Ag NPs), and poly‐L‐arginine (Arg) through electrospinning.^[^ ^343^ ^]^ Compared to Ag NPs, Au NPs offer superior biocompatibility, stability, and easier functionalization with various organic and biological molecules.^[^ ^337^ ^]^ Their strong light absorption and scattering properties make them suitable as contrast agents in imaging technologies, aiding in the visualization and monitoring of wound tissue engineering.^[^ ^340^ ^]^ As a result, their applications are more diverse. Conversely, silver nanomaterials are primarily employed in the antibacterial field, contributing to wound healing through their exceptional antibacterial properties.^[^ ^340^ ^]^ However, careful attention must be given to the concentration of silver nanoparticles and the potential biological toxicity arising from Ag⁺ release.^[^ ^335^ ^]^ While metal‐based biomaterials are extensively utilized in tissue engineering, their long‐term biocompatibility and biodegradability in natural tissue environments remain critical concerns. Recently, two‐dimensional transition metal carbides and nitrides (MXene) and gallium‐based liquid metals (LM), as a new generation of metal‐based materials, have shown advantages in fluidity, electrical conductivity, thermal stability, and biocompatibility.^[^ ^344^, ^345^ ^]^ These materials are poised to become the leading candidates for the preparation of conductive scaffolds in tissue engineering.

Conductive polymers are a class of organic materials characterized by a conjugated backbone with alternating single and double bonds, offering conductivity comparable to semiconductors (10^−7^ to 10^3^ S cm^−1^).^[^ ^338^, ^346^, ^347^ ^]^ These materials often require doping agents or surface functionalization to enhance their conductivity, as undoped conjugated polymers possess very limited inherent conductivity.^[^ ^348^ ^]^ The size, functional groups, and charge distribution of the dopant (counter‐ion) play a critical role in determining the conductivity and overall performance of conductive polymers.^[^ ^346^ ^]^ Conductive polymers are widely applied in various biomedical fields, such as polypyrrole (PPy), polyaniline (PANi), polythiophene (PTh), and poly(3,4‐ethylenedioxythiophene) (PEDOT).^[^ ^346^ ^]^ However, these materials inherently possess limitations, such as brittleness and poor processability.^[^ ^338^ ^]^ They are typically chemically modified or physically blended with non‐conductive polymers (e.g., PCL, polyurethane (PU), PLLA) to enhance their mechanical properties, processability, and conductivity, thereby making them more suitable for fabricating fiber scaffolds in tissue engineering.^[^ ^346^ ^]^ For example, Feng et al. utilized stable jet electrospinning and two rounds of in situ polymerization to prepare aligned PPy/PDA/PLLA for nerve regeneration studies.^[^ ^323^ ^]^ PPy and its derivatives are highly valuable in tissue engineering, primarily due to their exceptional biocompatibility and stimuli‐responsive properties.^[^ ^337^ ^]^ However, the incorporation of PPy increases the hydrophobicity of the designed composites, negatively affecting cell attachment. After chemical modification, PPy exhibits improved hydrophilicity and enhanced adhesion to human fibroblasts and Schwann cells.^[^ ^335^ ^]^ Additionally, PPy demonstrates photothermal properties, enabling combined light, heat, and electrical therapies.^[^ ^349^ ^]^ PANi is one of the commonly used conducting polymers due to its potential biocompatibility, low cost and high conductivity, environmental stability, simple acid‐base doping/dedoping process, and reversible reoxidation.^[^ ^350^ ^]^ In addition, PANi is pH‐responsive. In terms of wound healing, it can be used to make pH‐switchable conductive biomaterials and to characterize the wound environment.^[^ ^350^ ^]^ Rajasekaran et al. optimized PANi and incorporated it into SF/PCL solutions to fabricate electroactive microfibers through direct electrospinning, demonstrating stability and potential as electroactive biomaterials for wound healing applications.^[^ ^35^ ^]^ PTh has good conductivity, optical properties, and chemical stability.^[^ ^351^ ^]^ Park et al. integrated chemically synthesized PTh NPs into PCL nanofibers, showing their feasibility as tissue scaffold materials.^[^ ^352^ ^]^ PEDOT is a derivative of PTH and has better thermal stability and higher conductivity than PPy, and better electrochemical stability than PTH.^[^ ^353^ ^]^ Beregoi and collaborators developed a straightforward method to fabricate electrospun nanofibers based on poly(3,4‐ethylenedioxythiophene) doped with PEDOT, which served as triggers for fibroblast differentiation.^[^ ^354^ ^]^ In addition, it is optically transparent and is a potential material for the preparation of visualized electroactive nanofiber scaffolds, and the conductivity of PEDOT can be further enhanced through heat treatment, light treatment, or the addition of ionic liquids, thereby providing new materials for wound healing.^[^ ^353^ ^]^

Additionally, conductive polymer‐based scaffolds share a common challenge with carbon‐based biomaterials—difficulty in achieving complete degradation in vivo. To address this challenge, a grafting strategy has been proposed, wherein conjugated oligomers are grafted onto the backbone of biodegradable natural or synthetic polymers to enhance biodegradability. For example, Rivers et al. synthesized a novel conductive biodegradable polymer by linking conductive oligomers of thiophene and pyrrole through degradable ester bonds, demonstrating potential for use in tissue engineering.^[^ ^355^ ^]^ Several research teams proposed using aniline trimers, tetramers, and pentamers as base materials, synthesizing inherently electroactive biodegradable polymers by linking them with polylactic acid or ester bonds.^[^ ^355^, ^356^, ^357^ ^]^ Some other research teams have also effectively utilized the excellent electronic properties and high photoelectric conversion efficiency of the conductive polymer poly(3‐hexylthiophene) (P3HT) to convert light into electrical signals via photoelectric stimulation, providing a highly biocompatible and effective approach to overcoming the limitations of current strategies that rely on modifications to the conductive component.^[^ ^358^, ^359^ ^]^ This concept of converting light energy into electrical energy has been explored in neural tissue engineering, but no relevant studies have been found in the context of wound healing.^[^ ^360^, ^361^, ^362^ ^]^ The idea of using light‐controlled electroactive nanofiber scaffolds to promote wound healing holds great potential for improving healing efficiency, as it leverages both light and electricity. However, a key challenge lies in enhancing the photoelectric conversion efficiency of these systems.

Therefore, despite certain limitations, chemical modifications have significantly improved the biocompatibility, mechanical properties, and biodegradability of conductive polymers, making them more suitable for tissue engineering applications. Compared to carbon‐based and metal nanomaterials, conductive polymers present a more balanced combination of mechanical properties, biocompatibility, and conductivity, which has contributed to their growing use in tissue engineering.

#### 5.2.2 Piezoelectric Biomaterials

Exogenous electrical stimulation based on conductive biomaterials usually relies on large external power sources, which causes inconvenience to patients and increases the risk of infection after implantation.^[^ ^22^ ^]^ Advances in medical technology have led to the trend of power source miniaturization.^[^ ^363^ ^]^ Piezoelectric biomaterials are smart materials that generate an electric field by changing surface charge in response to mechanical deformation, making them suitable for use in wireless electrical stimulation generators.^[^ ^364^, ^365^ ^]^ Piezoelectric nanofiber scaffolds, which can promote tissue regeneration by wireless electrical stimulation, have garnered significant attention in tissue engineering.^[^ ^210^, ^364^ ^]^ Piezoelectric biomaterials have been utilized in diverse biological applications and are generally categorized into natural piezoelectric biomaterials (e.g., amino acids, peptides, proteins, and polysaccharides) and synthetic piezoelectric biomaterials (e.g., ceramic, polymer, and composite materials).^[^ ^161^ ^]^ Piezoelectricity is an inherent property of piezoelectric biomaterials, indicating the strength of their piezoelectric effect. It is typically quantified by the piezoelectric coefficient $d_{ij}$, and the relationship between this coefficient with electric displacement or field, stress or strain can be written in the following form:^[^ ^161^, ^366^ ^]^

(11) $$d_{ij}=\left(\frac{\partial D_{i}}{\partial T_{j}}\right)=\left(\frac{\partial S_{j}}{\partial E_{i}}\right)$$

and it can also be converted into the following matrix to represent:

(12) $$dij=\left[\begin{array}{c} 0\\ 0\\ d_{31} \end{array}\begin{array}{c} 0\\ 0\\ d_{32} \end{array}\begin{array}{c} 0\\ 0\\ d_{33} \end{array}\begin{array}{c} 0\\ d_{24}\\ 0 \end{array}\begin{array}{c} d_{15}\\ 0\\ 0 \end{array}\begin{array}{c} 0\\ 0\\ 0 \end{array}\right]$$

where *D* is the electric displacement, *T* is the stress, *S* is the strain tensor, *E* is the electric field, is the electric displacement. The value of i ranges from 1 to 3, representing the direction in which the electric field is applied or generated, while j ranges from 1 to 6, denoting the direction of the applied stress or resulting strain. In the literature, the longitudinal piezoelectric coefficient (*d_33_*), the transverse piezoelectric coefficient (*d_31_*), and the tangential piezoelectric coefficient (*d_15_*) are commonly reported.^[^ ^161^ ^]^ The piezoelectric coefficient of natural piezoelectric biomaterials typically ranges from 0.1 to 10 pm V^−1^, limiting their applications in tissue engineering.^[^ ^367^ ^]^ In contrast, synthetic piezoelectric biomaterials are more commonly used in tissue engineering scaffolds due to their higher piezoelectric coefficients and excellent electromechanical coupling properties.^[^ ^161^, ^210^ ^]^

Ceramic materials are typically categorized into lead‐based and lead‐free piezoelectric ceramics.^[^ ^368^ ^]^ In biomedical applications, lead‐based materials are predominantly used in medical devices, whereas lead‐free ceramics are favored for in vivo applications due to the biological toxicity associated with lead‐based materials.^[^ ^369^ ^]^ To date, lead‐free piezoelectric ceramics, including BaTiO₃ (BTO),^[^ ^370^ ^]^ K₀.₅Na₀.₅NbO₃ (KNN),^[^ ^371^ ^]^ BiFeO₃ (BF),^[^ ^372^ ^]^ and ZnO,^[^ ^205^ ^]^ have been reported for use in electroactive electrospun fiber scaffolds for tissue engineering (Figure 4b).^[^ ^364^ ^]^ Existing studies demonstrate that lead‐free piezoelectric ceramics are commonly combined with natural polymers to produce electroactive electrospun fiber scaffolds. This is attributed to the inherent brittleness, high rigidity, and limited spinnability of lead‐free piezoelectric ceramics. Moreover, the poor stability, biocompatibility, and degradability of lead‐free ceramics have led to increased interest in piezoelectric polymers for tissue engineering applications, as they demonstrate superior performance in these aspects.^[^ ^161^ ^]^ Piezoelectric polymers are polarized through high‐temperature and high‐electric‐field processes to improve their piezoelectric properties.^[^ ^373^ ^]^ During electrospinning, the piezoelectric polymer jet is subjected to mechanical stretching and electric polarization, significantly increasing the proportion of the polar β phase.^[^ ^210^, ^364^ ^]^ For instance, oriented electrospun fibers produced under high rotation speed conditions exhibit a higher β‐phase content, further enhancing the piezoelectric properties of the material.^[^ ^374^ ^]^ Common piezoelectric polymers, such as polyvinylidene fluoride (PVDF) and PLLA, possess advantages including high specific surface area, flexibility, ease of processing, and biocompatibility.^[^ ^161^ ^]^ These polymers generate both mechanical and electrical signals, supporting biological processes by providing specific electromechanical stimuli.^[^ ^161^ ^]^ These characteristics render them promising candidates for tissue engineering, particularly in promoting cell proliferation and tissue regeneration,^[^ ^365^, ^375^ ^]^ thereby offering significant research and clinical potential. PVDF is a synthetic polycrystalline polymer consisting of five crystalline phases (α, β, γ, δ, and ε), with its piezoelectric properties first reported by Kawai in 1969.^[^ ^376^ ^]^ Among these phases, the β‐phase, where the hydrogen and fluorine atoms in the CH₂‐CF₂ unit are aligned along the polymer backbone and all dipole moments point in the same direction, exhibits the highest piezoelectricity.^[^ ^377^ ^]^ The piezoelectric coefficient (*d₃₁*) of standard PVDF is ≈20 pC N^−1^, making the β‐phase the most widely used piezoelectric phase.^[^ ^378^ ^]^ During the preparation of electrospun nanofiber scaffolds based on PVDF, dipoles are typically aligned within the crystalline structure of PVDF through mechanical stretching and polarization to enhance the β‐phase content.^[^ ^379^ ^]^ Comparatively, this non‐polarization treatment method provides greater stability, with the piezoelectric coefficient reaching 24 to 34 pC N^−1^ after treatment, thereby further enhancing the material's piezoelectric properties.^[^ ^379^ ^]^ Forouharshad et al. observed that electroactive fiber scaffolds made of electrospun PVDF and self‐assembling peptides (SAP) showed a maximum voltage of around 500mv when stimulated at frequencies between 100 and 500 Hz, and the seeded neural stem cells (hNSCs) showed satisfactory proliferation, viability, and differentiation.^[^ ^380^ ^]^ Furthermore, if PVDF is stabilized in the β‐phase, it can undergo direct polarization without requiring stretching. Researchers synthesized P(VDF‐TrFE) by incorporating trifluoroethylene (TrFE) units into PVDF, where the steric hindrance of TrFE induces the PVDF to adopt the all‐trans (TTTT) configuration, stabilizing it in the β‐phase.^[^ ^364^, ^381^ ^]^ Additionally, the nanofiber scaffold spun from a 20% P(VDF‐TrFE) solution at a distance of 10 cm for 2 h achieved optimal piezoelectricity, with a piezoelectric coefficient (*d_31_*) of 16.17 pC N^−1^, generating a voltage of ≈0.75 V and a current output of ≈22.5 nA.^[^ ^161^, ^382^ ^]^ However, PVDF and its copolymers are often restricted in biomedical applications due to non‐biodegradability and low biocompatibility. PLLA is another widely used piezoelectric polymer, primarily sourced from plant‐based materials.^[^ ^383^ ^]^ It is highly biodegradable and biocompatible, having been approved as a safe material by the U.S. Food and Drug Administration (FDA), which makes it ideal for a wide range of biomedical applications.^[^ ^375^, ^383^, ^384^ ^]^ The molecular structure of PLLA consists of a helical β‐crystalline conformation, where the C═O double bonds are aligned at a 125° angle to the main carbon chain.^[^ ^383^, ^385^ ^]^ When subjected to shear stress, the parallel dipoles of C═O exhibit polarity, endowing PLLA with high thermal stability and a notable shear piezoelectric coefficient (*d_14_* = −10 pC N^−1^).^[^ ^385^ ^]^ During electrospinning, PLLA dipoles align perpendicularly to the fiber axis, leading to an amorphous or electrospun phase. Research shows that the piezoelectric properties of oriented PLLA nanofibers can be enhanced through heat treatments and polarization, which effectively modify the crystalline structure and have a substantial impact on stem cell differentiation.^[^ ^386^, ^387^ ^]^ While piezoelectric polymers like PLLA offer significant advantages, including biodegradability and biocompatibility, they still face challenges such as low piezoelectric constants, limited mechanical strength, and reduced durability.^[^ ^210^ ^]^ To address these challenges, piezoelectric composites have garnered significant attention, particularly those involving polymer‐ceramic combinations.^[^ ^161^ ^]^ In the biomedical field, piezoelectric composites composed of organic polymers and inorganic nanomaterials (e.g., BTO and KNN) have been widely utilized to fabricate tissue engineering scaffolds, particularly electrospun nanofiber scaffolds.^[^ ^365^ ^]^ For instance, Chen and colleagues fabricated electrospun nanofibers by combining poly(lactic acid) (PLA) nanofibers with biodegradable, high‐performance piezoelectric KNN nanowires, enabling wireless electrical stimulation via programmed ultrasound radiation.^[^ ^388^ ^]^ Under a sound pressure of 150 kPa, the open‐circuit voltage (Voc) and short‐circuit current (Isc) of the nanofiber generator reached ≈12.09 V and ≈20.8 µA, respectively.^[^ ^388^ ^]^ Shi et al. fabricated flexible piezoelectric P(VDF‐TrFE)/BaTiO3 films via electrospinning, capable of converting ultrasound energy into electrical energy and generating a high voltage of ≈8.22 V.^[^ ^389^ ^]^

### 5.3 Design and Fabrication of EEN Scaffolds

The diverse structural designs of electroactive electrospun fibrous scaffolds are critical to their performance. These scaffolds demonstrate significant potential in biomedical applications and are also utilized in engineering fields such as energy harvesting, air purification, and electromagnetic shielding, underscoring their multifunctionality and wide‐ranging applicability.^[^ ^390^ ^]^ In this section, our discussion will focus on the preparation and key applications of electroactive electrospun fibrous scaffolds in tissue engineering, with a more detailed analysis of their role in skin wound healing presented in Section 5. Currently, electroactive electrospinning techniques generate a variety of fibers and membranes, such as hybrid, aligned, core‐shell, and hollow fibers, which are utilized in tissue engineering for the development of one‐dimensional, two‐dimensional, and three‐dimensional structures (Figure 4c,d).^[^ ^210^, ^391^ ^]^ Each preparation method has its advantages and limitations (**Table** **2**), and its practical application should be considered holistically, taking into account factors such as technical complexity, material properties, and specific research objectives.

**Table 2:** Advantages and limitations of preparation methods for EEN scaffolds in accelerating wound healing.

Manufacturing techniques			Advantages	Limitations	Reference
Nano‐fibers	Uniaxial	Direct blending	Simple device Easy production Uniform Shape	Inhomogeneous spinning solution and phase separation Insufficient formation of conductive networks	[304, 392]
		Porous (phase separation)	Large specific surface area High porosity	Uneven pore size and distribution Reduction in mechanical strength and conductivity	[393, 394]
		Hollow (Sacrifice)	Large specific surface area High porosity Strong permeability	Hollow structures may exhibit reduced consistency and mechanical strength	[393, 395]
		Three‐layer core‐shell (in‐situ polymerization)	Simple core‐shell preparation Good interface bonding Scalability	Low coating efficiency Difficult thickness control	[34, 396]
	Coaxial	Two‐layer core‐shell	Reduces adverse interactions between components Enhances multifunctionality	Viscosity and flow rate control difficult Complex equipment requirements Low production efficiency	[36, 396]
Scaffolds morphology	Single‐Layer	Random	Easy production Anisotropic properties	Two‐dimensional structure Limited mechanical properties Lacks structural guidance	[38, 397]
		Orientation	Enhanced mechanical and electrical properties along the alignment direction	Two‐dimensional structure Limited to single functionality Requires specialized equipment	[393, 398]
		Coating	Strong scalability and versatility	Coating thickness may affect electrical properties, adhesion	[399, 400]
	Double‐Layer	Janus	Hierarchical structure Enhanced structural attributes Specific functionality	Incompatibility between layers Weak interface bonding Processing complexity	[31, 401]
	3D	3D short fiber	Porous Complex 3D structure	Limited height Poor mechanical stability	[24, 396]
		Three‐layer	Multifunctional Improved mechanical properties Enhanced structural properties	Incompatibility between layers Weak interface bonding Processing complexity	[402, 403]
		Hydrogel Nanofibers	Complex 3D structure, Structural heterogeneity Improved mechanical properties Multi‐component consideration Bionic structure	Reduced conductivity Insufficient structural stability	[393, 404]

The direct mixing strategy is the most commonly used and simplest preparation method. Typically, the polymer and electroactive biomaterial are uniformly mixed in a specific ratio before being subjected to electrospinning. The method is straightforward, effectively enabling the integration of electroactive materials with fiber scaffolds, making it applicable to a wide range of tissue engineering applications. Pi and colleagues fabricated an oriented conductive fiber scaffold by blending PCL with carboxylated multi‐walled CNTs (MWCNTs‐COOH) and collected the fibers using a high‐speed rotating drum.^[^ ^405^ ^]^ The scaffold, which was loaded with brain‐derived neurotrophic factor (BDNF), significantly promoted Schwann cell proliferation, induced morphological changes, and upregulated the expression of myelination‐related genes and myelin basic protein, demonstrating the potential for nerve repair.^[^ ^405^ ^]^ Yue et al. utilized the natural polymer SF as the primary material and introduced lithium niobate (LN) nanoparticles as dopants to construct composite SF‐based piezoelectric materials.^[^ ^392^ ^]^ Additionally, doping 1 wt% MWCNTs into the LN/SF materials significantly enhanced the current signal, achieving up to 15 nA. However, direct blending may lead to uneven distribution or aggregation of electroactive biomaterials. To overcome this issue, researchers have improved needle designs to produce core‐shell, hollow, and porous fibers. Core‐shell fibers are frequently employed in tissue engineering, where electroactive biomaterials are incorporated into either the shell or core to achieve uniform distribution, enhancing the scaffold's effectiveness in tissue repair. For example, Zhang and collaborators utilized coaxial electrospinning to develop conductive core‐shell nanofiber scaffolds with a conductive shell, resulting in the highest alignment of Schwann cells and significantly promoting the growth and differentiation of PC12 cells.^[^ ^406^ ^]^ Similarly, Xu et al. applied coaxial electrospinning to fabricate Ti3C2Tx MXene/PCL/gelatin electroactive nanofibers, with the sheath solution containing 6wt.% Ti3C2Tx MXene, showing optimal electroactivity for wound healing.^[^ ^36^ ^]^ Another study has employed post‐processing techniques alongside uniaxial electrospinning to fabricate a PPy/PDA/PLLA nanofiber wound dressing, achieving a stable and uniform three‐layer core‐shell structure with enhanced conductivity, effectively addressing the limitations of coaxial electrospinning.^[^ ^34^ ^]^ However, this post‐processing approach presents challenges, including specific operational conditions, uneven material distribution, compatibility issues, and increased costs. Electroactive fiber scaffolds with hollow microstructures are relatively rare in tissue engineering applications, and their structural characteristics make their applications mainly concentrated in drug loading. In rare studies, Bhattarai et al. successfully prepared PPy hollow fibers (PPy‐HFs) by sacrificial removal of soft templates and in situ polymerization of pyrrole.^[^ ^395^ ^]^ Porous electroactive fibers have recently gained increasing attention. Yang et al. used electrospinning to create oriented porous PLLA fibers, which were then coated with PPy NPs through oxidative polymerization, resulting in porous conductive fibers with an average diameter of ≈0.82 µm.^[^ ^394^ ^]^ The porous structure formed primarily due to rapid phase separation during the rotation of a drum containing a 7.5% (w/v) PLLA solution.^[^ ^394^ ^]^ Despite their diverse internal structures, these fibers are often used to construct two‐dimensional planar structures with various morphologies in practical applications.

The preparation of two‐dimensional patterned electroactive fiber scaffolds significantly improves material utilization and enables customization of performance and functionality.^[^ ^407^ ^]^ Patterned designs allow precise control of fiber alignment and distribution, optimizing mechanical, electrical, and biological properties of scaffolds for specific tissue engineering applications, offering more possibilities for personalized treatments.^[^ ^391^ ^]^ Initially, researchers used simple metal plates as collectors to gather electroactive fiber scaffolds with random surface features, typical of electrospun fibers. To obtain electrospun fibers with different morphologies, researchers introduced various collectors, such as parallel electrode plates, drums, and discs.^[^ ^391^ ^]^ Among these methods, the drum collector is more commonly used. By adjusting the drum's rotation speed, researchers can collect conductive fiber scaffolds with either random or aligned arrangements, tailored to the requirements of various tissue engineering applications. For example, Zhang and colleagues prepared conductive composite fiber scaffolds from PCL and CNTs with varying alignment by electrospinning at different rotation speeds (0, 500, 1000, and 2000 rpm).^[^ ^408^ ^]^ At an optimal rotation speed of 1000 rpm, the electrospun nanofibers exhibited excellent alignment at both the macroscopic level (average deviation angle = 2.78°) and the microscopic crystalline scale (orientation degree = 0.73). The contact angle decreased to 99.2°±4.9°, and the fibers demonstrated adequate tensile strength in both the perpendicular and parallel directions relative to the fiber axis (1.13±0.15 MPa and 5.06±0.98 MPa, respectively). Another study employed electrospinning technology to fabricate piezoelectric PLLA nanofiber matrices, collecting scaffolds produced at rotation speeds of 300 rpm and 4000 rpm.^[^ ^398^ ^]^ It was observed that high‐speed rollers significantly enhance the crystalline and fiber alignment within the film, thereby improving both the piezoelectric and mechanical properties of the material. The study also discovered that the orientation of electrospun fibers significantly enhances their tensile strength and modulus. Compared to randomly aligned fibers, oriented fibers facilitate improved penetration and proliferation of epithelial cells and fibroblasts, which is advantageous for wound healing. Consequently, fabricating conductive scaffolds with optimized fiber orientation demonstrates the potential for synergistically enhancing wound healing through combined oriented topology and electrical stimulation.^[^ ^409^ ^]^ Additionally, researchers have leveraged the properties of different materials to create two‐dimensional planar structures with diverse morphologies. Yao and his team utilized electrospinning technology and a layer‐by‐layer deposition process to prepare rGO/PCL scaffolds with micro‐nano structures.^[^ ^410^ ^]^ Due to the immiscibility of PCL and PVP, the removal of PVP resulted in the formation of nanoscale grooves on the surface of PCL and rGO/PCL fibers, which induced neural differentiation of ADSCs and significantly promoted neural repair. Hanumantharao et al. employed electrospinning to self‐assemble nanofibers into honeycomb structures with various patterns by solely adjusting the electric field.^[^ ^411^ ^]^ These honeycomb structures provide a stable microenvironment for the infiltration and migration of human dermal fibroblasts (HDFa), thereby promoting tissue regeneration. Meanwhile, other researchers have optimized fiber structures by integrating multiple technologies. For example, Zhang and colleagues used electrospinning, surface coating, and melt electrospinning to create conductive polycaprolactone/gold (PCL/Au) scaffolds with anisotropic micro/nanostructured fibers.^[^ ^412^ ^]^ This structure regulated myoblast development and guided their differentiation into aligned myotubes, offering a novel approach to skeletal muscle tissue engineering. Tang and his collaborators prepared a biomimetic scaffold with electrical conductivity and oriented fiber structure by combining electrospinning and electrospraying techniques to co‐spray collagen and conductive PPy NPs and showed good bioactivity.^[^ ^413^ ^]^ This design supplies a platform for exploring the effects of topographical guidance, fiber conductivity, and its mediated external electrical signals on neurogenesis. Most of the electroactive nanofibers mentioned above have single‐layer structures, which makes it challenging to achieve multiple functions, such as biocompatibility, electrical properties, and topographical features simultaneously. Blending natural and synthetic polymers in an electrospinning solution can effectively overcome the limitations of each component while maximizing their advantages. However, adding electroactive materials to the electrospinning solution may compromise inherent properties like hydrophilicity, biocompatibility, and mechanical performance.^[^ ^404^ ^]^ This trade‐off must be carefully considered and optimized when designing multifunctional fiber scaffolds. Therefore, introducing conductive substances without compromising the scaffold's original advantages has become a topic of great interest.

Layered structure designs have been extensively studied in tissue engineering, as they enable the incorporation of distinct biomaterials in each layer, effectively preserving the advantages of each component while more closely mimicking in vivo tissue organization.^[^ ^319^, ^414^ ^]^ This layered design provides a promising solution for tissue regeneration by balancing biocompatibility, electrical properties, and mechanical performance. Chen and colleagues fabricated a double‐layer electroactive wound dressing by employing electrospun dendritic P(VDF‐TrFE) nanofibers as the upper layer and a conductive adhesive polyacrylamide‐gelatin double‐network hydrogel, cross‐linked with iron ions and catechol groups, as the lower layer.^[^ ^404^ ^]^ In this study, the researchers took inspiration from naturally occurring nano/microstructures. The fabricated piezoelectric nanofibers demonstrated superior mechanical properties and enhanced piezoelectric sensitivity compared to conventional P(VDF‐TrFE) nanofibers, owing to their bioinspired dendritic structures. This biomimetic approach offers new perspectives and pathways for the development of advanced tissue repair materials. Similarly, Zhang and his team designed and fabricated a Janus nanofiber scaffold featuring piezoelectric properties and a biomimetic collagen fiber alignment structure to facilitate the simultaneous healing of tendon and bone.^[^ ^415^ ^]^ The bioinspired fiber arrangement imparts considerable tensile strength and suture retention capacity to the scaffold, fulfilling the mechanical property requirements for tendon and bone repair. Moreover, the scaffold's structure offers topological guidance for cell‐cell interactions.

Post‐processing methods following electrospinning, such as coating, in situ polymerization, and combining with other scaffolds, have been widely reported. These techniques optimize the surface properties of fiber scaffolds while enhancing bioactivity, mechanical performance, and conductivity. Shevach et al. deposited Au NPs onto PCL‐gelatin hybrid nanofiber scaffolds, which significantly increased the aspect ratio of cardiomyocytes and enhanced the expression of cardiac sarcomeric actin.^[^ ^416^ ^]^ Wang et al. fabricated three‐dimensional bionic ECM‐characteristic porous short fibers by chopping electrospun fibers, freeze‐drying, and subsequently dispersing them uniformly in tertbutyl alcohol.^[^ ^398^ ^]^ Additionally, GO was homogeneously modified on these 3D biomimetic ECM‐characteristic porous short‐fiber scaffolds via π‐π conjugation, enhancing their specialized electrophysiological microenvironment. Vinikoor et al. developed an injectable, biodegradable piezoelectric hydrogel by cryosectioning short piezoelectric nanofibers of PLLA (referred to as NF‐sPLLA) embedded within a collagen matrix. This hydrogel can be directly injected into the joint, where it generates localized electrical signals under ultrasound activation to enhance cartilage healing.^[^ ^206^ ^]^ The innovation of this work lies in embedding piezoelectric nanofibers into a hydroconductive gel, which not only enables wireless stimulation but also further enhances the scaffold's natural ECM properties, promoting intracellular cell growth and facilitating the remodeling of damaged tissues. Similar approaches have recently been applied in the field of peripheral nerve regeneration.^[^ ^417^ ^]^

In summary, conductive biomaterials facilitate the conduction of endogenous bioelectricity or exogenous electrical stimuli, while piezoelectric biomaterials are valuable for constructing self‐powered, monitorable, and on‐demand wireless stimulation systems. Both types of electroactive materials are extensively utilized in the preparation of electrospun scaffolds for biomedical applications. However, careful consideration must be given to the biocompatibility, biodegradability, and potential tissue accumulation of electroactive materials. Consequently, further research into developing high‐efficiency electroactive biomaterials and refining their preparation methods is necessary to expand their clinical applications.

## 6 EEN Scaffold Strategies for Enhanced Skin Wound Healing

### 6.1 Wound Treatment

**Figure 5:**
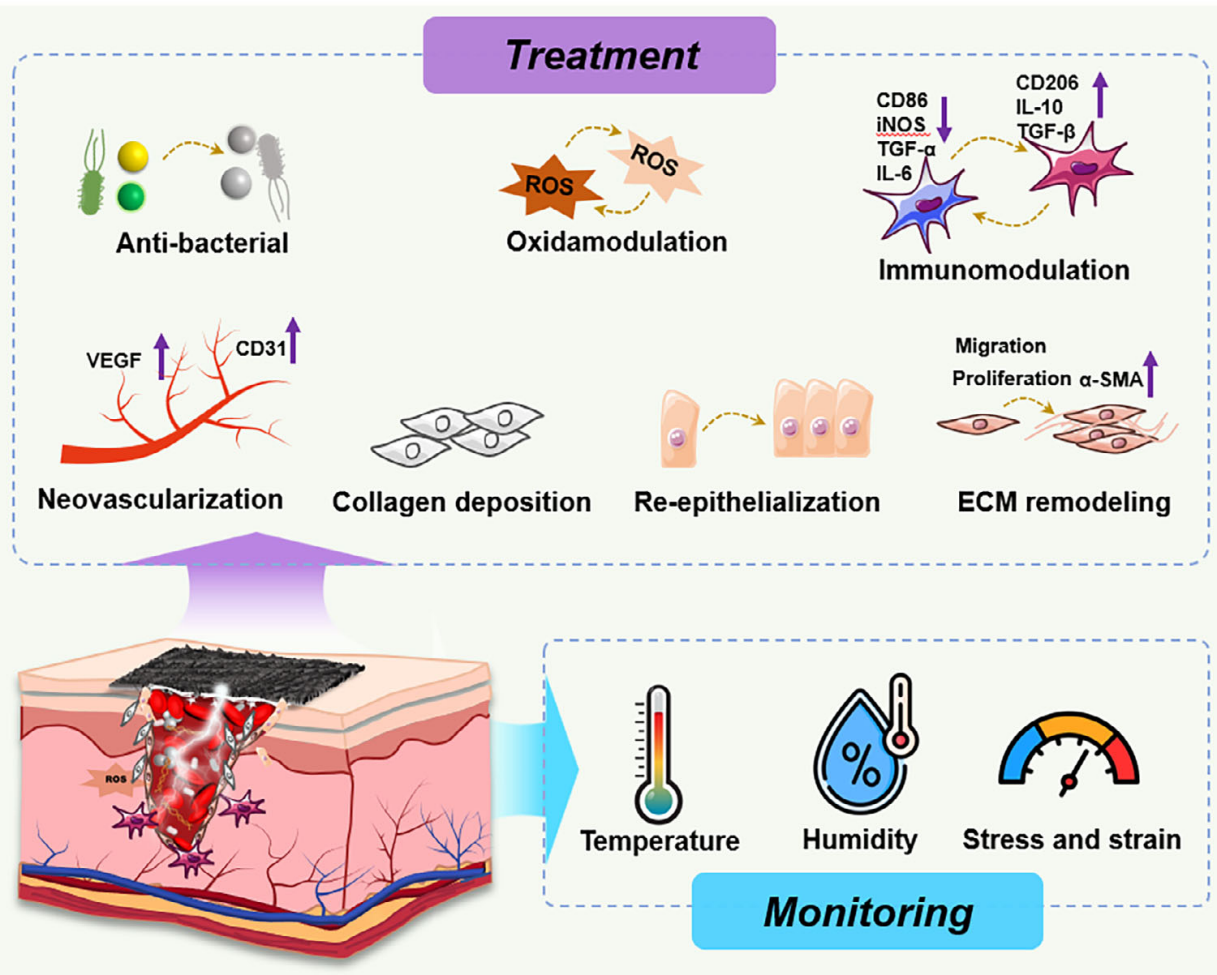
The overview of EEN scaffold strategies for skin wound healing.

Endogenous bioelectric signals play a critical role in regulating the function of electrically sensitive tissues and are indispensable in tissue regeneration processes.^[^ ^418^ ^]^ In recent years, many reviews have provided comprehensive summaries of the applications of electroactive biomaterials in tissue regeneration, with particular emphasis on recent advancements in cell fate regulation, drug delivery, and regenerative medicine.^[^ ^163^, ^419^, ^420^ ^]^ Electrospinning scaffolds based on electroactive materials—EEN scaffolds offer the distinct advantage of integrating physical, chemical, electrical, and topographical properties, thereby closely replicating the electrophysiological microenvironment of surrounding cells.^[^ ^30^ ^]^ Consequently, they demonstrate significant potential for a wide range of applications in tissue engineering. Currently, the applications of EEN scaffolds in tissue repair have been systematically reviewed in several publications, such as nerve,^[^ ^421^ ^]^ cardiac,^[^ ^422^ ^]^ and musculoskeletal.^[^ ^423^ ^]^ However, in the field of skin wound healing, although there have been numerous studies on the use of electrospun fiber scaffolds for wound healing,^[^ ^40^, ^41^, ^42^, ^148^, ^424^ ^]^ the potential of EEN scaffolds has yet to be systematically summarized. EEN scaffolds play pivotal roles in fostering fibroblast growth, re‐epithelization, vascularization, collagen deposition, immunomodulation, and mitigating complications such as infection, pain, and tissue scar formation (**Figure** **5**). Naturally, different types of wounds have different emphases on their effects.

Therefore, in this section, we will take acute and chronic wounds as the starting point and systematically summarize the latest progress and representative achievements of EEN scaffolds in promoting wound healing, especially in chronic wounds. Here, we summarize and list a variety of electroactive nanofibrous scaffolds and their material systems (**Table** **3**), and introduce the effects on the healing of different types of skin wounds in detail.

**Table 3:** Summarizes the effects of EEN scaffold strategies for enhanced skin wound healing.

Wound type	Animal models	Electroactive material systems	Design strategy	Types of electrical stimulation	Conductivity and piezoelectric output performance	Performance testing methods	Effect of skin wound healing	Refs.
Acute wounds	Full‐thickness incision in mice	PCL‐GO/Ag/Arg	Single‐layer	N/C	N/C	N/C	Promoted antibacterial activity and re‐epithelialization.	[343]
	Full‐thickness incision in mice	Ti3C2Tx MXene/ PCL/Gelatin	Coaxial single‐layer	PES	2.93 × 10⁻^3^ mS cm⁻¹	Four‐point measurement workstation	Promoted antibacterial activity, collagen deposition, and neovascularization.	[36]
	Full‐thickness incision in rats	Polyaniline doped silk fibroin‐PCL	Single‐layer	N/C	36 ± 6.1 mS cm^−1^	Four‐point measurement workstation	Increased gene expression of IL‐1β, IFNγ, Col III, EGF, TGF‐1, Col I, and FGF.	[35]
	Full‐thickness incision in mice	QCSP	Single‐layer	N/C	Two pairs of the oxidation/reduction peaks appeared at about 0.20 V, and 0.58 V, respectively.	Electrochemical workstation	Promoted radical scavenging ability and antibacterial activity.	[425]
	Full‐thickness incision in rats	PPy/PDA/PLLA	Three‐layer core‐shell	N/C	291 Ω	Resistance tester	Promoted antibacterial activity, hemostasis, antioxidant, and ROS scavenging.	[34]
	Full‐thickness incision in rats	PCL/PCE@CO/CNT	Double‐layer hydrogel nanofiber	N/C	A vertical linear curve in the low‐frequency range, a Warburg diffusion pattern in the midfrequency range, and a single depressed semicircular curve in the high‐frequency range,	Electrochemical impedance spectroscopy	Promoted antibacterial activity, collagen deposition, neovascularization, and re‐epithelialization.	[426]
	Full‐thickness incision in mice	PLLA	Single‐layer	Piezoelectric (ultrasonic)	The Voc is 0.3V 40 kHz	Oscilloscope	Promoted antibacterial activity, proliferation of fibroblast/epithelial cells, and collagen deposition	[398]
	Full‐thickness incision in mice	PVDF and PDA‐PAAm hydrogel	Double‐layer hydrogel nanofiber	Piezoelectric (self‐movement)	The Voc was 2.7 V under a 2 N force.	Electrochemical workstation	Promoted proliferation of fibroblast cells, collagen deposition, neovascularization, and re‐epithelialization.	[427]
	Full‐thickness incision in rats	P(VDF‐TrFE) and Iron ion and catechol‐based conductive adhesive polyacrylamide‐gelatin double network hydrogel	Double‐layer hydrogel nanofiber	Piezoelectric (mechanical energy)	Its output voltage and current are 0.85 V and 40 nA under peak force 6.5 N at 1 Hz	Multimeter and pico ammeter	Promoted collagen deposition and growth factor secretion.	[404]
	Full‐thickness incision in rats	PVDF and CPDA/PDA/ polyacrylamide hydrogel	Double‐layer hydrogel nanofiber	Piezoelectric (triboelectrification)	2 × 10^−2^ S cm^−1^	Four‐point measurement workstation	Promoted cell growth and migration, neovascularization, and re‐epithelialization.	[428]
	Full‐thickness incision in rats	PLLA/CFO	Single‐layer	Piezoelectric (external magnetic field)	Electrical stimulation of ≈53 mV under an intensity of 80 mT	Electrochemical workstation	Promoted collagen deposition, neovascularization, re‐epithelization, and reconstruction of granulation tissue.	[429]
	Full‐thickness incision in mice	PUE‐Zn/AB@CA	Single‐layer	Zn/AB micro batteries (redox discharge reaction)	An output current of 15 nA and an output voltage of 0.6 V	Electrochemical workstation	Promoted antibacterial activity, antioxidant, anti‐inflammatory, neovascularization, collagen deposition, and re‐epithelialization.	[37]
	Full‐thickness incision in mice	Ag/Zn@TPU/Cotton	Double‐layer	Ag/Zn (redox discharge reaction)	The peak voltage is ∼ 0.32 V	Multimeter	Promoted antibacterial activity, collagen deposition, neovascularization, and re‐epithelialization.	[430]
Burn Wounds	Deep partial‐thickness burns in mouse	PCL/ZnO	Single‐layer	Piezoelectric (LIPUS)	The Voc is 40–70mV	Electrochemical workstation	Accelerated nerve repair, collagen deposition, and neovascularization.	[431]
Infected chronic wounds	Full‐thickness skin defects in mice with *S. aureus solution*	BaTiO3/PLLA	Single‐layer	Piezoelectric (ultrasound)	The Voc is ∼ 0.4V	Electrometer	Promoted antibacterial properties, relieving inflammation, and enhancing collagen synthesis together with angiogenesis.	[432]
	Full‐thickness skin defects in rats with *S. aureus solution*	PLLA/ PEG/ BT	Single‐layer	Piezoelectric (self‐movement)	The Voc was 1.25 V under the strain of 6%	Electrometer	Promoted antibacterial activity and collagen deposition	[433]
	Full‐thickness skin defects in rats with *S. aureus solution*	PCL/Gel‐ PVDF/Ag	Double‐layer (Janus)	Piezoelectric	The Voc gradually increased from 1.49 to 5.28 V under the pressures ranging from 0.1 to 1 N	Source meter	Promoted antibacterial activity, collagen deposition, neovascularization, and re‐epithelialization. Achieved exudate management.	[31]
Diabetic wounds	Full‐thickness skin defects in diabetic rats	GelMA‐Bio‐IL and PFKU/DOXH	Double‐layer hydrogel nanofiber	N/C	0.754±0.040 S m‐1	DC power supply	Promoted anti‐inflammatory, ROS scavenging, collagen deposition, neovascularization, and re‐epithelialization.	[434]
	Full‐thickness skin defects in diabetic rats	ApF/PLCL/rG@VEGF	Single‐layer	Endogenous Electric‐Field (ion flow in injured tissue)	∼ 0.5 S m^−1^	N/C	Promoted antibacterial activity, collagen deposition, and neovascularization	[24]
Diabetes and infected wounds	Full‐thickness skin defects in diabetic rats infected with *S. aureus solution*	CFO@CTAB/PVDF	Single‐layer	Piezoelectric (dynamic magnetic field‐mechanical)	The magnetoelectric output generated could charge a capacitor with a capacity of 4.7 µF, reaching a stored voltage of 2 V within 5 min	N/C	Promoted antibacterial activity, collagen deposition, and neovascularization	[435]
	Full‐thickness skin defects in diabetic rats infected with MRSA	Cu2+/EGCG /PVDF	Single‐layer	Piezoelectric (cell adhesion)	The Voc was ∼ 4 V under the strain of 0.20 N	Oscilloscope and pico ammeter	Promoted antibacterial activity, antioxidant, anti‐inflammatory, neovascularization, and collagen deposition.	[33]
	Full‐thickness skin defects in diabetic rats infected with *S. aureus solution*	PPy/PCL and PCL/PLGA	Sandwich structure	Triboelectrification and electrostatic induction (body movement)	A maximum voltage of ≈1.5 V under a rat movement	Oscilloscope	Promoted antibacterial activity, collagen deposition, and neovascularization	[403]

#### 6.1.1 Acute Wounds

**Figure 6:**
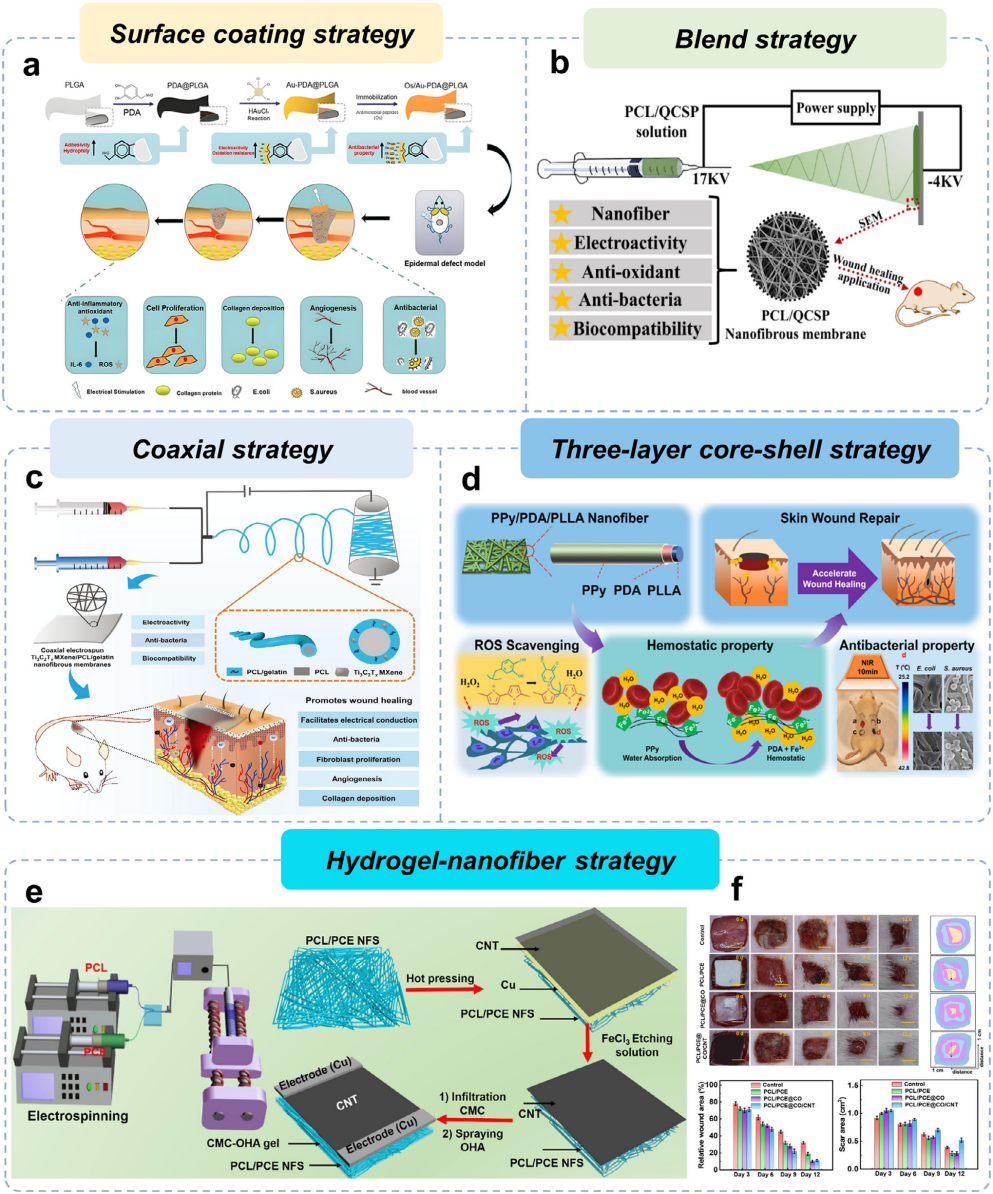
Various strategies for preparing conductive electrospun nanofibrous scaffolds for acute wound healing. a) Schematic diagram of the Os/Au‐PDA@PLGA composite membrane preparation and application in skin wound repair. Reproduced under terms of the CC‐BY 4.0 license.^[^ ^343^ ^]^ Copyright 2024, The Authors, published by BioMed Central Ltd. b) The electroactive nanofibrous membranes were fabricated by electrospinning PCL and QCSP polymer solutions. Reproduced with permission.^[^ ^425^ ^]^ Copyright 2020, Elsevier Ltd. c) Schematic diagram of preparation, characteristics, and application of the electroactive and antibacterial Ti3C2Tx MXene/PCL gelatin coaxial nanofibrous membranes. Reproduced with permission.^[^ ^36^ ^]^ Copyright 2023, Springer Nature. d) Schematic diagram of the novel PPy/PDA/PLLA three‐layer core‐shell structure and its anti‐inflammatory, hemostatic, and antibacterial properties. Reproduced with permission.^[^ ^34^ ^]^ Copyright 2022, Elsevier Ltd. e) A Schematic showing the fabrication of the high strength, excellent biocompatibility, antibacterial HNFS. f) Optical images of wound sites and wound healing rate within 12 days of different scaffolds. Reproduced with permission.^[^ ^426^ ^]^ Copyright 2023, American Chemical Society.

Acute wounds usually refer to tissue lacerations caused by sharp blade‐like instruments, with minimal collateral damage, characterized by rapid introduction of injury and a relatively rapid repair process.^[^ ^436^ ^]^ The acute wound models commonly used in existing studies are mainly full‐thickness incisions.^[^ ^437^ ^]^ In general, acute wounds follow a normal healing process, producing minimal scar tissue and having a high likelihood of healing without complications.^[^ ^436^ ^]^ However, if there is a disruption in this process, the wound is at significant risk of becoming chronic.^[^ ^438^ ^]^ Therefore, EEN scaffolds have been proposed as functional dressings to accelerate wound healing. AgNPs were designed as alternatives to traditional silver compounds to minimize tissue toxicity, leveraging their high bioactivity at low concentrations.^[^ ^340^ ^]^ For instance, Shahmoradi and colleagues leveraged the angiogenic and antibacterial properties of graphene oxide (GO), silver (Ag) nanoparticles, and poly‐L‐arginine (Arg) to create a novel porous PCL‐GO/Ag/Arg dressing via electrospinning, effectively promoting wound healing (**Figure** **6**a).^[^ ^343^ ^]^

In this study, GO was used to load Ag NPs at neutral pH (7.56) and mGA (0.168) exhibiting both the maximum loading of Ag, as well as the best distribution of Ag particles without free ions or aggregation. The results suggest that the incorporation of an appropriate dose of Arg and GO (optimal ratio obtained was 5:1) into the PCL‐GO/Ag/Arg nanocomposite can effectively enhance endothelial function via generating intracellular ROS and the phosphorylation of eNOS. After 12 d of treatment, the use of neat PCL and the PCL‐GO/Ag/Arg nanocomposites led to a decrease in wound size by 70% and 100%, respectively, while the PCL‐Ag nanocomposite had no significant impact on wound size. Despite the incorporation of Ag particles and GO, the study did not provide data on the conductivity of the prepared fiber scaffold. Furthermore, the authors acknowledged that the role of Arg was not adequately clarified, and the control group design did not effectively demonstrate its relevance. AgNPs are relatively safe, but the induced DNA damage and inflammatory response that may increase the generation of ROS in the wound bed have also been reported. In contrast, gold has a wider range of applications in material functional modification due to its excellent electrical properties, mechanical robustness, and chemical inertness.^[^ ^400^ ^]^ Although gold nanoparticles have weaker antibacterial activity than silver nanoparticles, they have stronger antioxidant and anti‐inflammatory activities and better biocompatibility and are also used as wound dressings. Although gold nanoparticles have weaker antibacterial activity than silver nanoparticles, they have stronger antioxidant, anti‐inflammatory activities and better biocompatibility, making them suitable for wound dressings.^[^ ^400^, ^439^ ^]^ Polydopamine (PDA) is commonly used as a functional coating to facilitate the in situ synthesis and loading of AuNPs. This approach effectively improves the hydrophilicity of polymeric materials and enhances their cell affinity.^[^ ^400^ ^]^ While the addition of metal nanoparticles to enhance conductivity holds promise, the limited number of scientific studies supporting their commercialization in medical practice, coupled with the high preparation cost of metal nanomaterials, hampers their widespread adoption in large‐scale clinical applications.^[^ ^440^ ^]^ Recently, two‐dimensional transition metal carbides and nitrides (MXenes) have emerged as a new generation of metal‐based materials, known for their flexibility, conductivity, thermal stability, and biocompatibility, and have been utilized in functional wound dressings.^[^ ^441^ ^]^ Xu and his team combined the mechanical properties of PCL, the bioactivity of gelatin, and the electroactivity of Ti3C2Tx MXene to fabricate nanofiber membranes (NFMs) via coaxial electrospinning (Figure 6c).^[^ ^36^ ^]^ Specifically, Ti₃C₂Tx MXene/PCL/gelatin‐6 (MPG‐6, containing 6 wt.% of Ti₃C₂Tx MXene in the sheath spinning solution) demonstrated optimal conductivity (2.93 × 10⁻^3^ mS cm⁻¹) and antibacterial activity (90.5% against E. coli and 93.16% against S. aureus). The in vivo evaluation in a full‐thickness wound defect model demonstrated that the MPG‐6 films significantly accelerated wound closure, increased granulation tissue formation, increased collagen deposition, and promoted wound vascularization. Conductive polymers, in their native state, exhibit relatively low conductivity and moderate mechanical properties.^[^ ^442^ ^]^ Strategies such as doping with acids, polar organic solvents, or ionic liquids are employed to enhance their conductivity, mechanical strength, and biocompatibility, ensuring better alignment with application requirements.^[^ ^442^ ^]^ One of the most effective strategies to address the biocompatibility issues of conductive polymers is to graft them onto biocompatible polymers and molecules and then incorporate these grafted polymers into naturally derived polymer matrices, which will produce conductive scaffolds with appropriate biological properties.^[^ ^35^, ^443^ ^]^ Rajasekaran et al. addressed these challenges by incorporating natural polymers, blending PANi with a silk protein‐loaded PCL solution to produce electroactive fibers, and demonstrating well‐organized collagen deposition, resulting in regenerated skin closely resembling natural skin. These fibers interacted with and responded to various electroactive elements in cells or skin tissues.^[^ ^35^ ^]^ This characteristic gave the material ‘smart’ properties, making it a responsive scaffold suitable for skin remodeling.^[^ ^35^ ^]^

In comparison to the use of Tegaderm^TM^, the EEN‐treated wound showed excellent tissue‐fiber integration and enhanced wound closure (∼85%) within 7 days. By day 14, both neoangiogenesis and inflammatory responses decreased, suggesting early healing and reduced chance of scare formation.By day 21, the EEN‐treated wounds demonstrated positive staining for Col III and Col I, indicating complete skin regeneration and re‐epithelialization. This study also verified the effect of pH on conductivity and found that the conductivity of the electrospun scaffold from the spinning solution containing formic acid (55.2 ± 4.8 mS cm^−1^) was higher than that from the spinning solution not containing formic acid (36 ± 6.1 mS cm^−1^). However, subsequent studies found that conductivity was not the only factor, highlighting that the mechanical properties of the scaffold are also very important. Additionally, chemical grafting methods have been reported to improve polyaniline's biodegradability. Researchers prepared antibacterial, antioxidant, and electroactive nanofiber membranes by electrospinning PCL and quaternized chitosan‐grafted‐polyaniline (QCSP) polymer solutions, which exhibited excellent free radical scavenging and antibacterial activity (Figure 6b).^[^ ^425^ ^]^ The positively charged amino and quaternary ammonium groups in QCSP can easily interact with the negatively charged bacterial and fungal cell walls through electrostatic adhesion, effectively killing bacteria, but mainly showing better bactericidal effects on *E. coli*, which may be due to the presence of PANi leading to the downregulation of essential gene expression of Gram‐negative bacteria.^[^ ^444^ ^]^ However, the cytotoxicity of PCL/QCSP NFM increases with higher QCSP content, making the balance between antimicrobial properties and cytocompatibility crucial. The study demonstrated that PCL/QCSP15 NFM, with an optimized physical structure and chemical composition, enhances wound healing and exhibits superior therapeutic effects compared to the Tegaderm^TM^ membrane and PCL/QCSP0 NFM. These findings underscore the pivotal role of scaffold electrical conductivity in wound healing. PPy is a widely used conductive polymer, known for its inherent antibacterial activity and ROS scavenging ability due to the positive charges on its main chain.^[^ ^445^ ^]^ However, PPy's brittleness limits its practical applications. To overcome this limitation, in situ polymerization on nanofiber surfaces is often used instead of directly employing PPy.^[^ ^446^ ^]^ In situ polymerization allows polydopamine (PDA) and PPy to be uniformly coated on PLLA nanofibers, creating a PPy/PDA/PLLA three‐layer core‐shell structure (Figure 6d).^[^ ^34^ ^]^ This trilayer structure provides conductivity and significantly improves hydrophilicity, near‐infrared photothermal antibacterial properties, wound hemostasis, antioxidant capacity, and ROS scavenging ability.^[^ ^34^ ^]^ On the 3rd day after treatment (wounds in the blank control group were not treated), wounds in the untreated group, the PLLA group, and the PDA/PLLA group did not recover, and even exudates appeared around the wounds, while wounds in the PPy/PDA/PLLA group showed a sign of healing and began to scab. On the 21st day, the wound area of the PPy/PDA/PLLA group had completely healed, and hair follicles and vascular tissue had appeared. Upon near‐infrared irradiation, the antibacterial properties of PPy/PDA/PLLA are further enhanced, primarily due to the photothermal effect of PPy and PDA in the near‐infrared spectrum. This improvement highlights the exceptional photothermal properties of PPy and its considerable potential in advancing comprehensive wound healing therapies. It opens up new avenues for a combined light‐heat‐electricity treatment strategy. Single‐layer EEN scaffolds often struggle to balance multiple functions, including biocompatibility, electrical properties, and topographical features.^[^ ^404^ ^]^ However, several findings demonstrate the importance of material conductivity, morphology, and procedure to electrically stimulated tissue engineering applications.^[^ ^442^ ^]^ Among different strategies, functional conductive hydrogel‐nanofiber scaffold (HNFS) composites offer a dynamic approach to wound treatment. The hydrogel nanofiber structure strategy effectively compensates for the limitations of both components and closely mimics the structure of skin tissue.^[^ ^447^ ^]^ Furthermore, the multi‐layered design endows the scaffold with scalability and multifunctionality, facilitating adaptation to various stages of the wound healing process.^[^ ^448^ ^]^ Xu and colleagues used electrospinning to fabricate a poly(caprolactone)‐poly(citric acid‐co‐ε‐polylysine) (PCL‐PCE) NFS with high mechanical strength (Figure 6e). CNTs were then grafted onto the NFS using hot pressing and ferric chloride etching techniques. Finally, carboxymethyl chitosan‐oxidized hyaluronic acid (CMC‐OHA) gel (CO gel) was introduced into the NFS via amination, forming a novel structure that maximized the benefits of both hydrogels and nanofiber scaffolds.^[^ ^426^ ^]^ It is worth noting that the mechanical strength of the PCL/PCE@CO/CNT HNFS is higher than that of commercial products (polypropylene mesh, stress: 8.05 MPa) and exceeds the mechanical strength of real mouse skin (stress: 11.05 MPa). However, it demonstrates greater versatility in terms of pore structure, air permeability, water absorption, and biodegradability. Furthermore, the rapid response rate, remarkable deformability, and commendable stability of PCL/PCE@CO/CNT HNFS when employed as a sensor endow it with substantial prospects across diverse scenarios. The cell viability of the PCL/PCE@CO/CNT group reached 148%, demonstrating significantly enhanced cellular vitality compared to the control and PCL/PCE groups, underscoring its excellent biocompatibility. By day 12, despite the wounds being minuscule, the PCL/PCE@CO and PCL/PCE@CO/CNT groups exhibited near‐complete healing, achieving ≈96.55 and 98.17% wound closure, respectively (Figure 6f). Histological analysis of wound healing demonstrated that the PCL/PCE@CO/CNT groups outperformed the control group in granulation tissue formation, vascular density, and epithelialization. However, the PCL/PCE@CO group, which lacked CNTs, also showed notable performance. This finding indicates that conductivity may not be the sole influencing factor and highlights the critical role of the scaffold's mechanical properties. Another noteworthy point is the importance of wound monitoring. While this study mentioned wearable monitoring, it did not emphasize its application in wound healing, focusing instead on its potential as a sensor.

**Figure 7:**
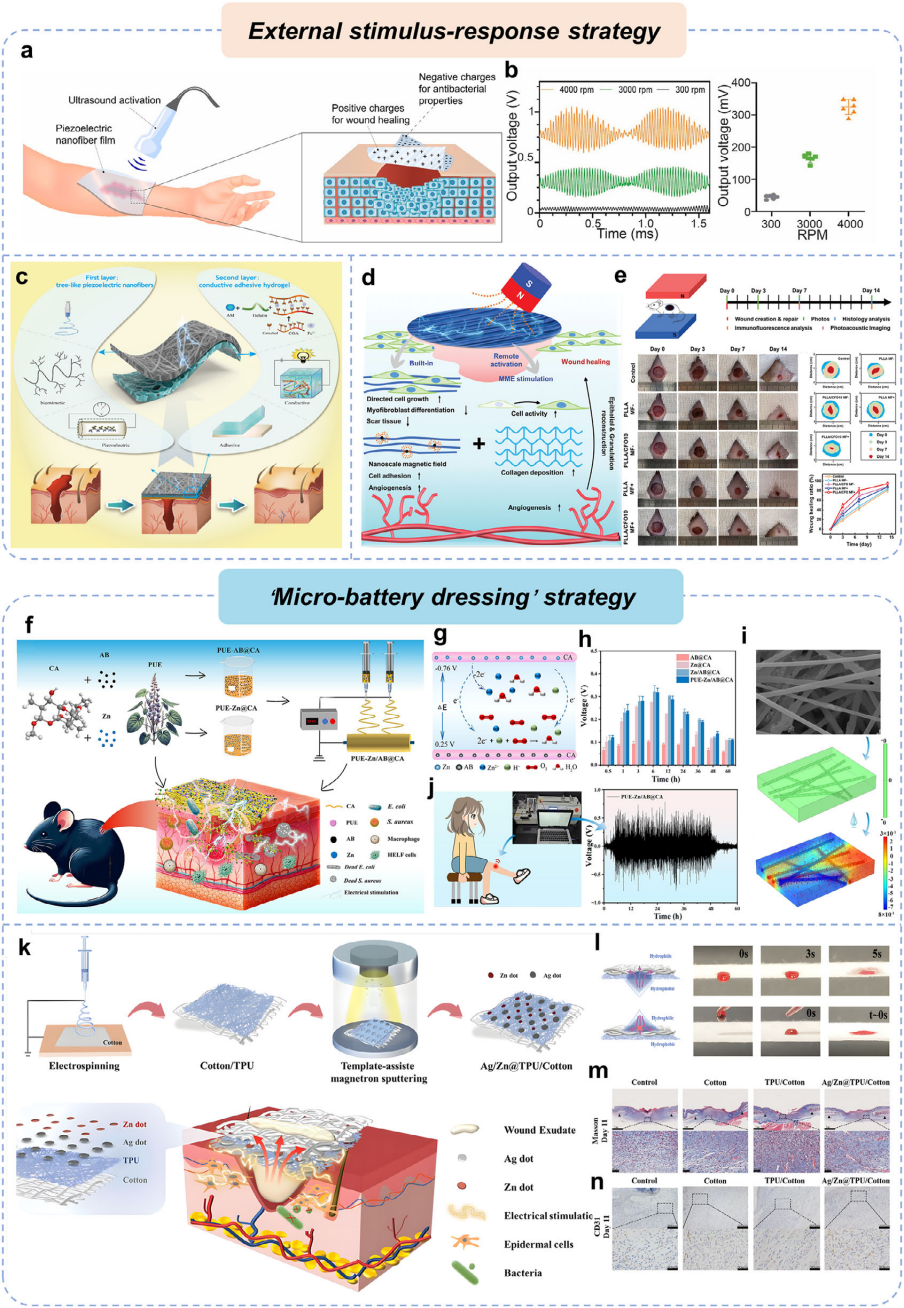
Various responsive strategies in the fabrication of piezoelectric electrospun nanofibrous scaffolds tailored for acute wound healing applications. a) Schematic diagram of the biodegradable piezoelectric nanofibers on a wound in combination with external ultrasound to produce controllable surface charge for wound healing and nanofiber orientation formed by different rotation speeds affects voltage output. b) Open circuit output voltage from PLLA transducers prepared using films at 4000, 3000, and 300 rpm collector speeds under applied 40 kHz ultrasound represented as waveforms (left) and peak output voltages (right). Reproduced with permission.^[^ ^398^ ^]^ Copyright 2023, Elsevier Ltd. c) Schematic diagram of double‐layer bionic flexible self‐powered electrical stimulator based on the force‐deformation mechanism to promote wound healing. Reproduced with permission.^[^ ^404^ ^]^ Copyright 2023, American Chemical Society. d) Schematic illustration of the mechanism of the MME cascade stimulation system constructed by the PLLA/CFO nanofibrous membrane coupled with EMF for promoting wound healing. e) The therapeutic effect of the PLLA/CFO magnetoelectric membranes on rat full‐thickness skin wound healing. Reproduced with permission.^[^ ^429^ ^]^ Copyright 2023, Wiley‐VCH. f) Preparation of PUE‐Zn/AB@CA nanofibrous dressing for wound healing and electroactive mechanism of PUE‐Zn/AB@CA. g) Electroactive mechanism of PUE‐Zn/AB@CA. h) The open‐circuit voltage of each group. i)  The SEM image and the simulated electric field in the dry (upon) and wet state (bottom). j) The open‐circuit voltage of Zn/AB@CA varies with time under bending. Reproduced with permission.^[^ ^37^ ^]^ Copyright 2024, Elsevier Ltd. k) Schematic of the manufacturing of the Ag/Zn@TUP/Cotton dressing for acute wound healing and working mechanism of Ag/Zn@Cotton/Paraffin. l) Unidirectional liquid transfer behavior of TPU/Cotton (35 min). m) Masson and n) CD31 immunohistochemical staining images of the wound treated with different dressings on day 11 in the wound site. Reproduced with permission.^[^ ^430^ ^]^ Copyright 2023, Wiley‐VCH.

The aforementioned series of studies indicate that EEN scaffolds hold great potential in promoting skin wound healing. However, most studies focus on the scaffolds' intrinsic conductivity. Some studies have combined exogenous electrical stimulation, but this approach often requires large external devices or electrode implantation near the wound, causing inconvenience and increasing infection risk.^[^ ^430^, ^449^ ^]^ To optimize the delivery of electrical stimulation, researchers have developed a series of self‐powered EEN scaffolds to accelerate wound healing. Na Yang's team utilized electrospinning to fabricate polyvinylidene fluoride‐trifluoroethylene (P(VDF‐TrFE)) piezoelectric films, achieving enhanced piezoelectric properties through the addition of tetrabutylammonium chloride (TBAC), likely due to the increased charge density and conductivity of the spinning solution (rising from 1.8 µS cm^−1^ to 800.2 µS cm^−1^).^[^ ^450^ ^]^ Additionally, the mechanical properties were enhanced, accompanied by changes in fiber morphology. Furthermore, no adverse interactions with cells or potential cytotoxicity were observed. Compared to P(VDF‐TrFE), the output voltage of P(VDF‐TrFE)/TBAC piezoelectric films was ≈5.3 times greater, with the wound healing rate showing a 30% improvement. This study unequivocally presents a chemical strategy for enhancing the piezoelectric properties of piezoelectric materials. Das et al. proposed a novel wound‐healing strategy combining electrical stimulation with tissue engineering using biodegradable, self‐potential materials like PLLA nanofiber matrices.^[^ ^398^ ^]^ This safe and stable piezoelectric scaffold can be activated by external ultrasound to generate controlled surface charges, with negative charges inhibiting bacterial growth and positive charges promoting skin regeneration (**Figure** **7**a). The voltage output of the piezoelectric nanofiber scaffolds formed at different rotation speeds is different (Figure 7b). In vitro experiments showed that compared with non‐piezoelectric PLLA (manufactured at 300 rpm) and a commercial Promogran Prisma Ag scaffold, low‐intensity/low‐frequency ultrasound‐activated piezoelectric stents can significantly promote the proliferation of fibroblasts and epithelial cells, while effectively inhibiting the growth of *S. aureus* and *P. aeruginosa*. Wound experiments also found that the healing rate of 2.74 ± 0.24 square millimeters per day was higher than that of commercial dressings.The piezoelectric nanofibers can eventually degrade inside the body as the skin heals. This biodegradability not only eliminates the requirement for undesired removal/replacement operations but also facilitates cell ingrowth into the scaffold, an advantage that cannot be achieved by most conventional piezoelectric materials such as PVDF.^[^ ^451^ ^]^ This strategy, involving the direct electrospinning of piezoelectric biomaterials to prepare electroactive nanofiber scaffolds, is among the most straightforward and widely used methods today.^[^ ^398^ ^]^ Unfortunately, while the article noted differences in the piezoelectric properties of fiber scaffolds produced at varying rotational speeds, it did not provide a comparative analysis for wound healing applications, warranting further investigation in future studies. Another approach uses a hydrogel‐electroactive electrospun nanofibrous scaffold composite, combining the wound healing benefits of hydrogels while mitigating biocompatibility issues from piezoelectric biomaterials.^[^ ^427^ ^]^ Simultaneously, it prevents the loss of fiber scaffold functionality resulting from the introduction and enhancement of ES therapy. Figure 7c shows a composite structure formed by electrospun piezoelectric nanostructures directly with conductive hydrogels, which effectively deliver ES to the wound.^[^ ^404^ ^]^ The transmission of electrical signals may involve the combined conduction of electrons and ions, thereby enhancing conduction efficiency. The P(VDF‐TrFE) nanofibers (NFs) feature a biomimetically designed dendritic structure, which enhances electrical signal generation and mechanical properties, highlighting the critical role of fiber scaffold morphology. Additionally, the underlying hydrogel structure mitigates the risk of direct contact between the piezoelectric nanofiber scaffold and the wound. Notably, this study differs from most conductive hydrogels doped with conductive materials by employing ionic conduction, which may offer enhanced biocompatibility.^[^ ^452^ ^]^ After two weeks of treatment, the wounds in the self‐powered electrical‐stimulator‐based wound dressing (SEWD) group had healed, showing significant collagen fiber deposition, skin appendage formation, and increased growth factor secretion at the wound site, all of which accelerated wound repair. In contrast, the wounds in the control and hydrogel groups showed limited healing, with closure rates of only 60% and 81%, respectively.^[^ ^404^ ^]^ Furthermore, Sharma and colleagues developed a piezoelectric‐driven triboelectric nanogenerator (PTENG) dressing. Surface charge transfer occurs during contact and release, aided by the inherent dipole moment.^[^ ^428^ ^]^ At the same time, a biocompatible hydrogel, made from polyacrylamide and PDA, was also used to provide a moist wound environment. Interestingly, the structure of carbonized PDA (CPDA) was mentioned, exhibiting electrical conductivity equal to or far superior to reduced graphene oxide,^[^ ^453^ ^]^ which enables more efficient electrical signal conduction, enhancing cell proliferation and tissue repair. In vitro and in vivo results demonstrated that, compared to the commercially available standard wound dressing (Tegaderm) and CPDA hydrogel, this structure promotes fibroblast proliferation and epithelialization, and influences endothelial cell migration in response to the electric field, leading to capillary network formation and demonstrating angiogenic potential. This study further highlights the distinctive advantages of hydrogel‐fiber composite scaffolds in promoting wound healing. The combination of piezoelectric and magnetic materials for creating self‐powered electroactive nanofiber scaffolds for wound healing has also been explored. In previous studies, magnetic or piezoelectric biomaterials have been independently employed to deliver local magnetic or electric signals.^[^ ^210^, ^454^ ^]^ Regrettably, the immobility and the resulting insufficient stress of injured sites hinder the activation of piezoelectric materials because they can only generate electrical output upon exposure to mechanical stress.^[^ ^17^ ^]^ Zhang and his team recently developed a magneto‐mechanical‐electrical (MME) cascade stimulation system using aligned magnetoelectric nanofiber membranes (PLLA/CFO) (Figure 7d).^[^ ^17^ ^]^ With the aid of an external magnetic field (EMF), the magnetic response of PLLA/CFO is amplified, inducing significant deformation and generating mechanical and electrical signals. This study fully leverages the unique properties of magnetoelectric biomaterials while preventing direct exposure of magnetoelectric nanoparticles to cells or tissues. By employing electrospinning technology, the alignment of PLLA nanofibers and the polarization of dipoles in macromolecules can be precisely controlled, thereby enhancing their piezoelectric output. However, a significant challenge lies in the influence of CFO nanoparticle content on the crystallinity of PLLA films and their piezoelectric properties, as experimentally demonstrated that PLLA/CFO10 preserved the greatest potential among the tested samples to induce electric stimulation through self‐exercise or external forces, which is highly preferable for promoting wound healing and regeneration. This system is optimized to closely align with the natural wound healing process, and animal experiment results show that aligned magnetoelectric nanofiber membranes can accelerate wound healing (Figure 7e).^[^ ^17^ ^]^ This work offers a new strategy to reconstruct the complex biophysical microenvironment in a wireless remote manner. But the article also mentioned the contribution degree of each biophysical cue remains to be determined at present, which deserves a more comprehensive and meticulous investigation. However, synthetic piezoelectric polymers are inherently non‐biodegradable and exhibit limitations in biocompatibility. Yue et al. utilized the natural polymer SF as the primary material and introduced LN nanoparticles as dopants to construct composite SF‐based piezoelectric materials. Additionally, doping 1 wt% MWCNTs into the LN/SF materials significantly enhanced the current signal, achieving up to 15 nA. This work highlights the combination of natural piezoelectric polymers and piezoelectric ceramic materials, effectively leveraging their respective advantages. Cellular experiments revealed that LN/CNTs/SF nanofiber scaffolds exhibited a significant piezoelectric effect, promoting cell proliferation with a 43% increase in the proliferation rate, ≈1.5 times higher than the blank control and SF nanofiber scaffolds, accompanied by enhanced angiogenesis.^[^ ^392^ ^]^

Considering the deformation limitations of piezoelectric biomaterials, which challenge large‐scale applications, the concept of ‘micro‐battery dressings’ has been introduced. When micro‐batteries contact bodily fluids (e.g., blood, wound exudate), redox reactions generate antibacterial and healing‐promoting effects.^[^ ^37^ ^]^ This approach offers a new direction for self‐powered EEN scaffolds. The conduction mechanism in this case may involve both electronic conduction (via the flow of electrons in the galvanic component) and ionic conduction (via ion transport in the liquid environment), with electronic conduction being the more dominant mechanism. Ionic conduction, while common in conductive hydrogel scaffolds, is exceedingly rare in electroactive nanofiber scaffolds. This rarity may be attributed to the limitations of electrospinning preparation methods. In hydrogel scaffolds, ionic conduction primarily transmits electrical signals by enhancing the ion density around target cells.^[^ ^442^ ^]^ Its advantage lies in its ability to mimic the ion transfer mechanisms present in the natural cellular environment, thereby supporting the preservation of natural electrical signal transmission between cells.^[^ ^455^, ^456^ ^]^ Additionally, it exhibits superior biocompatibility and antibacterial properties compared to electronically conductive fiber scaffolds.^[^ ^457^ ^]^ However, limitations in conductivity and sensitivity to environmental conditions increase the technical complexity of fabricating and maintaining scaffolds with stable ionic conductivity through electrospinning.^[^ ^458^ ^]^ Therefore, it predominantly exists in the form of composite conduction. For example, Chen et al. developed a drug‐loaded electroactive nanofiber dressing (PUE‐Zn/AB@CA) by incorporating zinc/acetylene black nanoparticles (Zn/AB) into puerarin/cellulose acetate (PUE/CA) nanofibers (Figure 7f).^[^ ^37^ ^]^ When wetted by wound exudate, the Zn/AB electrochemical couple is activated by potential differences (Figure 7g), generating electrical stimulation that promotes cell migration and proliferation. Moreover, this dressing can sustain stable voltage output for up to 60 h (Figure 7h), with COMSOL simulation further demonstrating significant changes in the electric field under both dry and wet conditions (Figure 7i). Simultaneously, in vivo experiments further confirmed that the open‐circuit voltage output performance remains stable even under bending conditions (Figure 7j). Possible reasons are the bending stress may facilitate internal fluid dynamics within the electrolyte, thereby removing by‐products generated from reactions and making space for new reactions.^[^ ^459^ ^]^ The results of cell experiments showed that the cell activity of PUE‐Zn/AB@CA reached 99.8%, with no significant difference compared with the Gauze group, which indirectly highlights the scaffold's advantages in promoting cell proliferation. Results from the animal wound model demonstrated that on Day 12, the healing rate of the PUE‐Zn/AB@CA group reached 96.47 ± 1.21%, surpassing the Zn/AB@CA group (94.98 ± 0.82%), CA group (93.65 ± 0.91%), and Gauze group (89.64 ± 0.84%). This study also highlights concerns regarding electroactive stability. The depletion of Zn/AB could compromise the electrical signal output, presenting a potential challenge in redox discharge reactions. However, the management of wound exudate from the wound source was not further considered here, which is known to be an important prerequisite for wound healing.^[^ ^460^, ^461^ ^]^ To address this, another study by Li and co‐workers developed a Janus‐structured electroactive dressing using electrospinning and magnetron sputtering (Figure 7k).^[^ ^430^ ^]^ The combination of hydrophobic TPU fibers and hydrophilic cotton nonwoven fabric enables efficient unidirectional fluid transfer (Figure 7l). Additionally, Ag/Zn point electrodes deposited on the TPU layer via template‐assisted magnetron sputtering are activated by exudate, generating electrical stimulation that promotes cell proliferation, angiogenesis, and wound healing (Figure 7m,n). Notably, this self‐powered system is activated by exudate through redox reactions, with initial open‐circuit and maximum voltages of ≈327.6 mV and 542.72 mV, respectively. The wound healing rate of the Ag/Zn@TPU/Cotton group was 70.71% on the 7th day, while that of the Control group was only 41.37%. The innovation of this study lies in the seamless integration of wound exudate management with electroactive properties, demonstrating significant potential for accelerating wound healing.

EEN scaffolds exhibit evolving technological potential and innovation space in promoting acute wound healing. The progression from single to composite structures, along with the transition from wired to wireless electrical stimulation delivery, highlights the continuous improvements in the diversity and performance of this technology. However, its stability and effectiveness remain to be further investigated, and innovative approaches for the preparation of electroactive nanofiber scaffolds warrant deeper exploration.

#### 6.1.2 Burn Wounds

**Figure 8:**
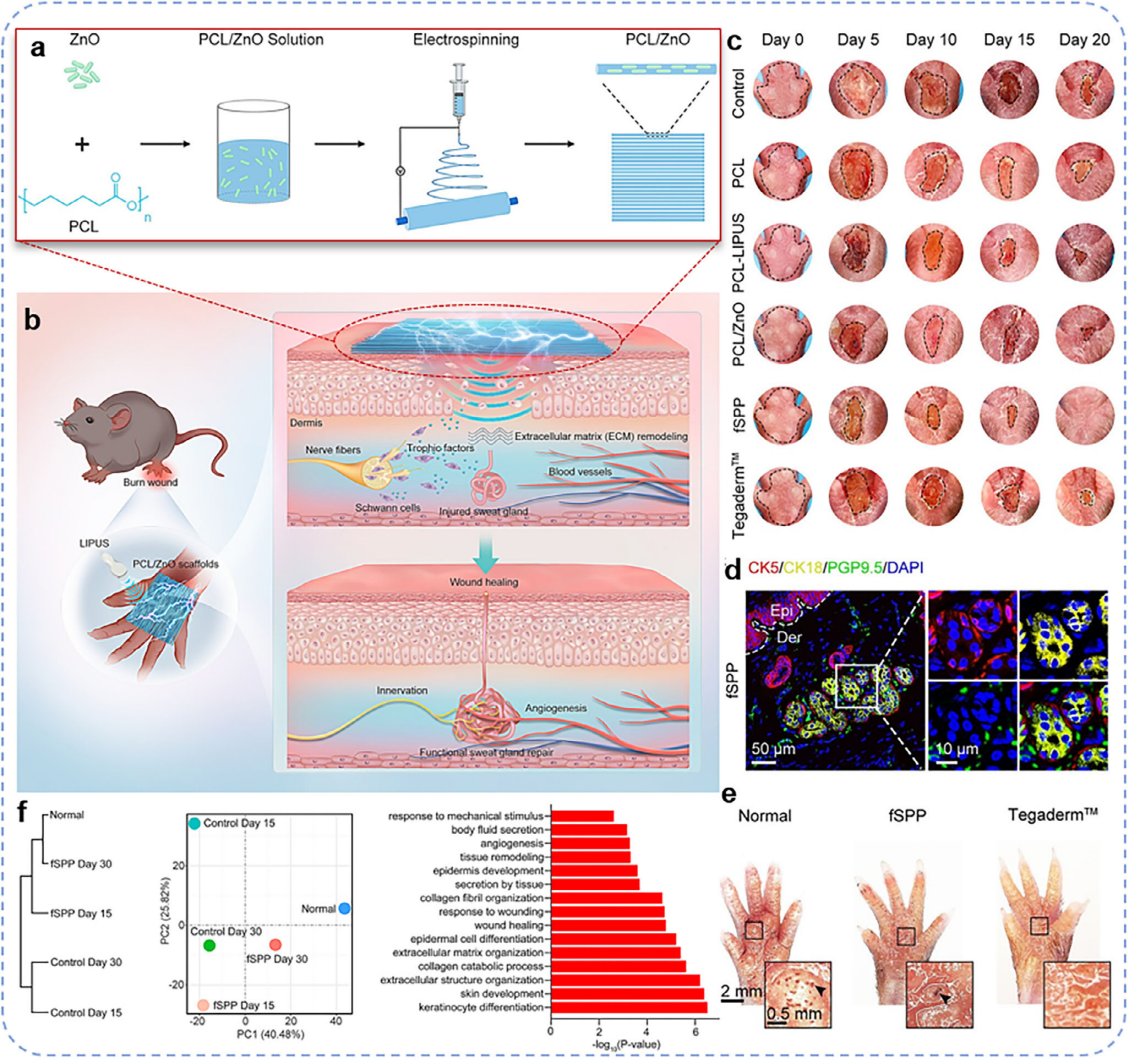
EEN scaffolds in burn wounds. a) Scheme of the detailed preparation process of the flexible sono‐piezo patch (fSPP). b)  Schematic of the fSPP for burn wound healing and sweat gland repair. c) Photographs of the burn wounds observed at various time points after surgery with different treatments. d) Immunofluorescent images of repaired sweat glands in the fSPP group on day 30. (Ductal marker CK5, red; luminal marker CK18, yellow; neuronal marker PGP9.5, green; DAPI, blue. Epi = epidermis, Der = dermis.). e) Heat‐induced sweating test on paws of mice on day 30. Positive dark spots on paw pads were detected in the fSPP group, similar to the normal group. On the contrary, no dark spot was detected in the Tegaderm group. f) PCA analysis, hierarchical clustering analysis, and KEGG pathway. Reproduced with permission.^[^ ^431^ ^]^ Copyright 2024, American Chemical Society.

Burn injuries are among the most common and destructive types of wounds, posing a significant global public health issue.^[^ ^462^ ^]^ Burn wounds are often accompanied by severe infections, excessive inflammation, reduced angiogenesis, and insufficient ECM production, all contributing to delayed healing.^[^ ^463^ ^]^ To prevent the deterioration of the wound bed and promote the healing of burn‐injured skin, it is crucial to develop innovative dressings tailored to the specific needs of burn injuries.^[^ ^464^ ^]^ However, to date, the application of EEN scaffolds in promoting burn wound healing remains limited. Recently, Pi et al. developed a flexible sono‐piezo patch (fSPP) based on electrospinning technology (**Figure** **8**a). This patch (fSPP) integrates low‐intensity pulsed ultrasound (LIPUS) and piezoelectric fiber scaffolds to repair functional sweat glands in burn wounds (Figure 8b).^[^ ^431^ ^]^ In a deep partial‐thickness burn model in mice, the fSPP system effectively closed the wound and regenerated innervated, functional sweat glands in vivo (Figure 8c–e). Notably, the fSPP group exhibited higher rates of wound closure compared with the other groups at each time point, including the Tegaderm film group. Transcriptome sequencing revealed that the fSPP system triggered a significant response in regulatory factors and functional genomics, contributing to the multifaceted remodeling of the regenerative microenvironment (Figure 8f). In addition, the fSPP‐treated regenerated skin exhibited similarities to normal skin in terms of gene expression and functional ontology relevant to neurogenesis, sweat gland regeneration, vascularization, collagen deposition, ECM remodeling, and wound healing. The research team suggested that the fSPP promoted rapid wound healing and restored functional sweat glands in burn wounds solely through external physical stimulation, presenting a potentially safe and convenient option for clinical application. However, challenges such as the non‐adhesive nature and the complexity of the treatment still need to be addressed. Furthermore, this study did not characterize the scaffold's mechanical properties, which could influence the results, nor did it evaluate its flexibility. Additionally, all ultrasound‐induced piezoelectricity may have a mixed effect.

Burn wounds, compared to other types of wounds, have unique characteristics, such as the presence of substantial exudate, which can lead to persistent inflammation, increased infection risk, tissue edema, or excessive hydration, all of which prolong the healing process.^[^ ^465^ ^]^ Although nanofiber scaffolds have been widely used to accelerate burn wound repair,^[^ ^466^ ^]^ research on incorporating electrical signals into functional fiber scaffolds for this field is lacking. Further exploration is needed to harness the full potential of electroactive materials in burn treatment.

#### 6.1.3 Infected Wounds

Wound infection is a significant barrier to normal healing and can lead to chronic, non‐healing wounds.^[^ ^467^ ^]^ Antibiotics are currently the most common strategy for treating wound infections.^[^ ^468^ ^]^ However, the rise of multidrug‐resistant microorganisms has compromised the effectiveness of many antibacterial treatments.^[^ ^469^ ^]^ Antibacterial properties are a basic requirement for wound dressings in clinical practice.^[^ ^40^ ^]^ To combat antibiotic resistance, researchers have developed antibacterial dressings by physically loading agents or embedding components through chemical reactions.^[^ ^463^ ^]^

**Figure 9:**
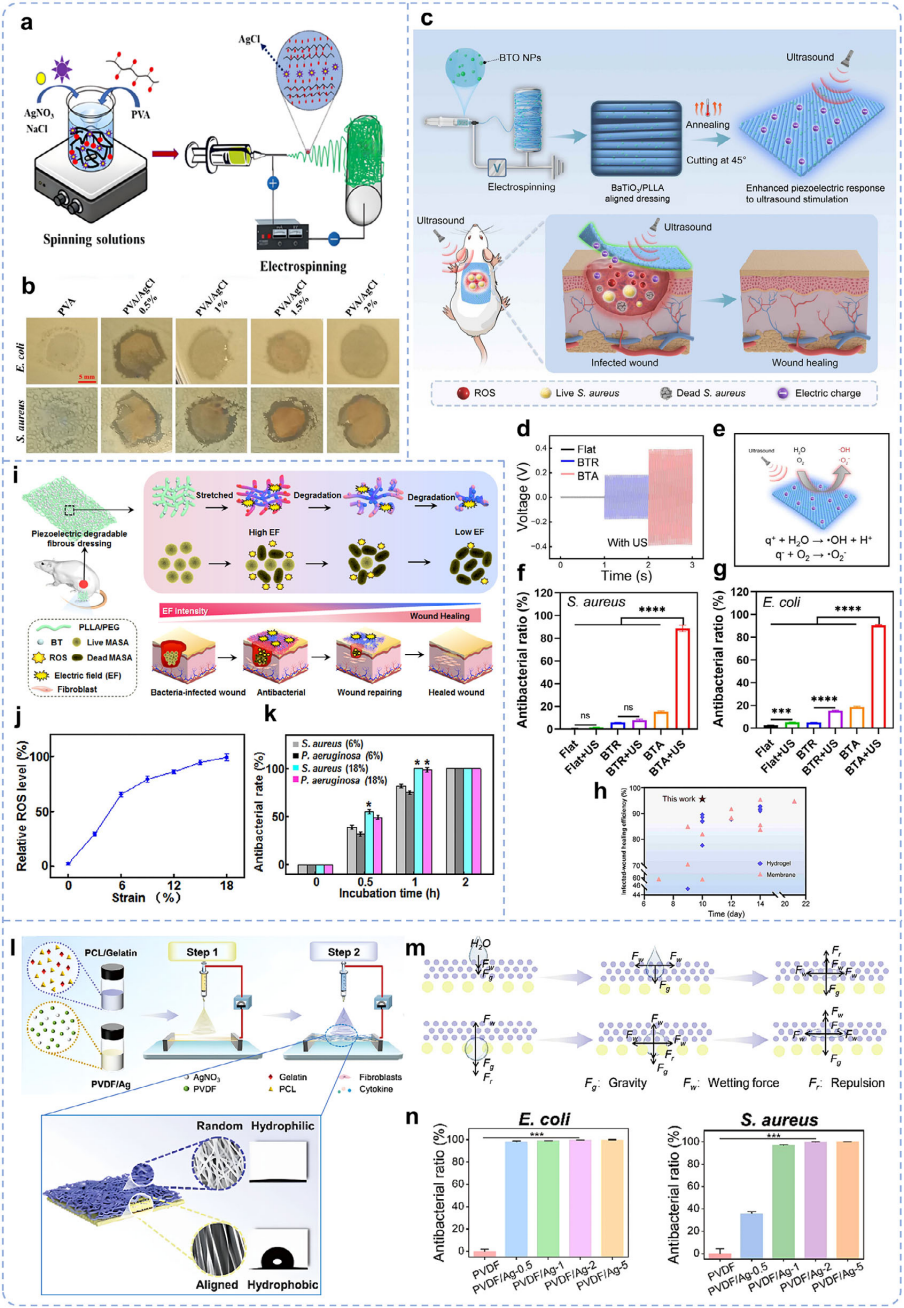
EEN scaffolds in infected wounds. a) Electrospun silver chloride‐loaded PVA nanofibers as a potential antibacterial and electroconductive scaffold for the management of wound infection and healing. b) Effectiveness of different groups in antibacterial. Reproduced with permission.^[^ ^32^ ^]^ Copyright 2024, Springer Nature. c) Schematic illustration of ultrasound‐driven BaTiO3 doped PLLA aligned piezoelectric fibrous dressing for efficient healing of infected skin wound. d) Piezoelectric output voltage of BaTiO3/PLLA electrospun fibrous dressings (EFDs). e) Schematic illustration of the generation of •OH and •O^2^– by ultrasound‐driven BaTiO3/PLLA EFDs. f,g) Quantitative analysis of antibacterial rates of *S. aureus* and *E. coli*, as assessed by live/dead staining. h) Comparison of infected skin wound healing efficiency of reported materials (red five‐point star indicate BTA EFD, blue diamonds indicate hydrogel and pink triangles indicate membrane). Reproduced with permission.^[^ ^432^ ^]^ Copyright 2024, American Chemical Society. i) Schematic illustration of the bacterial‐killing mechanism of the piezoelectric fibrous dressing. j) Relative ROS level of PLE‐BT4 under different strains up to 18%. k) Antibacterial effectiveness of PLE‐BT4 after treatment for different periods under strains up to 18%. Reproduced with permission.^[^ ^433^ ^]^ Copyright 2024, Elsevier Ltd. l) Schematic diagram of the Janus electrospun nanofibrous dressing prepared by layer‐by‐layer electrospinning of the hydrophobic PVDF/Ag and hydrophilic PCL/Gel layers. m) The mechanism of unidirectional transport of droplets in contact with hydrophilic and hydrophobic layers. n) The histograms of calculation of the antibacterial rate. Reproduced with permission.^[^ ^31^ ^]^ Copyright 2023, Springer Nature.

Metal particles like silver, copper, zinc, and their compounds are widely used in the antibacterial field, proving effective against many pathogens.^[^ ^470^ ^]^ Therefore, the development of metal‐polymer composites, with their excellent antibacterial properties and conductivity, holds the potential to further enhance the healing of various types of wounds. Considering the potential cytotoxicity of Silver compounds, Abazari's team developed polyvinyl alcohol (PVA) nanofibers loaded with silver chloride (AgCl) through electrospinning, aimed at creating antibacterial conductive wound dressings (**Figure** **9**a,b).^[^ ^32^ ^]^ PVA fibers were produced via electrospinning, with AgCl incorporated through an in‐situ reaction between silver nitrate and sodium chloride. Unlike most studies focusing solely on PVA/silver nanoparticle composites, AgCl showed excellent antibacterial activity while reducing cytotoxicity, making it a safer option for wound healing. However, the safety of silver nitrate must be carefully evaluated during the electrospinning process. Silver nitrate is a highly toxic reagent that readily oxidizes and rapidly decomposes upon exposure to light or organic substances.^[^ ^40^ ^]^

Reactive oxygen species (ROS), such as superoxide anion (O_2‐_^•^), hydroxyl radicals (OH^•^), peroxide (O_2_^2−^), and hydrogen peroxide (H_2_O_2_), play an important role in intracellular signaling for wound healing and infection.^[^ ^471^ ^]^ ROS generated at the wound site effectively kills bacteria by causing membrane collapse and cytoplasmic leakage.^[^ ^472^ ^]^ Indeed, ROS generation presents a “double‐edged sword”: excessive ROS can lead to tissue damage, while insufficient ROS may fail to provide effective antibacterial action.^[^ ^473^ ^]^ Therefore, precise regulation of ROS production is essential. Electrical stimulation has been utilized to develop control strategies that generate moderate ROS levels, providing effective bactericidal effects on skin wounds.^[^ ^474^ ^]^ Nevertheless, conventional antibacterial strategies relying on external power sources are often invasive and elevate post‐treatment risks, limiting their clinical use. Therefore, self‐powered systems have been developed. Wu et al. developed a comprehensive therapeutic modality integrating piezoelectric fibrous dressing with controlled ultrasound stimulation for efficient healing in an infected wound model (Figure 9c). The electrospun fibrous dressings composed of BaTiO_3_ doped PLLA possess improved piezoelectric properties due to the aligned structure and high crystallinity, which could effectively amplify the piezoelectric effects of PLLA and promote the generation of free radicals in response to ultrasound stimulation (Figure 9d,e). There were 88.72% and 90.43% killing rates of *S. aureus* and *E. coli* respectively upon ultrasound stimulation, with a much‐improved healing rate of 14% compared with previously reported therapeutic strategies (Figure 9f–h).^[^ ^432^ ^]^ In addition, piezoelectric‐driven therapy (PEDT) is considered another self‐powered form of portable, flexible, or wearable antimicrobial agent. This advantage stems from its ability to convert mechanical strain from mammalian activities—such as walking, running, or breathing—into electrical energy to generate ROS.^[^ ^475^ ^]^ For example, Liu and colleagues utilized PLLA, poly(ethylene glycol) (PEG), and tetragonal barium titanate (BT) as substrates to fabricate self‐powered, piezoelectric, biodegradable wound dressings with antibacterial properties using electrospinning technology (Figure 9i).^[^ ^433^ ^]^ The self‐powered source generated millivolt‐level piezoelectric signals under ≈6% and 18% strain on the rat skin in both calm and active states and maintained good biocompatibility and maximum ROS generation even under 18% strain (Figure 9j). This phenomenon may occur because stimulation of the piezoelectric material in an aqueous solution generates a micro‐electric field on its surface, disrupting hydrogen bonds in H₂O and producing ROS.^[^ ^450^ ^]^ The ROS generated by this fiber composite effectively killed 99% of bacteria within 60 min (Figure 9k). Compared to the control group, the self‐powered piezoelectric composite membrane containing 4% BT exhibited a significantly higher wound healing rate (91%) after 10 days of treatment, with complete bacterial elimination achieved within just 2 days. This study underscores the critical role of BT material proportion in enhancing the therapeutic efficacy of the self‐powered piezoelectric composite membrane.

The Janus design, with its unique bilayer structure, allows unidirectional liquid transport, effectively addressing excessive exudate accumulation in infected wounds.^[^ ^476^, ^477^ ^]^ This design is widely used in wound dressings, particularly for managing high‐exudate wounds, demonstrating significant benefits.^[^ ^478^ ^]^ Hu et al. used a simple layer‐by‐layer electrospinning technique to prepare Janus nanofiber dressings with ultrathin, flexible, breathable, and piezoelectric properties.^[^ ^31^ ^]^ The hydrophilic layer consists of randomly arranged PCL/Gel nanofibers, while the hydrophobic layer contains well‐aligned Ag NPs embedded in piezoelectric PVDF/Ag nanofibers (Figure 9l). The mechanism of unidirectional water transport in the Janus films was analyzed (Figure 9m). Antibacterial experiments found that the antibacterial ratio values showed that the pure PVDF film had a low antibacterial ratio, PVDF/Ag‐2 (2% Ag NPs) Janus dressing, the inhibition rates against *E. coli* and *S. aureus* reached 99.69% and 99.84%, respectively (Figure 9n). In the PVDF/Ag‐2 Janus group, the mice had the highest wound closure rate, especially at the early stage of healing, the scabs appeared earliest on day 3, and the wounds almost healed on day 11. In contrast, those in the control, PCL/Gel, and the TPU Janus group were still in the septic stage. However, this study does not clarify the specific role of the single‐layer PVDF/Ag‐2 dressing in antibacterial activity and wound healing, as Ag NPs may predominantly contribute to the observed effects, thereby failing to emphasize the advantages of the Janus structural design.

Bacterial growth in wound tissues is a major factor causing delayed healing, as bacteria release toxic enzymes that damage host tissues and impede wound recovery.^[^ ^479^ ^]^ From a therapeutic standpoint, the primary challenge in treating infected wounds is the complete eradication of skin and soft tissue infections caused by microorganisms like bacteria, viruses, and fungi.^[^ ^480^ ^]^ Existing studies indicate that introducing physical signals, like electrical stimulation, into tissue engineering scaffolds to create functional dressings shows promise for significant breakthroughs. However, further technological integration, along with in vitro, in vivo, and clinical experiments, is needed to validate their effectiveness.

#### 6.1.4 Diabetic Wounds

Diabetic wounds, resulting from various diabetes‐related complications, take longer to heal than normal wounds, increase the risk of infection, are prone to chronic wounds, and may even lead to amputation. Therefore, therapies aimed at promoting diabetic wound healing are of particular importance.^[^ ^481^ ^]^ Chronic diabetic wounds deviate from the normal wound‐healing process, exhibiting an abnormal inflammatory microenvironment and hypoxia resulting from vascular narrowing and diabetic microvascular complications.^[^ ^482^ ^]^

**Figure 10:**
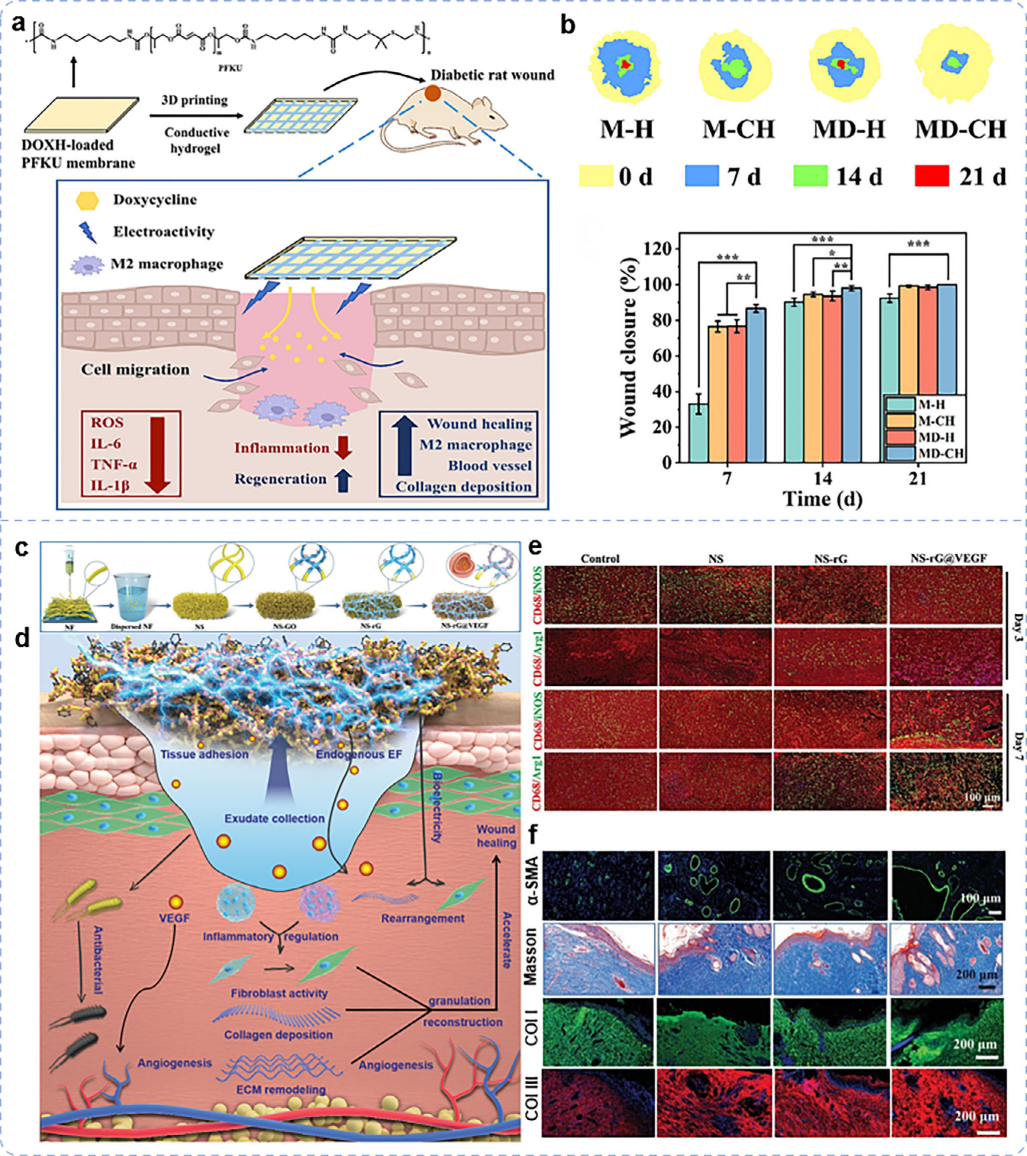
EEN scaffolds in diabetic wounds. a) A Schematic showing treatment of the diabetic wound with a composite dressing composed of conductive hydrogel strips and DOXH‐loaded PFKU fibrous membrane accelerates diabetic wound healing. b) Overlaid images (upon) and wound closure percentages (bottom) in different groups on days 0, 7, 14, and 21 post wounding. Reproduced with permission.^[^ ^434^ ^]^ Copyright 2022, Elsevier Ltd. c) Schematic illustration of the key steps in fabrication of scaffold. d) Schematic illustration of the mechanism of NS‐rG@VEGF scaffold for accelerated diabetic wound healing. e) Representative images of co‐staining of CD68 and iNOS or Arg1 on days 3 and 7. f) Immunofluorescence staining of α‐SMA, Masson, COI I, and COI III from different groups on days 21. Reproduced with permission.^[^ ^24^ ^]^ Copyright 2021, Wiley‐VCH.

In recent years, diabetic wound dressings have gained increasing attention from researchers. Electrospun membranes are regarded as excellent candidates for advanced wound dressings, as incorporating bioactive materials that regulate inflammation and promote angiogenesis significantly accelerates diabetic wound healing.^[^ ^151^, ^483^ ^]^ Meanwhile, as the role of endogenous EFs in skin wounds has garnered increasing attention, electroactive biomaterials have been developed into electrospun fibrous scaffolds for diabetic chronic wounds, exhibiting antibacterial, antioxidant, anti‐inflammatory, and angiogenic properties. EEN scaffolds have shown good prospects in promoting the healing of simple diabetic wounds. Existing studies have reported the application of double‐layer conductive hydrogel fiber and 3D bionic electroactive short fiber scaffolds in diabetic wounds. The research team led by Cao developed a two‐layer multifunctional wound dressing comprising 3D‐printed conductive hydrogel (GelMA‐Bio‐IL) strips and a ROS‐responsive DOXH‐loaded polyurethane (PFKU) membrane, specifically designed for in vivo treatment of diabetic wounds (**Figure** **10**a).^[^ ^434^ ^]^ This dressing integrates multiple functions, such as electroactivity, drug release, and ECM support, providing antibacterial activity, ROS scavenging, and enhanced cell migration, effectively regulating the abnormal microenvironment of diabetic wounds and enhancing diabetic wound healing (Figure 10b). Unlike traditional EEN wound dressings, the electroactivity of this dressing stems from the combination of conductive hydrogels with nanofibers, rather than from the inherent conductivity of the nanofibers. This study employed choline‐based bio‐ionic liquids (Bio‐IL) to fabricate conductive hydrogels, exhibiting high biocompatibility, complete water solubility, and the ability to copolymerize into polymer networks without the risk of residual monomers. However, when low concentrations of Bio‐IL were incorporated into hydrogels and printed onto nanofiber scaffolds to create wound dressings, no significant enhancement in wound healing was observed, likely due to insufficient electrical activity.^[^ ^484^ ^]^ The electrical activity primarily involves ion conduction, and its stability may be influenced by the wound environment and the water content of the hydrogel scaffold.^[^ ^434^ ^]^

Proper collection and utilization of wound exudate contribute to wound healing. Most biological scaffolds primarily focus on enhancing tissue biocompatibility and absorbing exudate to reduce infection. Therefore, whether the cytokines or ion fluxes in the exudate can be harnessed to regulate the biological functions of endogenous cells and promote tissue repair has gradually gained attention. Wang et al. developed a 3D biomimetic short‐fiber scaffold capable of early‐stage biofluid collection and coupling with an endogenous EF by guiding short fibers into a 3D network structure, followed by multifunctional modification (Figure 10c,d).^[^ ^24^ ^]^ This scaffold actively aligns with cascade reactions involved in tissue repair, enabling precise tissue remodeling. GO was homogeneously modified on 3D biomimetic ECM characteristic porous short‐fiber scaffolds by π–π conjugation. Mussel‐stimulated PDA could effectively induce the reduction of GO to reduced oxide graphene (rG) and further modify VEGF‐carrying liposomes using catechol groups as multifunctional sites in PDA. The modified rG with intrinsic conductivity formed a continuous and well‐connected 3D electrical connection network on the scaffold, thus ensuring the high conductivity of the rG‐modified nanofibrous sponge (NS–rG). The 3D biomimetic short fiber scaffold promotes water absorption and retention, enabled by its interconnected porous structure and high porosity. These features lay the groundwork for effective wound exudate management and active coupling with endogenous EF. In vitro and in vivo experimental results showed that under the influence of endogenous EF, the NS–rG suppresses inflammation, accelerates vascularization, and promotes collagen deposition and remodeling, thereby expediting diabetic wound healing (Figure 10e,f).^[^ ^24^ ^]^

**Figure 11:**
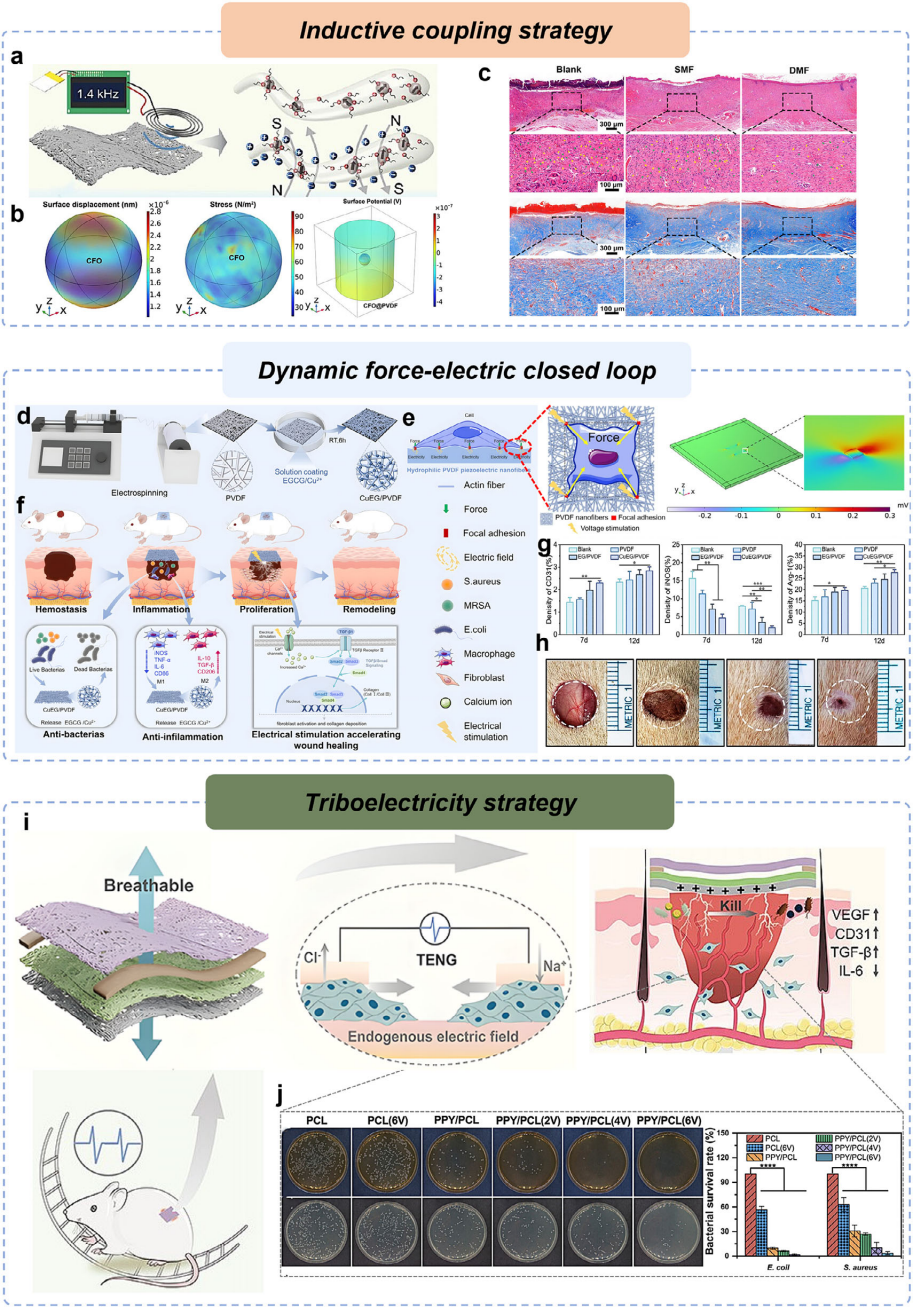
EEN scaffolds in a diabetic infected wound. a) Schematic of integrated solution involving magnetoelectric dressings and a magnetic field generator. b) COMSOL simulation of magnetostrictive effect in CFO nanoparticle and magnetoelectric coupling effect in CFO@PVDF nanofiber under a 20 Oe magnetic field. c) H&E and Masson staining images of wound tissue on the 14th‐day postsurgery. Reproduced with permission.^[^ ^435^ ^]^ Copyright 2024, American Chemical Society. d) Schematic representation of the synthesis of hydrophilic piezoelectric PVDF nanofibers. e) Schematic diagram of the force‐electric transition between cell adhesion and piezoelectric nanofibers (left). Piezoelectric potential distribution on PVDF nanofibers under 10 nN adhesion force (right). f) Application and mechanism study of CuEG/PVDF nanofibers in diabetic chronic wound healing. g) The quantification in expression amounts of CD31, iNOS, and Arg‐1 at days 7 and 14 in vivo. h) Representative photographs of wounds in CuEG/PVDF groups at days 0, 3, 7, and 12. Reproduced with permission.^[^ ^33^ ^]^ Copyright 2024, Elsevier Ltd. i) Schematic diagram of the skin patch integrated with self‐powered electrical stimulation and multifunctional properties to promote diabetic infected wound healing. j) Antibacterial activities against E. coli (upon) and S. aureus (bottom) of PCL and PPy/PCL under 0–6 V electrical stimulations by the TENG. Reproduced with permission.^[^ ^403^ ^]^ Copyright 2023, American Chemical Society.

In studies on the role of electroactive nanofiber scaffolds in promoting the healing of diabetic‐infected wounds, wireless electrical stimulation has attracted significant attention from researchers, including for diabetic chronic wounds. Currently, self‐powered systems are mainly achieved via piezoelectric and triboelectric effects. In response to external fields (e.g., magnetic or ultrasonic fields), these scaffolds generate dynamic electrical stimulation, promoting fibroblast and epithelial cell proliferation while enhancing collagen production.^[^ ^398^ ^]^ Ke et al. developed a magnetoelectric dressing (CFO@CTAB/PVDF (CCP)) by electrospinning CoFe₂O₄ (CFO) particles modified with cetyltrimethylammonium bromide (CTAB) together with PVDF (**Figure** **11**a).^[^ ^435^ ^]^ A wearable electromagnetic induction device established a dynamic magnetic field, inducing magnetostrictive deformation of CFO nanoparticles. This deformation generated a piezoelectric potential on the surface of PVDF nanofibers and achieved good magneto‐electric coupling, which is further validated by finite element simulation (Figure 11b). Further experimental results demonstrated that the magnetoelectric output could charge a 4.7 µF capacitor, achieving a stored voltage of 2 V within 5 min. Based on these electroactive properties, under dynamic magnetic and electrical stimulation, CCP dressings outperform static magnetic fields in reducing inflammatory cells, promoting granulation tissue formation, and enhancing both collagen fiber density and alignment (Figure 11c). Notably, the study also indicated that the antibacterial effect primarily originates from CTAB (which contains quaternary ammonium cations), with optimal bioactivity and antibacterial properties observed at a CTAB content of 2%.

However, electrical stimulation induced by external fields cannot be sustained for extended periods, limiting its long‐term effectiveness at the cellular or tissue level. Furthermore, the surface potential generated by polarization, being inherently static, cannot dynamically adapt to physiological cell processes and cannot synchronize with cellular activities. To address this, Ren and his team developed a piezoelectric nanofiber dressing with a dual‐network structure (Cu^2+^EGCG (Epigallocatechin 3‐gallate)/PVDF) to accelerate the healing of diabetic infected wounds (Figure 11d).^[^ ^33^ ^]^ This structure leverages cellular adhesion forces to induce piezoelectric nanofibers to generate adaptive electrical stimulation in cells, forming a dynamic force‐electric closed‐loop (Figure 11e). The results demonstrated that this dynamic force‐electric closed‐loop significantly activated cellular calcium ion activity, thereby stimulating the TGF‐β/Smad signaling pathway, promoting collagen deposition, and exhibiting notable antibacterial, antioxidant, anti‐inflammatory, and angiogenic activities (Figure 11f,g). In vivo animal experiments also showed a wound healing advantage within 12 days (Figure 11h). This study is the first to report using adhesion forces between cells and the ECM to induce piezoelectric materials to generate electrical signals, providing new insights for designing wearable, multifunctional electrotherapy devices.^[^ ^33^ ^]^ The triboelectric effect, as another form of self‐powered system, generates periodic biphasic electrical pulses by converting local mechanical displacement (e.g., body or muscle movements) into electrical energy, making it a promising candidate for producing self‐sustaining and bio‐responsive electrical stimulation.^[^ ^485^ ^]^ Electrospun fiber membranes have ideal properties for wound dressings and triboelectric nanogenerators (TENGs).^[^ ^486^ ^]^ Therefore, electrospinning polymer as a friction‐optimizing layer and chemical vapor‐deposited PPy electrodes assembled into TENG for wound healing were reported (Figure 11i).^[^ ^403^ ^]^ By harvesting mechanical motion and positive charges on the surface of PPy, the generated electrical stimulation killed over 96% of bacteria due to its synergistic effect on disrupting bacterial cell membranes (Figure 11j). On day 14 after surgery, the wounds of the TENG are entirely covered by the new pink epidermis, while the wounds of the PPy/PCL (13 ± 3%) and the Blank (36 ± 8%) are still open wounds.^[^ ^403^ ^]^ The challenges in healing diabetic wounds are mainly due to infection, inflammation, hypoxia, and poor vascularization. Current research focuses on the role of EEN scaffolds in addressing these challenges, with consistently positive results demonstrating significant potential.

### 6.2 Monitoring

Skin wound healing is a complex process with multiple parameters that continuously change during the healing period, necessitating dynamic monitoring for effective treatment.^[^ ^463^ ^]^ Traditional wound dressings rely on visual assessments during dressing changes, which cannot provide timely feedback on abnormal wound conditions and may lead to missed opportunities for optimal treatment. There is an urgent need for wound management systems capable of continuous monitoring and recording of wound conditions without removing the dressing, providing real‐time alerts for abnormalities to reduce potential harm.^[^ ^487^ ^]^ Despite the evolution of wound dressings from single‐function to multifunctional designs, these advances have largely remained simple and one‐time‐use. Given the complexity of wound healing and the continuous changes in wound parameters, comprehensive strategies are needed for effective wound management and monitoring.^[^ ^463^ ^]^ The rapid development of smart flexible electronics and lightweight wearable devices in recent years has led to the creation of various stretchable, conductive electronic skins,^[^ ^488^ ^]^ which have proven effective for wound monitoring and management. Concurrently, advancements in wound dressings with integrated diagnostic and therapeutic functions, particularly those based on EEN scaffolds, have also progressed (Figure 5).

**Figure 12:**
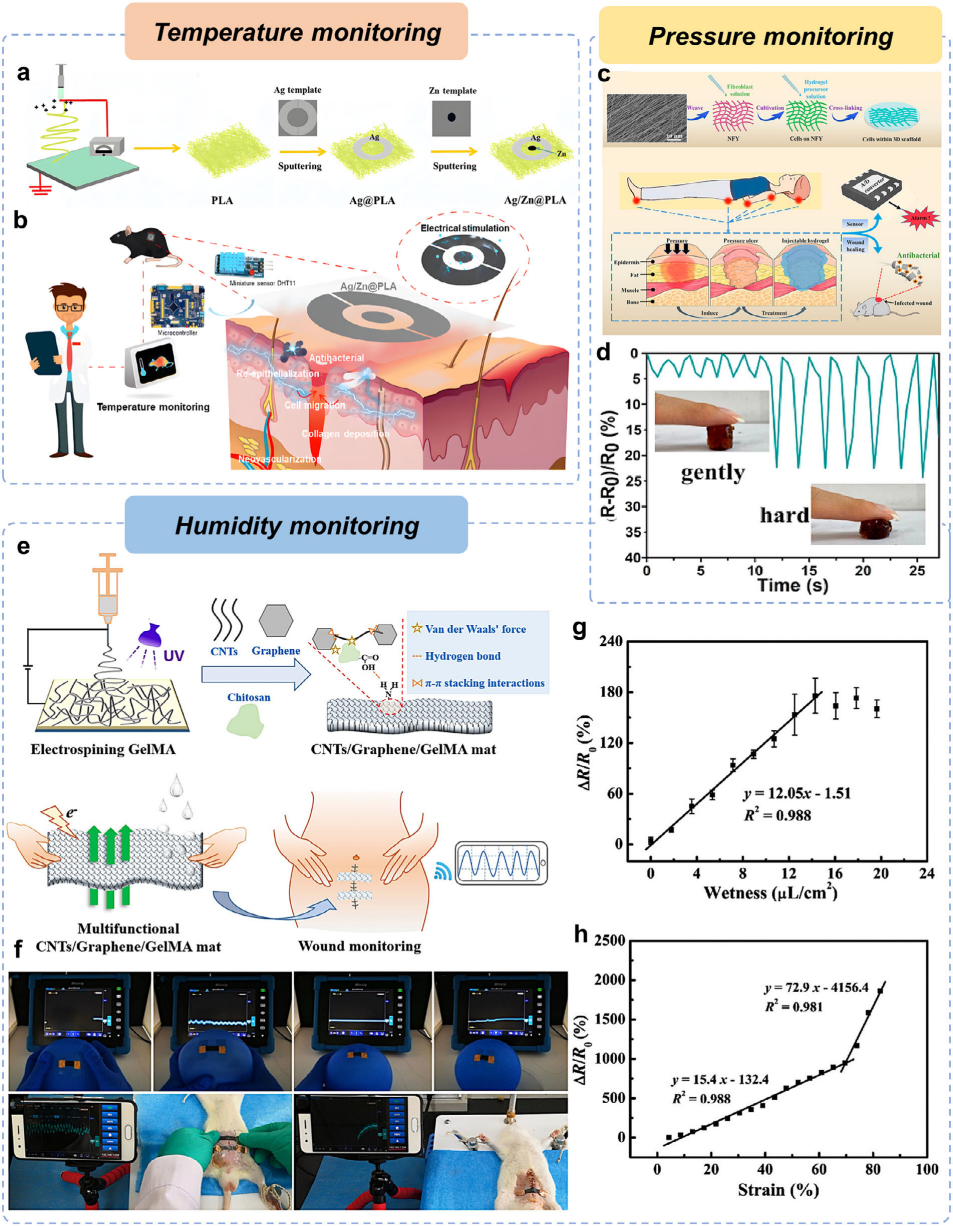
EEN scaffolds for wound monitoring. a) Schematic illustration of the fabrication process of the Ag/Zn@PLA dressing. b) Illustration of the possible wound healing promoting mechanism of Ag/Zn@PLA with miniature temperature sensor. Reproduced with permission.^[^ ^491^ ^]^ Copyright 2023, The Royal Society of Chemistry. c) Illustration of the fabrication of a 3D hybrid scaffold with aligned nanofiber yarns (NFY) embedded in injectable hydrogels and monitoring the pressure on the wound in real‐time. d) The amplitude of corresponding signals for different levels of pressure. Reproduced with permission.^[^ ^492^ ^]^ Copyright 2022, Elsevier Ltd. e) Schematic of the preparation process of CNTs/graphene/GelMA mat as an e‐skin and its application for wound monitoring. f) Application demo of CNTs/graphene/GelMA mat on a balloon and the belly of a rat for wound monitoring. g) The linear relationship between ΔR/R0 and wetness from 0 to 0.2 µL mm−2. h) Two linear relationships between ΔR/R0 and various strains in the ranges of 0–70% and 70–85%. Reproduced with permission.^[^ ^38^ ^]^ Copyright 2022, Elsevier Ltd.

Changes in wound temperature are important indicators of inflammation and infection.^[^ ^489^ ^]^ Monitoring temperature provides crucial information related to healing, such as local blood flow, oxygenation, wound infection, and inflammation.^[^ ^490^ ^]^ As technology advances, temperature monitoring is being increasingly incorporated into wound care, offering more precise and personalized treatment options for patients. Yang and his team sputtered Ag/Zn electrodes onto the surface of PLA nanofiber membranes to create an electroactive wound dressing (Ag/Zn@PLA) and integrated a miniature DHT11 sensor (**Figure** **12**a,b), allowing for real‐time temperature monitoring and treatment at the wound site.^[^ ^491^ ^]^

Pressure ulcers, commonly referred to as bedsores, result from sustained pressure on the skin and subcutaneous tissue, which impairs blood circulation, causing ischemia, and ulceration, and potentially leading to necrosis of the affected tissue.^[^ ^492^ ^]^ Pressure ulcers are generally considered preventable, with prevention strategies being the primary focus in clinical settings. Standard clinical care involves regular repositioning and the use of pressure‐relieving mattresses,^[^ ^493^ ^]^ though these methods lack quantitative monitoring capabilities. As the underlying cause of pressure ulcers is prolonged excessive localized pressure,^[^ ^494^ ^]^ monitoring changes in skin stress is critical for prevention. And then, Qiu and his team developed a 3D hybrid scaffold with conductive and antibacterial properties, designed for both monitoring and repairing chronic wounds (Figure 12c,d).^[^ ^492^ ^]^ This scaffold was constructed by encapsulating a network of nanofiber yarns (NFY) within an injectable hydrogel, where ions were incorporated to enhance conductivity, thus enabling its potential use as a flexible strain sensor for precise wound pressure monitoring. Additionally, the scaffold promotes cell elongation and alignment, effectively mimicking the ECM. However, both studies lacked in vivo monitoring experiments, casting doubt on the system's long‐term effectiveness and stability, which remains a notable limitation in current research.

Wound moisture is another crucial determinant in the healing process.^[^ ^495^ ^]^ Maintaining an optimal balance of wound moisture is essential, as both excessive and insufficient humidity can impede the healing process.^[^ ^496^ ^]^ Monitoring humidity levels within the wound environment provides critical insights into the wound's condition, offering guidance for treatment decisions such as optimal dressing changes. To address this clinical need, Li et al. developed a novel porous, breathable, stretchable, and conductive CNTs/Graphene/GelMA mat with both strain and humidity sensing capabilities, utilizing electrospinning, ice templating, and in situ loading techniques (Figure 12e).^[^ ^38^ ^]^ This novel electronic skin was integrated with portable devices for real‐time wound monitoring and management. The application demonstration of CNTs/graphene/GelMA has been performed on a balloon and the abdomen of a rat for wound monitoring (Figure 12f). The moisture sensitivity coefficient of the structure is 12.05 (Figure 12g), while the gauge factors for strain in the 0–70% and 70–85% ranges are 15.4 and 72.9 (Figure 12h), respectively, aligning well with the mechanical properties of human skin.^[^ ^38^ ^]^ Notably, this study successfully achieved dual monitoring of both strain and humidity, making it particularly suitable for challenging wound types, such as those at joint areas and post‐cesarean section sites.

Wearable sensors and systems for wound monitoring are advancing rapidly. Electroactive electrospun fiber scaffolds, utilized as functional wound dressings, have been reported to monitor parameters such as temperature, humidity, and strain. However, research on additional biomarkers (e.g. pH, glucose, ions, and enzymes) and the integration of intelligence remain underdeveloped, which requires further investigation and advancement.

## 7 Conclusions and Outlook

Bioelectricity is essential in the regenerative microenvironment, allowing tissues to respond to external stimuli while regulating cellular fate.^[^ ^497^ ^]^ Electrotherapy has been widely implemented in clinical settings to treat various diseases, yielding promising outcomes.^[^ ^498^ ^]^ This progress offers novel insights and approaches for tissue engineering. The advent of electroactive electrospun fibrous scaffolds represents an innovative extension of traditional electrotherapy, marking substantial progress in wound healing. These scaffolds are primarily fabricated from electroactive biomaterials, including conductive and piezoelectric materials, through electrospinning techniques. They are engineered to replicate the electrical properties, topographical structures, and mechanical cues of tissue and cellular microenvironments.^[^ ^499^ ^]^ Through this mimicry, they regulate cell‐cell and cell‐biomaterial interactions,^[^ ^163^, ^500^ ^]^ modulate cellular processes such as migration, proliferation, and differentiation, inhibit bacterial growth to mitigate inflammation and infection, and promote angiogenesis and epithelialization, address multiple needs throughout the wound healing process.^[^ ^22^ ^]^

The primary advantage of electroactive electrospun fibrous scaffolds is their capacity to closely replicate the microenvironment of tissues and cells while offering precise control. This fosters optimal conditions for wound tissue repair. Additionally, electrospinning technology enables the customization of scaffold shape, fiber diameter, and structure to suit specific wound conditions. When integrated with the tunability of electroactive materials, these scaffolds can be tailored to address the diverse needs of various wound types. Research has demonstrated that various types of electroactive nanofiber scaffolds outperform non‐electroactive nanofiber scaffolds, traditional gauze, and commercial dressings in enhancing wound healing and providing antibacterial benefits. These scaffolds have evolved rapidly, displaying significant potential and advancements. Different wound types necessitate distinct electroactive nanofiber scaffolds; for instance, burn wounds are better suited to nanofiber composite electroactive scaffolds, whereas infected wounds benefit more from scaffolds with robust antibacterial properties and exudate management. Other common wounds may have less stringent requirements. Nonetheless, findings from cell and animal model studies are currently inadequate to fulfill medical device standards.

From a clinical perspective, the translation of electroactive electrospun fibrous scaffolds into wound healing applications is relatively easier compared to other tissue engineering fields. This has motivated our team to review research advancements in this area to guide future studies and accelerate the clinical application of wound healing technologies. However, this research remains in its early stages, focusing primarily on basic research aimed at developing biomimetic electro‐microenvironments and material designs that enhance wound healing. Translating these laboratory findings into clinical applications poses significant challenges and leaves substantial room for further exploration, highlighting the need for continued research on the unresolved issues that remain. The challenges to be solved in the future include:

- Transcellular types and tissues and antimicrobial mechanisms are explored. While the importance of electrical stimulation or EFs in wound healing is widely recognized, the molecular and cellular mechanisms underlying cellular and bacterial responses to these stimuli remain incompletely understood. Moreover, there is a notable lack of studies on the synergistic mechanisms of electroactive fiber scaffolds in the wound healing process. Existing studies have found that possible biological mechanisms include activating cellular calcium ion activity, regulating ROS, promoting the increased expression of Ki67, VEGF, and FGF‐2, and the initiation of the TGF‐β/Smad signaling pathway.^[^ ^22^, ^33^, ^40^, ^231^ ^]^ However, the interactions between cells/tissues and electroactive fiber scaffolds and their underlying mechanisms remain unclear. Furthermore, the interrelationships between electro‐structural and antimicrobial properties, and the mechanisms driving these interactions, remain underexplored. Future research can leverage advanced biological techniques (e.g., single‐cell and spatial multi‐omics, membrane potential, and ion flow detection techniques) to elucidate the interactions between cells/tissues, bacteria, and scaffolds. This will help identify universal design principles for electroactive biomaterials, scaffold structures, and electrical stimulation parameters across tissue, cellular, molecular, and genetic levels.
- The optimization of electrical parameters is crucial for enhancing the performance of electroactive fiber scaffolds. Electrical parameters encompass both the conditions of electrical stimulation (e.g., voltage, frequency, pulse width, and stimulation duration) and the intrinsic conductivity of the material. Although the optimization of external electrical stimulation parameters has been discussed previously, generalized conclusions have yet to be reached. Minor variations in the responses of different cell types (e.g., fibroblasts, endothelial cells, and macrophages) and bacterial species during wound healing may further complicate these outcomes. Additionally, wound healing may require specific scaffold conductivity, and the impact of heterogeneous or homogeneous spatial distribution of components in electroactive scaffolds on potential in vivo outcomes remains unexplored. When external electrical stimulation is combined with electroactive scaffolds, the complexity increases, and the interactive effects between the two require deeper exploration. Despite the complexity of optimization, studies focusing on identifying optimal parameters for different wound types are essential to facilitate their translation from the laboratory to clinical practice.
- Emphasizing the biocompatibility and long‐term safety of scaffold materials is essential. Despite the multifunctionality of electroactive electrospun fiber scaffolds, their biocompatibility and long‐term safety are critical to their success. Electroactive electrospun fiber scaffolds consist of two components: an electrostatically spun substrate and electroactive biomaterials. The substrate is typically composed of FDA‐approved synthetic polymers and some natural polymers, which are considered relatively safe. However, the biocompatibility and degradability of electroactive biomaterials in vivo remain controversial, highlighting the need for developing safer electroactive biomaterials. Additionally, limitations exist in the current methods for assessing the safety of electroactive biomaterials and the fate of their degradation products. Beyond traditional biocompatibility and degradability assessments, high‐throughput assays are anticipated to enable multifactorial parallel evaluation and selection.
- Standardizing and expanding animal models is essential for advancing preclinical research. Currently, wound animal models primarily focus on full‐thickness incisions, burns, and pressure ulcers. Full‐thickness incisions are further categorized into diabetic wounds and infected wounds, depending on the presence of diabetes and bacterial infection. However, existing studies have incorrectly classified diabetic and infected wounds as chronic wound models, which diverges from the true definition of chronic wounds—a discrepancy evident in the descriptions of model preparation methods. Furthermore, species differences must be considered, as studies on electroactive fiber scaffolds in wound healing predominantly utilize rats, largely due to their convenience and cost‐effectiveness. However, significant differences between rats and humans in skin structure, immune response, and metabolic rate can limit the translatability of experimental results to clinical practice. Thus, large animal models, such as pigs, which more closely resemble the structural and physiological properties of human skin, should be prioritized in future studies. Moreover, the limited studies on animal models of burns and pressure ulcers hinder the exploration of electroactive fiber scaffolds in diverse wound types, thereby reducing their clinical applicability. This gap warrants further attention. Standardizing preclinical research protocols is essential to expedite the transition to clinical trials.
- Developing an intelligent stent strategy with dual functions of monitoring and treatment is essential for future advancements. Currently, research on EEN scaffolds in wound healing remains focused on either treatment or monitoring functions. However, a dual‐function strategy that integrates both diagnosis and treatment aligns more closely with clinical needs and represents the future development trend of functional dressings. Furthermore, the development of wearable, miniaturized, and self‐powered fiber scaffolds is gaining importance due to the limitations of external power sources, such as structural rigidity, large size, and non‐portability. Piezoelectric biomaterials play a pivotal role in this process, and optimizing their piezoelectric properties remains an area of active exploration. Additionally, exploring other forms of wireless stimulation, such as photoelectric conversion, to effectively harness both light and electrical exogenous stimuli for synchronized treatment presents a promising strategy, although research in this area remains limited. With advances in wireless transmission technologies (e.g., Bluetooth, near‐field communication, and radiofrequency identification), wearable sensors capable of monitoring wound biomarkers (e.g., pH, glucose, temperature, strain, exudates, inflammatory markers, and drug concentration) in situ and transmitting real‐time data have garnered significant attention. Developing intelligent scaffolds with dual functions—monitoring and treatment—by leveraging electroactive fiber scaffolds to achieve synchronized wound status information and remote treatment control is a key direction for future research. Research in this field may require the integration of artificial intelligence, the Internet of Things, and 5G technologies, alongside cross‐disciplinary collaboration among fields such as materials science, biomedicine, e‐informatics, and precision instrumentation. In summary, the future development of EEN scaffold strategies in skin tissue engineering is likely to focus on portability, intelligence, personalization, and customization. This trajectory will drive the emergence of innovative solutions in tissue engineering and clinical therapeutics, opening new possibilities for enhanced tissue repair.
- Balancing cost, ease of use, safety, and effectiveness will be a major challenge in the clinical translation of EEN scaffolds. From a doctor's perspective, patients generally prefer treatment options that are simple, convenient, cost‐effective, and efficacious. Reducing the threshold for patient usage will help broaden the clinical applicability of this technology. Moreover, stent‐related dressings are typically classified as consumables and need to be applied continuously throughout the entire wound‐healing process. However, medical insurance policies cannot fully cover the costs, thus increasing the financial burden on patients. To address this issue, optimizing the cost structure and developing cost‐effective materials that reduce production costs while prolonging the effective use time of dressings will be essential strategies. In conclusion, by achieving a comprehensive balance between cost control, ease of use, safety, and effectiveness, this technology will enhance its competitiveness in future clinical applications.

In conclusion, the electroactive electrospun nanofiber scaffold strategy holds significant research value and application potential in wound healing, owing to its capacity to create a biomimetic regenerative electrical microenvironment and enhance tissue regeneration potential. Although challenges and limitations remain, a comprehensive exploration of the biological effects of EFs and electroactive scaffolds, coupled with ongoing technological advancements, is expected to position electroactive nanofiber scaffolds as a key player in skin tissue regeneration and a forerunner in clinical translation.

## Conflict of Interest

The authors declare no conflict of interest.
